# Aids to management of headache disorders in primary care (2nd edition) on behalf of the European Headache Federation and *Lifting The Burden*: the Global Campaign against Headache

**DOI:** 10.1186/s10194-018-0899-2

**Published:** 2019-05-21

**Authors:** T. J. Steiner, R. Jensen, Z. Katsarava, M. Linde, E. A. MacGregor, V. Osipova, K. Paemeleire, J. Olesen, M. Peters, P. Martelletti

**Affiliations:** 1 Department of Neuromedicine and Movement Science, NTNU Norwegian University of Science and Technology, Edvard Griegs Gate, Trondheim, Norway; 2 Division of Brain Sciences, Imperial College London, London, UK; 3 Danish Headache Centre, Department of Neurology, University of Copenhagen, Rigshospitalet Glostrup, Glostrup, Denmark; 4 Department of Neurology, Evangelical Hospital Unna, Unna, Germany; 5 Medical Faculty, University of Duisburg-Essen, Essen, Germany; 6 Norwegian Advisory Unit on Headache, St. Olavs Hospital, Trondheim, Norway; 7 Centre for Neuroscience and Trauma, Blizard Institute of Cell and Molecular Science, Barts and the London School of Medicine and Dentistry, London, UK; 8 Research Department of Neurology, First “I. Sechenov” Moscow State Medical University, Moscow, Russian Federation; 9 Research Center for Neuropsychiatry, Moscow, Russian Federation; 10 Department of Neurology, Ghent University Hospital, Ghent, Belgium; 11 Health Services Research Unit, Nuffield Department of Population Health, University of Oxford, Oxford, UK; 12 Department of Clinical and Molecular Medicine, Sapienza University, Rome, Italy; 13 Regional Referral Headache Centre, Sant’Andrea Hospital, Rome, Italy

**Keywords:** Headache disorders, Migraine, Tension-type headache, Cluster headache, Medication-overuse headache, Trigeminal neuralgia, Persistent idiopathic facial pain, Classification, Diagnosis, Management, Primary care, Guidelines, Red flags, Patient information leaflets, Follow-up, Instruments, Outcome measures, Headache calendar, Headache diary, Burden of disease, Translation, Global Campaign against Headache, European Headache Federation

## Abstract

The *Aids to Management* are a product of the Global Campaign against Headache, a worldwide programme of action conducted in official relations with the World Health Organization. Developed in partnership with the European Headache Federation, they update the first edition published 11 years ago.

The common headache disorders (migraine, tension-type headache and medication-overuse headache) are major causes of ill health. They should be managed in primary care, firstly because their management is generally not difficult, and secondly because they are so common. These *Aids to Management*, with the *European principles of management of headache disorders in primary care* as the core of their content, combine educational materials with practical management aids. They are supplemented by translation protocols, to ensure that translations are unchanged in meaning from the English-language originals.

The *Aids to Management* may be individually downloaded and, as is the case for all products of the Global Campaign against Headache, are available without restriction for non-commercial use.

**Electronic supplementary material:**

The online version of this article (10.1186/s10194-018-0899-2) contains supplementary material, which is available to authorized users.

## Preface

Medical management of headache disorders does not, for the vast majority of people affected by them, require specialist skills or investigations. It can and should be based in primary care [1].

Nonetheless, non-specialists throughout Europe may have received limited training in the diagnosis and treatment of headache [1]. This publication combines educational materials with practical management aids. It is a product of the Global Campaign against Headache, a worldwide programme of action for the benefit of people with headache conducted by the UK-registered non-governmental organization *Lifting The Burden* (LTB) in official relations with the World Health Organization [2].

*Aids to management of headache disorders in primary care (2nd edition)* updates the first edition, published 11 years ago [3]. The content has been put together by a writing group of experts convened by LTB in collaboration with the European Headache Federation (EHF). It has undergone review by a wider consultation group of headache experts, including representatives of the member national societies of EHF, primary-care physicians from eight countries of Europe, and lay advocates from member organisations of the European Headache Alliance. While the focus is Europe, these aids may be useful to a much wider population.

The *European principles of management of headache disorders in primary care*, laid out in 14 sections, are the core of the content. Each section is stand-alone and may be separately down-loaded (*Management of migraine* is in four separate parts), in order to act as a practical management aid as well as an educational resource.

There is a set of additional practical management aids. An abbreviated version of *the International Classification of Headache Disorders, 3rd edition* [4], provides diagnostic criteria for the relatively few headache disorders relevant to primary care. A headache diary further assists diagnosis and a headache calendar supports follow-up. A measure of headache impact, the HALT-90 Index, can be employed in pre-treatment assessment of illness severity. Its derivative, the HALT-30 Index, may be more useful in follow-up, along with the HURT questionnaire, an outcome measure designed to guide follow-up. Any of seven information leaflets may be offered to patients to improve their understanding of their headache disorders and their management. Each of these may also be separately down-loaded.

LTB and EHF offer these aids for use without restriction for non-commercial purposes, as is the case for all products of the Global Campaign against Headache [2]. We hope for benefits for both physicians and patients. For the former, the aids have been designed expressly to assist primary-care physicians in delivering appropriate care more efficiently and more cost-effectively for a group of disorders that, collectively, are very common and very disabling. For the latter, there should be better outcomes for the many people with headache who need medical treatment.

The materials will need translating into many languages. Among the supplementary materials are translation protocols developed by LTB to ensure that translations as far as possible are unchanged in meaning from the English-language originals.

**Table:** 

**TJ Steiner**	**P Martelletti**
Global Campaign Director	President
*Lifting The Burden*	European Headache Federation

## European principles of management of headache disorders in primary care

### Introduction

Headache disorders are the second-highest cause of disability in Europe [4]. Three of these disorders (migraine, tension-type headache [TTH] and medication-overuse headache [MOH]) are important in primary care because they are common and responsible for almost all burden attributed to headache [4, 5]. Management of these belongs largely in primary care [1].

A fourth headache disorder, cluster headache, is also important because, although not common, it is extremely painful. It is treatable in specialist care, but is very often misdiagnosed, and consequently not referred, over many years. Also requiring specialist management and therefore important to recognise are trigeminal neuralgia and persistent idiopathic facial pain.

The management of migraine, TTH and MOH is in most cases not difficult. The purpose of these principles is to help primary-care physicians correctly diagnose these few disorders, manage them well when they can, recognise warnings of serious headache disorders and refer for specialist care whenever necessary.

### Development process

#### Stakeholder involvement

These principles were developed by *Lifting The Burden* (LTB) in collaboration with the European Headache Federation (EHF) as a product of the Global Campaign against Headache.

The **writing group** (TJS, RJ, ZK, ML, EAM, VO, KP and PM) were headache specialists from Belgium, Denmark, Germany, Italy, Norway, Russian Federation, Sweden and United Kingdom (UK).

The **consultation group**, who undertook review, were primary care physicians from the same countries, members of the national headache societies within EHF (representing Albania, Austria, Belgium, Belarus, Bulgaria, Croatia, Czech Republic, Denmark, Estonia, Finland, France, Germany, Georgia, Greece, Hungary, Iran, Israel, Italy, Lithuania, Moldova, Montenegro, Morocco, The Netherlands, Norway, Poland, Portugal, Romania, Russian Federation, Serbia, Slovakia, Slovenia, Spain, Sweden, Switzerland, Turkey and UK), and patient representatives and advocates consulted through the Board of the European Headache Alliance.

All active contributors to the review are named in the acknowledgements at the end of this article.

#### Rigour of development

The development process was organised in four stages:

1. review by the writing group of all treatment guidelines or recommendations in use in Europe and published or otherwise available in English (from Austria, Belgium, Croatia, Denmark, Finland, France, Germany, Hungary, Italy, The Netherlands, Portugal, Spain, Switzerland, UK and European Federation of Neurological Societies [the last written by experts from Belgium, Denmark, Germany, Greece, Hungary, Italy, Sweden, Switzerland and UK]);
2. harmonisation by selection, through expert consensus within the writing group, of whichever recommendations within these carried greatest weight (evidence-based recommendations were always preferred to those without explicit supporting evidence; discordance between recommendations was resolved through reference to original evidence or, where this was lacking, through expert consensus);
3. review by the consultation group;
4. final editing by the writing group in the light of all comments.

#### Editorial independence

EHF was the sole funding body supporting development of these principles. Potential competing interests are declared at the end of this article.

These principles make no recommendations that favour one proprietary medication over another unless they are clearly evidence-based.

### The principles

To facilitate use in routine practice, these principles are designed as and additionally set out in 14 stand-alone management aids (see below). For this reason, there is deliberate repetition of some content between them.

They are likely to be most useful if read through at least once in their entirety, then used for reference.

The principles are in **three parts**:

**Guides to diagnosis** (some elements of these will need to be assimilated into routine practice, whereas others can serve as check lists and *aide-mémoires*).

- Headache as a presenting complaint (Additional file 1)
- Typical features of the headache disorders relevant to primary care (Additional file 2)
- Diagnosis of headache disorders (Additional file 3)

**Guides to management** (these are information sources to be referred to once the diagnosis has been made; they include guidance on information to patients (Additional file 5)).

- General aspects of headache management (Additional file 4)
- Advice to patients (Additional file 5)
- Management of migraine (Additional files 6, 7, 8 and 9)
  - Acute or symptomatic management of episodic migraine (Additional file 7)
  - Prophylactic management of episodic migraine (Additional file 8)
  - Management of chronic migraine (Additional file 9)
- Management of tension-type headache (Additional file 10)
- Management of cluster headache (Additional file 11)
- Management of medication-overuse headache (Additional file 12)
- Management of trigeminal neuralgia and persistent idiopathic facial pain (Additional file 13)

**Guide to referral** (a reference and reminder).

- Headache management in primary care: when to refer (Additional file 14)

#### Clarity and presentation

The aim was to give straightforward and easily followed guidance to primary-care physicians, who were assumed to be non-expert.

The emphasis was on unambiguous advice. Nevertheless, because availability and regulatory approval of drugs and reimbursement policies vary from country to country, different possible options are set out wherever appropriate.

All guidance is evidence-based but, for clarity of presentation, the evidence is not laid out.

#### Applicability

These principles assume that headache services are developed and adequately resourced in all countries in Europe, even though this is not the case at present [3]. Separate initiatives by LTB and EHF are being undertaken to support better organisation of headache services in all countries in Europe [2].

These principles, now in their second edition, will be reviewed from time to time by the writing group.

### Guides to diagnosis

#### Headache as a presenting complaint

This guide can be separately downloaded (Additional file 1).

Most people have occasional headache. This is a **symptom**, which many people regard as “normal”. Headache becomes a problem at some time in the lives of about 40% of adults and lesser but still substantial proportions of children and adolescents. These people have a **headache disorder**.

The International Classification of Headache Disorders (ICHD) [4] recognises over 200 headache disorders, and divides them into three groups (see 3.2 *Diagnostic criteria for headache disorders in primary care: The International Classification of Headache Disorders, 3rd edition (ICHD-3) – abbreviated form* (also, Additional file 15)).

- ▪ **Primary headache disorders** include migraine, tension-type headache (TTH) and cluster headache, all of which are important in primary care (Table 1).
- ▪ **Secondary headache disorders** have another causative disorder underlying them; therefore, the headache occurs in close temporal relation to the other disorder, and/or worsens or improves in parallel with worsening or improvement of that disorder. These associations are keys to their diagnosis. Secondary headache disorders include medication-overuse headache (MOH), also important in primary care (Table 1).
- ▪ **Painful cranial neuropathies and other facial pains** include two disorders, trigeminal neuralgia and persistent idiopathic facial pain, that need to be recognised in primary care.

**Table 1:** The headache disorders of particular importance in primary care

Migraine	• Usually episodic, occurring in 15–25% of the general population, in women more than men in a ratio of up to 3:1; • A chronic type is recognised, with headache occurring on more days than not
Tension-type headache	• Usually episodic, affecting most people from time to time but, in at least 10%, recurring frequently; • In up to 3% of adults and some children it is chronic, occurring on more days than not
Cluster headache	• Extremely intense and frequently recurring but short-lasting headache attacks, affecting up to 3 in 1000 men and up to 1 in 2000 women
Medication-overuse headache	• A secondary headache, but occurring only as a complication of a pre-existing headache disorder, usually migraine or tension-type headache, present on most days (≥15 days/month) and affecting 1–2% of adults, women more than men, and about 0.5% of children and adolescents

A patient may have **more than one of these disorders** concomitantly.

##### Which headaches should be managed where?

Four headache disorders are of particular importance in primary care (Table 1). All have a neurobiological basis. They are variably painful and disabling, but all may cause lost productivity and impair quality of life. Collectively they are the second highest cause of disability worldwide [5], and therefore very costly.

- ▪ **Migraine, TTH and MOH** can and should, almost always, be managed well in primary care.
  - ▪ Specific advice on each of these is given below (also, Additional files 6, 7, 8, 9, 10 and 12).
- ▪ The exception is **chronic migraine**. This uncommon type should be recognised in primary care, but it is difficult to treat and likely to require specialist management.
  - ▪ Specific advice on this is given below (also, Additional file 9).
- ▪ **Cluster headache** should be diagnosed in primary care because it is easily recognisable, but referred for specialist management.
  - ▪ Specific advice on this is given below (also, Additional file 11).
- ▪ Among painful cranial neuropathies and other facial pains are **trigeminal neuralgia** and **persistent idiopathic facial pain**. These should be recognised when present, but require specialist management.
  - ▪ Specific advice on each of these is also given below (also, Additional file 13).
- ▪ Any headache **not responding satisfactorily** to management in primary care should also be referred for specialist management.
- ▪ Of the large number of other secondary headache disorders, **some are serious**. Overall these account for <1% of patients presenting with headache, but they **must be recognised**.
  - ▪ Advice on these is provided under 2.4.3 *Diagnosis of headache disorders* (also, Additional file 3).

More general advice on indications for referral to specialist management is set out under 2.6.1 *Headache management in primary care: When to refer* (also, Additional file 14).

#### Typical features of the headache disorders relevant to primary care

This guide can be separately downloaded (Additional file 2).

The **distinguishing features** of the important primary headache disorders are summarised in Table 2.

**Table 2:** Summary of features distinguishing the important primary headache disorders (NB: two or more of these disorders may occur concomitantly)

	Migraine	Tension type headache (TTH)	Cluster headache (CH)
Temporal pattern	Episodic migraine: Recurrent attack-like episodes, lasting from 4 h to 3 days; frequency often 1–2/month but variable from 1/year to 2/week or more; freedom from symptoms between attacks Chronic migraine: Episodicity lost: headache on ≥15 days/month, having migrainous features on ≥8 days/month	Frequent episodic TTH: Recurrent attack-like episodes lasting hours to a few days; 1–14 days affected per month; freedom from symptoms between attacks Chronic TTH: ≥15 days affected per month (often daily and unremitting)	Episodic CH: Frequent (typically ≥1 daily) short-lasting attacks (15–180 min): • Recurring in bouts, usually once or sometimes twice a year, which are typically of 6–12 weeks’ duration; • Then remitting for ≥3 months Chronic CH: Similar, but without such remissions between bouts
Typical headache characteristics	Often unilateral; often pulsating	Can be unilateral but more often generalised; may spread to the neck; typically described as pressure or tightness	Strictly unilateral (although side-shifts occur occasionally), around the eye or over the temple
Headache intensity	Typically moderate to severe	Typically mild to moderate	Extremely severe
Associated symptoms	Aura (in a minority of attacks); often nausea and/or vomiting; often photo- and/or phonophobia	Frequent episodic TTH: None typical; mild photophobia or phonophobia may occur Chronic TTH: Sometimes mild nausea, but not vomiting	Strictly ipsilateral autonomic features: • Any or all of red and/or watering eye, running or blocked nostril, ptosis
Reactive behaviour	Avoidance of physical activity (maybe bed rest); preference for dark and quiet	None specific	Marked agitation: cannot lie still during attacks

##### Migraine

Migraine is typically a **moderate-to-severe headache** accompanied by **nausea, vomiting** and **sensitivity to light and/or noise**. It is more prevalent among women than among men.

Migraine is usually episodic, occurring in attacks lasting hours to a few days. The **two principal types** are migraine without aura and the less common migraine with aura. One patient may have both types. There is also an uncommon chronic type.

***Migraine without aura***

**Adults** with this disorder describe:

- ▪ recurrent episodic **moderate or severe headaches** which, *typically but not always*:
  - ▪ are **unilateral** and/or **pulsating**;
  - ▪ last (when untreated) from 4 h to 3 days;
  - ▪ are **associated with**:
    - ▪ nausea and/or vomiting;
    - ▪ photophobia, phonophobia and sometimes osmophobia;
  - ▪ are aggravated by routine physical activity, and **disabling**;
  - ▪ and during which they limit their activity and prefer **dark and quiet**;
- ▪ **freedom** from these symptoms **between attacks**.

In **children:**

- ▪ attacks may be shorter-lasting;
- ▪ headache is more often bilateral and less often pulsating;
- ▪ gastrointestinal disturbance is often more prominent.

***Migraine with aura***

This type affects about one third of people with migraine, although only a minority of these experience aura symptoms with every attack. It is characterised by:

- ▪ **aura** preceding or less commonly accompanying headache and consisting of **one or more neurological symptoms** (see Table 3)
- ▪ **headache** that is similar to migraine without aura, or may be rather featureless.

**Table 3:** Symptoms of aura (developing gradually over ≥5 min and usually resolving within 60 min)

Typical	• **Visual symptoms** (occurring in >90% of auras): usually a slowly-enlarging scintillating scotoma (patients may draw a jagged crescent if asked); *and/or* • **Unilateral paraesthesiae** and/or numbness of hand, arm and/or face
Less usual	• Brainstem symptoms (*eg*, vertigo, tinnitus, diplopia, ataxia); • Speech and/or language disturbances
Rare	• Motor weakness

**Typical aura without headache** may occur in patients with a past history of migraine with aura.

***Chronic migraine***

This highly disabling migraine type develops, in a small minority of patients, from episodic migraine. Over time, attacks become more frequent, with **loss of clear periodicity**. Simultaneously, the specific characteristics of migraine become less pronounced.

Chronic migraine is **not simply more frequent migraine**. It is essentially characterised by:

- ▪ **headache** occurring on **≥15 days/month** for at least 3 months which:
- ▪ on **≥8 days/month** meets diagnostic criteria for **migraine** (or responds to migraine-specific drug treatment);

and often **complicated by**:

- ▪ depression and/or anxiety;
- ▪ low back and/or neck pain;
- ▪ medication overuse.

Transformation of episodic migraine to a chronic headache disorder is very often **causally associated with medication overuse**:

- ▪ the correct diagnosis is then **medication-overuse headache** (MOH);
- ▪ chronic migraine and MOH are **not mutually exclusive** but, when medication is being overused, it may be that only MOH and not chronic migraine is present.

##### Tension-type headache (TTH)

This disorder is typically a **mild-to-moderate headache** of highly variable frequency and duration, **without associated symptoms** or the specific features of migraine. It tends to be more common in women than in men.

It has **three types**. Infrequent episodic TTH, occurring less than once a month, is not medically important. The others are frequent episodic TTH and chronic TTH.

***Frequent episodic tension-type headache***

- ▪ occurs in attack-like episodes on **1–14 days/month**, each lasting hours to a few days;
- ▪ can be unilateral but is more often generalised;
- ▪ is typically described as **pressure or tightness** like a vice or tight band around the head, often spreading to the neck;
- ▪ lacks the associated symptom complex of migraine.

***Chronic tension-type headache***

This type has features similar to those of frequent episodic TTH but:

- ▪ occurs by definition on **≥15 days/month for >3 months**, and may be daily and unremitting;
- ▪ may be associated with mild nausea.

##### Cluster headache

This disorder is characterised by frequently recurring, localised, **short-lasting** but **extremely severe headache** accompanied by a set of very recognisable **autonomic symptoms**. It affects men three times as commonly as women.

It **should never be missed**. It demands accelerated specialist referral, investigation and treatment.

Cluster headache occurs in attacks, which *very typically*:

- ▪ are characterised by headache of excruciating intensity, which is
  - ▪ strictly **unilateral and localised** around the eye or over the temple;
  - ▪ accompanied by highly characteristic and strictly ipsilateral **autonomic features**, including any or all of:
    - ▪ red and watering eye;
    - ▪ running or blocked nostril;
    - ▪ ptosis;
  - ▪ associated with marked **agitation** (the patient, unable to stay in bed, paces the room, even going outdoors);
- ▪ occur **once or more daily**, very often at night (causing awakening);
- ▪ last **15–180 min** (commonly 30–60).

Cluster headache has **two subtypes**, episodic and (less common) chronic.

***Episodic cluster headache***

- ▪ occurs in **bouts** (clusters) of recurring attacks, typically **once or twice a year**, which:
  - ▪ are of 6–12 weeks’ duration (but may be longer);
  - ▪ then remit until the next cluster, at least 3 months later.

***Chronic cluster headache***

- ▪ persists, still as recurring attacks but **without remissions**, or with remissions of <3 months;
- ▪ may develop from and/or revert to episodic cluster headache.

##### Medication-overuse headache (MOH)

This is one of the syndromes characterised by **headache occurring on ≥15 days/month**. It is often daily, but variable in site, intensity and character. It greatly impairs quality of life. It is more common in women.

Medication-overuse headache:

- ▪ occurs **daily or near-daily** (by definition on ≥15 days/month);
- ▪ is present – and often at its worst – early in the morning;
- ▪ is **causally associated** with regular use, over >3 months, of:
  - ▪ non-opioid analgesics on ≥15 days/month, and/or
  - ▪ opioids, ergots or triptans, or any combination of these, on ≥10 days/month.

MOH is an **aggravation of a prior headache** (usually migraine or tension-type headache) by chronic overuse of medication taken to treat headache or other pain. A history can usually be elicited of increasingly frequent and difficult-to-treat headache episodes, with increasing medication use, over months to many years.

**All acute headache medications** may have this effect. Frequency, regularity and duration of intake are important determinants of risk.

MOH tends to worsen initially when attempts are made to reduce consumption of the overused medication(s), but in most cases improves within 2 months after overuse is stopped.

##### Important causes of facial pain

Many causes of facial pain may bring patients to GPs. Two in particular, although not common, require recognition.

***Trigeminal neuralgia (TN)***

This disorder presents as recurrent, unilateral, **brief but severe, electric-shock-like pains** in the distribution of the trigeminal nerve, abrupt in onset and termination and often triggered by innocuous stimuli.

- ▪ Trigeminal neuralgia (TN) affects women twice as commonly as men, and mostly those above 50 years of age (but may occur in younger people). It has no other known risk factors.
- ▪ It is often associated with neurovascular compression of the trigeminal nerve close to its point of entry to the brainstem (**classical trigeminal neuralgia**).
- ▪ TN is one of the **most painful disorders**, demanding **accelerated specialist referral**, investigation and treatment.
- ▪ **MRI** of the brain (including brainstem) is essential.
  - ▪ This may demonstrate neurovascular compression, but is required in any case to exclude secondary causes that give rise to pains indistinguishable from classical TN. These occur more often in younger people.

*Classical trigeminal neuralgia:*

- ▪ occurs in **bouts of repeated**, stabbing or **electric-shock-like pains** in the distribution of one or more divisions of the trigeminal nerve (usually the 2nd and/or 3rd), which are:
  - ▪ **excruciating**;
  - ▪ of **sudden onset**;
  - ▪ highly characteristically **triggered** by sensory stimuli to the affected side of the face (touching, washing, applying make-up) or by talking, eating, chewing, drinking or smoking;
  - ▪ **short-lasting** (from less than a second up to 2 min);
  - ▪ **strictly unilateral**, and not switching side between bouts;
  - ▪ often **serial**, with up to hundreds of pain paroxysms during 1 day;
- ▪ may also feature a **constant aching pain** between attacks, in the affected area, of moderate intensity.

Bouts may remit completely for months or years in an unpredictable pattern. Otherwise, treatment may require surgical decompression.

*Secondary trigeminal neuralgia*

- ▪ has characteristics similar to classical trigeminal neuralgia, but is secondary to another disorder (usually cerebellopontine angle tumour, AV-malformation or multiple sclerosis).

***Persistent idiopathic facial pain (PIFP)***

Previously termed “atypical facial pain”, this disorder presents as variable but persistent, **poorly localized facial and/or oral pain**. It is more common in women.

Persistent idiopathic facial pain (PIFP):

- ▪ is **dull**, aching or nagging;
- ▪ **recurs daily** for >2 h and **persists** over >3 months;
- ▪ is **unassociated** with neurological deficit;
- ▪ is aggravated by **stress**.

PIFP is associated with high levels of **psychiatric comorbidity** and psychosocial disability, and difficult to manage. It usually requires **specialist referral**. However:

- ▪ patients are often referred for exclusion of sinus and dental problems, then returned untreated to primary care;
- ▪ referral to a specialist clinic with a pain management programme is preferable.

**Temporomandibular disorder** (TMD) is in the differential diagnosis of PIFP. This is itself a very complex problem:

- ▪ the pain associated with TMD is usually most prominent in the pre-auricular areas of the face, masseter muscles and/or temporal regions;
- ▪ there is significant overlap between TMD and tension-type headache and jaw, dental and bite disorders.

#### Diagnosis of headache disorders

This guide can be separately downloaded (Additional file 3).

The universally accepted basis for the diagnosis of any headache is the International Classification of Headache Disorders [4], an abbreviated version of which is included in these aids (3.2 *Diagnostic criteria for headache disorders in primary care: The International Classification of Headache Disorders, 3rd edition (ICHD-3) – abbreviated form* (also, Additional file 15)). In all health-care settings, diagnostic practice should employ ICHD terminology.^1^

##### Differential diagnosis of the headache disorders relevant to primary care

Diagnosis of episodic migraine or episodic tension-type headache requires multiple attacks; neither diagnosis should be made after a first attack without exclusion of other disorders.

- ▪ Each of the primary headaches is in the differential diagnosis of each of the others.
- ▪ Medication-overuse headache is in the differential diagnosis of chronic migraine or chronic tension-type headache.
- ▪ The distinguishing features of these are described above (2.4.2 *Typical features of the headache disorders relevant to primary care*) (also in Additional file 2).
- ▪ Otherwise, the differential diagnosis potentially includes **a small number of serious secondary headaches that are important to recognise** (see *Warning features in the history or on examination*, below).

***Taking a diagnostic history***

The **history is all-important** in the diagnosis of the primary headache disorders and of medication-overuse headache. There are no useful diagnostic tests.

Table 4 indicates diagnostic questions to elicit any that may be present of the features described above (2.4.2 *Typical features of the headache disorders relevant to primary care*) (also in Additional file 2).

**Table 4:** Diagnostic questions to ask in the history

How many different headaches types does the patient have? A separate history is needed for each.	
Time questions	• Why consulting now? • How recent in onset? • How frequent, and what temporal pattern (episodic or daily and/or unremitting)? • How long do headache episodes last?
Character questions	• Manner and speed of headache onset (abrupt, progressive over minutes, hours, days or longer)? • Intensity of pain? • Nature and quality of pain? • Site and spread of pain? • Associated symptoms?
Cause questions	• Predisposing and/or trigger factors? • Aggravating and/or relieving factors? • Family history of similar headache?
Response questions	• What does the patient do during the headache? • How much is activity limited or prevented? • What medications are used, and how frequently?
State of health between attacks	• Completely well, or residual symptoms?

***Diagnostic diary***

A diary kept over a few weeks can be a very helpful diagnostic aid, clarifying the pattern and frequency of headaches and associated symptoms as well as medication use or overuse.

An example is included here, among the management aids (3.3.2 *Diary and calendar for use in primary care* (also, Additional files 16)).

***Warning features in the history***

The history should also elicit any warning features of a serious secondary headache disorder:

- ▪ **any new headache**, or a significant change in headache characteristics, should provoke a new diagnostic enquiry;
- ▪ **very frequent headache** should always lead to detailed enquiry into medication use, since overuse is a likely cause;
- ▪ in addition, there are a number of **specific warning features** (“red flags”) that may be elicited (Table 5).

**Table 5:** Specific warning features (“red flags”) in the history

Warning feature	What to beware of
Thunderclap headache (intense headache with “explosive” or abrupt onset)	Subarachnoid haemorrhage
Headache with atypical aura (duration >1 h, or including motor weakness)	TIA or stroke
Aura without headache in the absence of a prior history of migraine with aura	TIA or stroke
Aura occurring for the first time in a patient during use of combined hormonal contraceptives	Risk of stroke (requires discontinuation)
New headache within 3 months of head trauma	Subdural haematoma
Progressive headache, worsening over weeks or longer	Intracranial space-occupying lesion
Headache aggravated by postures or manoeuvres that raise intracranial pressure	Intracranial space-occupying lesion
Headache brought on by coughing, exercise or sexual activity	Intracranial space-occupying lesion
Mild-to-moderate progressive or recurrent headache with irritability, dizziness (light-headedness), nausea and/or tiredness and confusion	Carbon monoxide poisoning
Headache associated with unexplained focal neurological symptoms or with epileptic seizures	Suggests secondary headache
Headache associated with change in memory or personality	Suggests secondary headache
Headache associated with weight-loss	Suggests secondary headache
New headache in a patient older than 50 years	Temporal arteritis or intracranial tumour
New headache in a patient with a history of cancer or immunodeficiency (including HIV infection)	Likely to be secondary headache
New headache in a patient with a history of polymyalgia rheumatica	Temporal (giant cell) arteritis
New headache in a patient with a family history of glaucoma	Glaucoma

***Physical examination of headache patients***

Migraine, tension-type headache, cluster headache and medication-overuse headache are diagnosed **solely on history**. Signs are present in cluster headache patients when seen during attacks (red and/or watering eye, running or blocked nostril and/or ptosis ipsilateral to the pain).

- ▪ **Blood pressure** measurement in all cases is good practice.
- ▪ Physical examination is **mandatory** when the **history is suggestive of secondary headache**, and then may elicit warning signs (Table 6).

**Table 6:** Warning features on examination, when associated with headache

Warning feature	What to beware of
Otherwise unexplained pyrexia	Meningitis
Neck stiffness	Meningitis or subarachnoid haemorrhage
Focal neurological signs	Secondary headache
Disorders of consciousness or memory	
Change in personality	
Weight-loss or poor general condition	

***Investigation of headache patients***

- ▪ Routine blood tests as a screen for general health may be worthwhile in primary care.
- ▪ **Special investigations**, including neuroimaging, are **not indicated unless** the history or examination suggests headache may be secondary to another condition.

***Diagnostic caveats***

The following tend to be **greatly overdiagnosed**:

- ▪ **cervicogenic headache** (headache caused by a disorder of the cervical spine and its component bony, disc and/or soft tissue elements, usually but not invariably accompanied by neck pain);
- ▪ headache attributed to **arterial hypertension** (chronic arterial hypertension below 180/110 mmHg does not appear to *cause* headache);
- ▪ headache attributed to **refractive error** (rare in adults, although some evidence exists for it in children);
- ▪ headache attributed to “**sinusitis**” (a misdiagnosis commonly applied to migraine);
- ▪ **trigeminal neuralgia** (recurrent unilateral brief electric shock-like pains, abrupt in onset and termination, limited to the distribution of one or more divisions of the trigeminal nerve and triggered by innocuous stimuli);
- ▪ **occipital neuralgia** (paroxysmal shooting or stabbing pain in the posterior part of the scalp, in the distributions of greater, lesser and/or third occipital nerves).

### Guides to management

#### General aspects of headache management

This guide can be separately downloaded (Additional file 4).

The purpose of these principles of management is to provide guidance, while demonstrating that headache **management in most cases is not difficult**.

The following are generally important for all headache disorders managed in primary care.

##### Educating and reassuring patients

Many people with recurrent headache wrongly fear underlying disease, so education and **appropriate reassurance** should never be omitted.

Good treatment of patients with any headache disorder therefore begins with **explanations** of their disorder and the purpose and means of management.

- ▪ Explanation is a crucial element of **preventative management** in patients with migraine or frequent episodic tension-type headache, who are at particular risk of escalating medication consumption.
- ▪ While patients want to know the **cause** of their headache, this may not be possible. Both genetic and environmental factors contribute to processes that are not well understood.
- ▪ Patients may need to be persuaded that **tests are not helpful**.
- ▪ Patients with primary headache disorders may be advised that these tend to **remit with advancing age**.

Advice on further information that may be requested by patients is provided below under 2.5.2 *Advice to patients* (also, Additional file 5).

A series of patient information leaflets included here, in Section 4, provide basic explanations of migraine (also, Additional file 21), tension-type headache (also, Additional file 22), cluster headache (also, Additional file 23), medication-overuse headache (also, Additional file 24), trigeminal neuralgia (also, Additional file 26) and persistent idiopathic facial pain (also, Additional file 27), and their management.

##### Acknowledging and assessing impact

Assessment of impact **at start of treatment** establishes need and priority for treatment and measures the baseline for later evaluation of treatment. In addition to symptom-burden, impact of recurrent headache particularly includes disability.

The **HALT-90 Index** developed by *Lifting The Burden* is an easy-to-use instrument for assessing burden in terms of **lost productive time**. It is included here, among the management aids (3.4.2 *The Headache-Attributed Lost Time (HALT) Indices* (also, Additional file 18)).

In addition, recurrent disabling headache:

- ▪ may lead to **lifestyle compromise**, either in response to attacks or in a bid to avoid them (in this way, episodic headache can have continuous impact);
- ▪ has impact not only on the person with it but also on **other people** (family, work colleagues and employer).

##### Realistic aims of management

Primary headache disorders cannot be cured, but in most cases can be **effectively managed**. This means controlled by reductions in attack frequency and severity to minimise impact.

##### Causes and triggers

Many patients seek help in identifying triggers, but the importance of these should not be over-emphasised.

- ▪ Correctly identified triggers offer the possibility of **avoidance** (perhaps by life-style change) as a sometimes major contribution to management.
- ▪ When triggers are relevant to individual patients, they are usually **self-evident**.
- ▪ Triggers may be less readily identified when they are **cumulative** in their effect, jointly lowering the threshold above which attacks are initiated.
- ▪ Even when they are correctly identified, triggers are **not always avoidable**.

##### Follow-up

Every patient to whom treatment is offered, or whose treatment is changed, requires follow-up in order to ensure that optimum treatment has been established.

- ▪ The use of **outcome measures** is recommended to evaluate treatment and guide follow-up. The following are included here, among the management aids:
  - ▪ the **HURT questionnaire**, developed by *Lifting The Burden* expressly to guide management in primary care (see 3.5.2 *The Headache Under-Response to Treatment (HURT) questionnaire* (also, Additional file 20));
  - ▪ the **HALT-30 Index**, to record lost productive time in the preceding month (see 3.4.2 *The Headache-Attributed Lost Time (HALT) Indices* (also, Additional file 19));
  - ▪ a headache calendar (see below).
  - ▪ **Persistent management failure** is an indication for specialist referral.

##### Diaries and calendars

The principal distinction between these is in the amount of information collected. An example of each is provided here, among the management aids (see 3.3 *Headache diary and calendar to aid diagnosis and follow-up in primary care* (also, Additional file 16 and 17)).

**Diaries** capture more descriptive features of symptoms (headache intensity and character, associated symptoms), perhaps using free text.

- ▪ Diaries, used particularly as an aid to **diagnosis**, are useful for:
  - ▪ recording symptoms and temporal patterns that contribute to correct diagnosis;
  - ▪ recording acute medication use or overuse prior to diagnosis;
  - ▪ reporting lost productive time as part of pre-treatment assessment.

**Calendars** essentially note the temporal occurrence of headache episodes and related events such as menstruation and medication intake.

- ▪ Calendars, used in **follow-up**, are recommended in primary care for:
  - ▪ revealing associations with the menstrual cycle and possibly other triggers;
  - ▪ monitoring acute medication use or overuse during follow-up;
  - ▪ encouraging adherence to prophylactic medication;
  - ▪ recording treatment effect on headache frequency, and charting outcomes.

#### Advice to patients

This guide can be separately downloaded (Additional file 5).

Patients with headache disorders commonly request information. Many find or have found misleading information on the internet.

In addition to the advice below, **a series of patient information leaflets** developed by *Lifting The Burden* are provided here, in Section 4 and in the Additional files (see below).

- ▪ Four describe the important headache disorders (**migraine** (Additional file 21), **tension-type headache** (Additional file 22), **cluster headache** (Additional file 23) and **medication-overuse headache** (Additional file 24)), and their management.
- ▪ A fifth offers information on **female hormones and headache** (Additional file 25).
- ▪ Two further leaflets briefly describe **trigeminal neuralgia** (Additional file 26) and **persistent idiopathic facial pain** (Additional file 27).

##### Advice on non-drug treatments

Patients enquiring about the following may be given this summary advice.

- ▪ **Diets**. While healthy eating is always advisable, there is no reliable evidence that gluten-free, lactose-free, ketogenic or other specific diets prevent or improve headache disorders.
- ▪ **Biofeedback and relaxation therapies** can be helpful, and are potentially useful options when drug treatments must be avoided.
- ▪ **Cognitive behavioural therapy** may help patients develop coping strategies and better manage their symptoms. There is no good evidence to confirm benefit.
- ▪ **Physiotherapy** has proven benefits in some patients with tension-type headache. It requires skilled and individualised therapy, which is not widely available in many countries.
- ▪ **Aerobic exercise**. Limited data support the benefits of aerobic exercise on migraine and tension-type headache. Exercise has other important health benefits: improving physical strength, fitness and sleep, relieving depression and reducing blood pressure, cholesterol and weight.
- ▪ **Acupuncture** has differing forms, and is highly dependent on the skill of the therapist. There is **limited evidence** that acupuncture can be effective in reducing intensity and frequency of migraine attacks, but large clinical trials have failed to distinguish between acupuncture and sham procedures.
- ▪ **Devices**. Many are on the market, some very costly and promoted with insupportable claims of efficacy. “Testimonials” can be attributed to placebo effect and should be disregarded. The only clear recommendation possible is that successful trial usage should precede any expensive purchase.
  - ▪ A range of transcutaneous electrical nerve stimulators (TENS) and noninvasive neuromodulating devices for peripheral vagal nerve stimulation, supraorbital nerve stimulation and single-pulse transcranial magnetic stimulation are available, with evidence of efficacy in some people.
- ▪ **Herbals** are not recommended. Clinical trials data are limited and provide no evidence of safety in prolonged use. Herbals may interfere with other medications.
  - ▪ **Feverfew** preparations on sale everywhere are highly variable in content and their toxicity is not well understood.
  - ▪ **Butterbur** has some efficacy in migraine, but preparations on sale are variable in content and not all are free of liver toxins.
- ▪ **Nutraceuticals** are mostly not recommended. The following have some evidence for efficacy in migraine, and may be tried *where preparations of pharmaceutical quality are available*:
  - ▪ coenzyme Q10 (CoQ10) (100 mg three times daily);
  - ▪ magnesium (as citrate, starting at 100 mg three times daily to avoid diarrhoea, and increasing to 200 mg three times daily);
  - ▪ riboflavin (200 mg twice daily).
- ▪ **Homoeopathy** is of unproven value. There is no arguable case for over-the-counter sales of homoeopathic remedies.
- ▪ **Reflexology** has no scientific basis.
- ▪ **Cold packs or menthol gel** applied to the head and/or neck are found by some people to relieve pain or discomfort while being harmless and inexpensive.
- ▪ **Dental treatment**, including splints and bite-raising appliances, is of unproven value in treating headache and should be discouraged for this purpose.
- ▪ **Spectacles** should be professionally prescribed and worn when needed, but refractive errors are rarely a cause of troublesome headache.
  ▪ For the same reason, **accommodation training**, sometimes offered by optometrists, is not an accepted treatment for headache or likely to be beneficial.
- ▪ **Surgical procedures**. No surgical procedures produce benefit in migraine or tension-type headache. **Hysterectomy** has no place in migraine management.

##### Advice on hormonal contraception and HRT

With one important exception, migraine is not a contraindication to hormonal contraception or hormone replacement therapy (HRT).

- ▪ **Migraine with aura** and the **ethinylestradiol** component of combined hormonal contraceptives (CHCs) are **independent risk factors for stroke** in young women.
  - ▪ Every woman seeking hormonal contraception in primary care should be screened for migraine with aura and, if positive, offered **progestogen-only contraception** or non-hormonal alternatives.
- ▪ Otherwise, **headache is often a side-effect of CHCs** (pills, patches or vaginal rings), and many women report onset or aggravation of migraine after starting them.
  - ▪ Such symptoms usually resolve with continued use; if not, alternatives to CHCs should be offered.
- ▪ Other women, particularly those with menstrually-related migraine (without aura), report improvement, especially when CHCs are taken continuously without a week’s break.

The following **advice on hormonal contraception** may be given to patients with migraine:

- ▪ CHCs **increase risk of stroke** in young women with **migraine with aura**, who should therefore use alternatives;
- ▪ **a change** from migraine without aura to migraine with aura after starting CHCs is a clear signal to **stop immediately**;
- ▪ **progestogen-only contraception** is acceptable with any type or subtype of migraine.

The following **advice on hormone replacement therapy (HRT)** may be given to patients with migraine:

- ▪ **HRT is not contraindicated** in migraine with or without aura;
- ▪ decisions about commencing or continuing HRT should be made according to generally applicable criteria.

#### Management of migraine

This guide can be separately downloaded (Additional file 6).

Migraine is typically a **moderate-to-severe headache** accompanied by **nausea, vomiting and sensitivity to light and/or noise**. It is commonly disabling. It is usually episodic, but there is an uncommon chronic form.

##### Principles of management

- ▪ Good treatment of migraine begins with **education of patients**, explaining their disorder and the purpose and means of management.
- ▪ **Impact** of migraine should be assessed prior to planning treatment:
  - ▪ the **HALT-90 Index**, assessing burden in terms of lost productive time, is included here, among the management aids (see 3.4.2 *The Headache-Attributed Lost Time (HALT) Indices* (also, Additional file 18)).
- ▪ **Triggers and predisposing factors** should not be overemphasised but should nonetheless be considered early in management (with life-style modification when called for).
- ▪ Almost all patients with migraine will require **drug therapy for acute attacks**, but not necessarily prescription drugs (see 2.5.4 *Acute or symptomatic management of episodic migraine* (also, Additional file 7)).
- ▪ Any patient who is not well controlled with acute therapy alone and whose quality of life is impaired by migraine, whether adult or child, should be offered **prophylaxis** in addition (see 2.5.5 *Prophylactic management of episodic migraine* (also, Additional file 8)).
- ▪ Every patient to whom treatment is offered, or whose treatment is changed, requires **follow-up** to ensure that optimum treatment has been established.

##### Education of patients

A patient information leaflet on migraine and its management, developed by *Lifting The Burden*, is provided here in Section 4 (also, Additional file 21).

**Key points** of information are:

- ▪ migraine is a **common** disorder which, while it may be disabling, is **benign**;
- ▪ it is often **familial**, and probably genetically inherited;
- ▪ it cannot be cured but can be **successfully treated**;
- ▪ **trigger or predisposing factors** are common in migraine, and should be identified and avoided or modified when possible, but not all can be;
- ▪ a headache **calendar** helps good management by recording over time:
  - ▪ the symptoms and pattern of attacks (*eg*, menstrual relationship);
  - ▪ medication use (thus identifying overuse);
- ▪ **regular activity** (*eg*, sport or exercise 2–3 times per week) may reduce intensity and frequency of migraine attacks.

***Hormonal contraception and HRT***

Many women report onset or aggravation of migraine after starting combined hormonal contraceptives (CHCs). Others, particularly those with menstrually-related migraine, report improvement, especially when CHCs are taken continuously without a week’s break.

The following **advice on hormonal contraception** may be given:

- ▪ migraine with aura and the ethinylestradiol component of CHCs are **independent risk factors for stroke** in women, especially in those under 50 years;
- ▪ **alternatives to CHCs** are therefore very strongly recommended for women with migraine with aura;
- ▪ **a change** from migraine without aura to migraine with aura after starting CHCs is a clear signal to **stop immediately**;
- ▪ **progestogen-only contraception** is acceptable with any type or subtype of migraine.

The following **advice on hormone replacement therapy** (HRT) may be given:

- ▪ HRT is **not contraindicated** in migraine with or without aura;
- ▪ decisions about commencing or continuing HRT should be made according to generally applicable criteria.

A patient information leaflet on female hormones and headache, developed by *Lifting The Burden*, is provided here in Section 4 (also, Additional file 25).

##### Follow-up

- ▪ Use of a **calendar** is recommended to encourage adherence with prophylactic medication and record treatment effect. An example of a simple calendar is included here among the management aids (see 3.3 *Headache diary and calendar to aid diagnosis and follow-up in primary care* (also, Additional file 17)).
- ▪ The use of **outcome measures** is recommended to guide follow-up. The following are included here among the management aids:
  - ▪ the **HURT questionnaire** was developed expressly for primary care (see 3.5.2 *The Headache Under-Response to Treatment (HURT) questionnaire* (also, Additional file 20));
  - ▪ the **HALT-30 Index** records lost productive time during the preceding month (see 3.4.2 *The Headache-Attributed Lost Time (HALT) Indices* (also, Additional file 19)).
- ▪ **Persistent management failure** is an indication for specialist referral.

#### Acute or symptomatic management of episodic migraine

This guide can be separately downloaded (Additional file 7).

##### General principles

- ▪ All **adults** with episodic migraine should have access to acute medication.
- ▪ **Children** with short-lasting attacks may respond well to bed-rest without medical treatment.
- ▪ In adults and children, *regular* use of acute medication at high frequency (on >2 days/week) risks the development of **medication-overuse headache**.
- ▪ Many patients seek help in identifying **triggers** (see below). The importance of trigger factors in migraine is nonetheless often overemphasised.

##### Trigger and predisposing factors

- ▪ Correctly identified triggers offer the possibility of **avoidance** (perhaps by life-style change) as a sometimes major contribution to management.
- ▪ When triggers are relevant to individual patients, they are usually **self-evident**.
- ▪ **Cyclical hormonal fluctuations** may be an obvious factor in menstruating women.
- ▪ **Irregular lifestyle**, poor sleep pattern and “stress” are important predisposing factors in anybody with migraine. Missing meals is a potent trigger factor.
- ▪ Triggers may be less readily identified when they are **cumulative** in their effect, jointly lowering the threshold above which attacks are initiated.
- ▪ Even when they are correctly identified, triggers are **not always avoidable**.
- ▪ Contrary to popular belief, there is **no “migraine diet”**. The only dietary triggers with good evidential support are certain alcoholic drinks (especially red wine).

##### Drug intervention

**All patients** should climb a **treatment ladder** (stepped management), usually treating three attacks at each step before proceeding to the next. This strategy, when followed correctly, reliably achieves the most effective and cost-effective individualised care.

***Step one: symptomatic therapy***

- ▪ non-opioid analgesic
- ▪ plus, when needed, an antiemetic.

Recommended drugs and doses are shown in Table 7.

**Table 7:** Recommended drugs and doses for acute migraine therapy, step one

Analgesics	Antiemetics
Adults	
Non-steroidal anti-inflammatory drugs: • Acetylsalicylic acid 900–1000 mg *or* • Ibuprofen 400–800 mg *or* • Diclofenac 50–100 mg	• Domperidone 10 mg (supportive evidence of efficacy is for 20 mg, but the European Medicines Agency recommends restriction to 10 mg orally [up to three times daily] or 30 mg by suppository [up to twice daily]), *or* • Metoclopramide 10 mg (the European Medicines Agency restricts dosing to 10 mg [up to three times daily])
*Or* (where these are contraindicated): • Paracetamol 1000 mg^a^	
*Or* (possibly benefiting from the different mechanisms of action): • Combinations of paracetamol with acetylsalicylic acid or ibuprofen	
Children (when needed)	
Ibuprofen 200–400 mg according to age and weight	• Domperidone (dosage according to age and weight)

^a^Paracetamol on its own has lower efficacy and is **not** first-line treatment

*Drugs to avoid*

- ▪ **Opioids** (including codeine and dihydrocodeine) are ineffective for migraine, associated with multiple adverse effects, potentially addictive and commonly implicated in medication-overuse headache;
- ▪ **Barbiturates** have no place in the treatment of migraine.

*Principles of step one*

- ▪ Use **soluble analgesics** (or mouth-dispersible formulations with water) when available.
- ▪ Take **early** in the attack.
- ▪ Use **adequate dosage** (see Table 7: in most cases, adequate doses require more than a single tablet).
- ▪ A **prokinetic antiemetic counters gastric stasis**, an early feature of migraine, which impairs bioavailability of oral medication.
- ▪ Rectal formulations (where available) may be preferable in the presence of vomiting.
- ▪ Proceed to **step two after three attacks without success** (local guidelines may recommend trying more than one analgesic in step one before proceeding to step two).

***Step two: specific therapy***

- ▪ Where available, and unless contraindicated, specific therapy (Table 8) should be **offered to all patients failing step one**.
- ▪ Availability of drugs varies from country to country.

**Table 8:** Specific anti-migraine drugs, formulations and doses for step two (listed alphabetically)

Almotriptan	• Tablets 12.5 mg
Eletriptan	• Tablets 20 and 40 mg • Tablets 80 mg (not widely available) (for some people, 80 mg is effective when 40 mg is not)
Frovatriptan	• Tablets 2.5 mg
Naratriptan	• Tablets 2.5 mg
Rizatriptan	• Tablets and mouth-dispersible wafers 10 mg • Tablets 5 mg (to be used when propranolol is being taken concomitantly)
Sumatriptan	• Tablets and rapidly dissolving tablets 50 and 100 mg • Nasal spray 10 mg (licensed for adolescents) and 20 mg • Subcutaneous injection 6 mg
Zolmitriptan	• Tablets and mouth-dispersible tablets 2.5 and 5 mg • Nasal spray 5 mg

*Drugs to avoid*

- ▪ **Ergotamine** is a poor substitute for triptans: it has very low and unpredictable bioavailability, which impairs its efficacy, and poor tolerability. It is no longer recommended for routine use.

*Principles of step two*

- ▪ Triptans are more effective when taken while **headache is still mild** (but not during aura) (this instruction should be given only to patients who can reliably distinguish migraine from tension-type headache).
- ▪ The initial dose of all oral triptans (except eletriptan in some cases) is one tablet.
- ▪ A **second dose** for non-response is not recommended by most triptan manufacturers but, taken not less than 2 h after the first, may nonetheless be effective in some cases.
- ▪ Triptans should **not be used regularly on ≥10 days/month** to avoid the risk of medication-overuse headache.
- ▪ Triptans differ slightly, but there are large and unpredictable individual **variations in responses** to them:
  - ▪ one may work where another has not;
  - ▪ patients are best served if they can try several, in different formulations, and choose between them.
- ▪ When **nausea** is present, domperidone 10 mg may be added.
- ▪ When **vomiting** is present, zolmitriptan nasal spray (absorbed through the nasal mucosa) or sumatriptan subcutaneous injection may be preferred.
- ▪ Efficacy of sumatriptan may be increased by combination with naproxen 500–1000 mg (there are no data on combinations of other triptans and NSAIDs).
- ▪ When all other triptans are ineffective, sumatriptan by subcutaneous injection 6 mg should be considered.
- ▪ Triptans are associated with return of symptoms within 48 h (**relapse**) in up to 40% of patients who have initially responded (see below).

*Treatment of relapse*

- ▪ A **repeat dose** of a triptan is usually effective.
- ▪ A further relapse may occur:
  - ▪ in a minority of patients, this happens **repeatedly**, a major management problem with high risk of developing **medication-overuse headache**;
  - ▪ a different triptan should be tried in future attacks;
  - ▪ concomitant use of a triptan and naproxen may reduce susceptibility to relapse.

*Contraindications and special precautions in step two*

- ▪ Triptans should not be taken **during aura** of migraine with aura, but at the onset of headache.
- ▪ All triptans should be **avoided** by people with:
  - ▪ uncontrolled hypertension (one reason for measuring blood pressure);
  - ▪ coronary heart disease, cerebrovascular disease or peripheral vascular disease;
  - ▪ multiple risk factors for coronary or cerebrovascular disease;
- ▪ In the **elderly**, all of these are more common, and triptans should therefore be used with **greater caution**.
- ▪ In **pregnancy**: limited safety data are available only for sumatriptan, which should be used with caution and **only under specialist supervision**.
- ▪ In addition, there are **specific precautions** attached to some triptans (see pharmacopoeia).

*Step two for children and adolescents*

- ▪ Failure of step one in **children** is an indication for specialist referral.
  - ▪ No specific anti-migraine drug has been shown to have efficacy in children (under 12 years old).
- ▪ **For adolescents** (12–17 years), the following have efficacy and are approved:
  - ▪ sumatriptan nasal spray 10 mg;
  - ▪ zolmitriptan nasal spray 2.5 mg and/or 5 mg (in some countries).

##### Follow-up

Every patient to whom treatment is offered, or whose treatment is changed, requires follow-up to ensure that optimum treatment has been established.

- ▪ Use of a **calendar** is recommended to monitor acute medication use or overuse. An example of a simple calendar is included here among the management aids (see 3.3 *Headache diary and calendar to aid diagnosis and follow-up in primary care* (also, Additional file 17)).
- ▪ The use of **outcome measures** is recommended to guide follow-up. The following are included here among the management aids:
  - ▪ the **HURT questionnaire** was developed expressly for primary care (see 3.5.2 *The Headache Under-Response to Treatment (HURT) questionnaire* (also, Additional file 20));
  - ▪ the **HALT-30 Index** records lost productive time during the preceding month (see 3.4.2 *The Headache-Attributed Lost Time (HALT) Indices* (also, Additional file 19)).
- ▪ **Failure of acute therapy** may be an indication for prophylaxis (see below).

#### Prophylactic management of episodic migraine

This guide can be separately downloaded (Additional file 8).

##### General principle

Any patient with migraine who is **not well controlled with acute therapy alone**, whether adult or child, should be offered prophylaxis **in addition to** acute medication.

##### Indications for prophylaxis

Prophylactic therapy should be **added** when migraine **impairs quality of life**, *and*

- ▪ attacks cause disability on **two or more days per month**, *and*
- ▪ **acute therapy has been optimised** but does not prevent this, or is poorly tolerated, *or*
- ▪ there is a risk of **over-frequent use of acute therapy**, even when it is effective; *and*
- ▪ the **patient is willing** to take daily medication.

**Frequent absences from school** because of migraine are an additional indication for prophylaxis in **children** (who should be referred for specialist assessment).

##### Principles of prophylaxis

- ▪ A **calendar** should be kept by every patient on prophylaxis to assess efficacy and promote adherence. An example of a simple calendar is included here among the management aids (see 3.3 *Headache diary and calendar to aid diagnosis and follow-up in primary care* (also, Additional file 17)).
- ▪ **Poor adherence** is a major factor impairing efficacy of migraine prophylactics; once-daily dosing is associated with better adherence.
- ▪ The **dose** of any drug **should start low** in the suggested range and be increased in the absence of troublesome side-effects.
- ▪ Drugs that appear ineffective should **not be discontinued too soon**; 2–3 months may be the minimum to achieve and observe efficacy.
- ▪ Failure of one drug **does not predict failure of others** in a different class.
- ▪ **Tapered withdrawal** may be considered after 6 months of good control, and should be considered no later than after 1 year.
- ▪ **Children** requiring prophylactic medication should be referred for specialist assessment.

##### Effective drugs for prophylaxis

A range of drugs have proven efficacy (Table 9), all with contraindications and side-effects (refer to pharmacopoeia).

- ▪ Availability and regulatory approval vary from country to country, and many are not specifically licensed for migraine prophylaxis. Use of drugs off-licence rests on individual clinical responsibility.
- ▪ Across the range, expected benefit is no greater than 50% fewer attacks in 50% of users after 3 months of treatment (with individual benefit varying between zero and [rarely] 100%).
- ▪ Once daily dosing (as opposed to more frequent) is associated with better adherence, an important determinant of efficacy.

**Table 9:** Migraine prophylactic drugs with evidence of efficacy in adults (drugs are listed in a suggested order of use; within classes [beta blockers and CGRP monoclonal antibodies], they are listed alphabetically)

Beta-adrenergic blockers without partial agonism: • atenolol 25-100 mg twice daily • bisoprolol 5-10 mg once daily • metoprolol 50-100 mg twice daily or modified-release 200 mg once daily • propranolol LA 80-160 mg once to twice daily	• observe general contraindications, including comorbid depression • propranolol has best evidence of efficacy, but not evidence of best efficacy • cardioselective and non-lipophyllic drugs (bisoprolol, atenolol, metoprolol) are likely to be better tolerated
Amitriptyline 10-100 mg at night	• may be preferred when migraine coexists with tension-type headache, depression or sleep disturbance
Topiramate 50 mg twice daily	• titrate over 4 weeks from 25 mg once daily • contraindicated in pregnancy
Candesartan 16 mg once daily	• start at 8 mg once daily and titrate weekly • contraindicated in pregnancy
Sodium valproate 600-1500 mg daily	• titrate upwards • **avoid altogether in women of child-bearing potential** (even on contraception); absolutely contraindicated in pregnancy
Flunarizine 5-10 mg once daily	• observe general contraindications, including comorbid depression
CGRP monoclonal antibodies (to the peptide or its receptor): • erenumab 70 or 140 mg s/c once monthly • fremanezumab 225 mg s/c once monthly or 675 mg s/c once quarterly • galcanezumab 240 mg s/c, then 120 mg s/c once monthly	• newly licensed, not yet universally available or reimbursed, usually restricted to specialist care and reserved for those failing (or not tolerating) other prophylactics • all self-administered by auto-injector • high relative cost

##### Other treatments patients may ask about

- ▪ **Onabotulinum toxin A (Botox)**. This is **not effective** in episodic migraine and is not recommended for this condition.
- ▪ **Surgical procedures**. There is **no evidence** to support any surgical procedure as a treatment for episodic migraine.
  - ▪ In particular, migraine is not improved by closure of patent foramen ovale (PFO). This procedure should not be undertaken for migraine prophylaxis: it carries a small but relevant risk of serious adverse events including stroke, pericardial tamponade, atrial fibrillation and death.
- ▪ **Acupuncture** has differing forms, and is highly dependent on the skill of the therapist. There is **limited evidence** that acupuncture can be effective in reducing intensity and frequency of migraine attacks, but large clinical trials have failed to distinguish between acupuncture and sham procedures. Benefits experienced by some patients may be attributable to placebo effect.
- ▪ **Devices**. Many are on the market, some very costly and promoted with insupportable claims of efficacy. “Testimonials” can be attributed to placebo effect and should be disregarded. The only clear recommendation possible is that successful trial usage should precede any expensive purchase.
  - ▪ A range of transcutaneous electrical nerve stimulators (TENS) and noninvasive neuromodulating devices for peripheral vagal nerve, supraorbital nerve and single-pulse transcranial magnetic stimulation are available, with evidence of efficacy in some people.
- ▪ **Herbals** are **not recommended**. Evidence of both efficacy and safety in prolonged use is poor. They may interfere with other medications.
  - ▪ **Feverfew** preparations are highly variable in content, and not all of pharmaceutical quality. Their toxicity is not well understood.
  - ▪ **Butterbur** has some efficacy and is approved for use in some countries, but preparations on sale are variable in content and not all of pharmaceutical quality (not guaranteed to be free of liver toxins).
- ▪ **Nutraceuticals** are mostly not recommended. The following have some evidence for efficacy, and may be tried where preparations of pharmaceutical quality are available:
  - ▪ **coenzyme Q10** (CoQ10) (100 mg three times daily);
  - ▪ **magnesium** (as citrate, starting at 100 mg three times daily to avoid diarrhoea, and increasing to 200 mg three times daily);
  - ▪ **riboflavin** (200 mg twice daily).
- ▪ **Homoeopathy** is of unproven value. There is no arguable case for over-the-counter sales of homoeopathic remedies.

##### Prophylaxis in pregnancy

- ▪ This is **better avoided**, and rarely required since migraine often remits during pregnancy.
- ▪ Sodium valproate is **absolutely contraindicated**; topiramate and candesartan are **contraindicated**.
- ▪ **Propranolol and amitriptyline** have best evidence of safety, but specialist guidance is recommended.
- ▪ Riboflavin (vitamin B2), 200 mg twice daily, may be tried, but may not show efficacy for 3 months.

##### Follow-up

Every patient to whom prophylactic treatment is offered, or whose treatment is changed, requires follow-up to ensure that optimum treatment has been established.

- ▪ Use of a **calendar** is recommended to encourage adherence with prophylactic medication and record treatment effect. An example of a simple calendar is included here among the management aids (see 3.3 *Headache diary and calendar to aid diagnosis and follow-up in primary care* (also, Additional file 17)).
- ▪ The use of **outcome measures** is recommended to guide follow-up. The following are included here among the management aids:
  - ▪ the **HURT questionnaire** was developed expressly for primary care (see 3.5.2 *The Headache Under-Response to Treatment (HURT) questionnaire* (also, Additional file 20));
  - ▪ the **HALT-30 Index** records lost productive time during the preceding month (see 3.4.2 *The Headache-Attributed Lost Time (HALT) Indices* (also, Additional file 19))

##### When prophylaxis fails

- ▪ Failure may be due to subtherapeutic dosage (itself perhaps due to non-adherence) or insufficient duration of treatment.
- ▪ The following actions are recommended:
  - ▪ review the **diagnosis**;
  - ▪ review **adherence**;
  - ▪ review **other medication**, especially for **overuse**.
- ▪ When prophylaxis still fails to have clear benefit, **discontinue** it.
- ▪ When all options fail, **specialist referral** is indicated.

#### Management of chronic migraine

This guide can be separately downloaded (Additional file 9).

Chronic migraine develops in a small minority of people with episodic migraine. It is one of the syndromes characterised by **headache on ≥15 days/month**, but is **not simply migraine that is more frequent**: it is often complicated by medication overuse, depression, anxiety and low back and/or neck pain.

**Chronic migraine** should be:

- ▪ **suspected** in any patient:
  - ▪ with a history of migraine
  - ▪ who reports (or records in a diary) **headache on ≥15 days/month**;
- ▪ **diagnosed**, in the absence of medication overuse, in patients with:
  - ▪ **headache on ≥15 days/month** over the last 3 months, which
  - ▪ on **≥8 days/month**:
    - ▪ fulfilled the diagnostic criteria for migraine, *or*
    - ▪ responded to migraine-specific drug treatment.

The **presence of medication overuse** in such patients complicates the diagnosis:

- ▪ medication-overuse headache (MOH) is another syndrome characterised by headache on ≥15 days/month;
- ▪ chronic migraine and MOH are **not mutually exclusive** but, even when the conditions above are met, only MOH and not chronic migraine may be present when medication is being overused;
- ▪ medication overuse, whether or not occurring with chronic migraine, must **always be recognised and managed as a separate medical problem**.

Medication-overuse, and MOH, can often be successfully managed in primary care (see 2.5.9 *Management of medication-overuse headache* (also, Additional file 12)), but patients with **chronic migraine** should be **referred for specialist care**.

##### Principles of management

Chronic migraine is **difficult to treat**. Management in specialist care includes:

- ▪ **education** of patients about chronicity and its causes and risk factors;
- ▪ recognition and management of **medication overuse**, when present;
- ▪ management of any **comorbidities**;
- ▪ use of **preventative drugs** (Table 10);
- ▪ **follow up**, with both medical and psychological care.

**Table 10:** Drugs used by specialists in chronic migraine prophylaxis

Topiramate, 50 mg or more twice daily	
Onabotulinum toxin A, 155-195 units by multisite injection	• not licensed for chronic migraine in some countries, *or* • not reimbursed, *and/or* • regulators require prior failure of two or more of the drugs used in prophylaxis of episodic migraine
CGRP monoclonal antibodies (to the peptide or its receptor): • erenumab 70 or 140 mg s/c once monthly • fremanezumab 225 mg s/c once monthly or 675 mg s/c once quarterly • galcanezumab 240 mg s/c, then 120 mg s/c once monthly	• newly licensed, not yet universally available or reimbursed, usually restricted to specialist care and reserved for those failing (or not tolerating) other prophylactics • all self-administered by auto-injector • high relative cost

##### Preventative drugs

Those used in specialist care, with evidence of efficacy, are shown in Table 10.

#### Management of tension-type headache (TTH)

This guide can be separately downloaded (Additional file 10).

Tension-type headache (TTH) is typically a **mild-to-moderate headache** of highly variable frequency and duration, **without associated symptoms** or the specific features of migraine.

**Two types** of TTH are medically important:

- ▪ **frequent episodic TTH**, with headache attacks on 1–14 days/month on average;
- ▪ **chronic TTH**, one of the syndromes characterised by headache occurring on ≥15 days/month, either with highly-frequent attacks or, occasionally, continuous and unremitting.

##### General principles

- ▪ Good treatment of patients with troublesome TTH (of either type) begins with their **education**, explaining their disorder and the purpose and means of management.
- ▪ **Impact** of TTH should be assessed prior to planning treatment:
  - ▪ the **HALT-90 Index**, assessing burden in terms of lost productive time, is included here, among the management aids (see 3.4.2 *The Headache-Attributed Lost Time (HALT) Indices* (also, Additional file 18)).
- ▪ **Infrequent** headaches (on ≤2 days/week) are managed with over-the counter (OTC) analgesics.
- ▪ When headache is **more frequent**:
  - ▪ advice on **lifestyle** may be helpful, possibly accompanied by psychological intervention such as cognitive behavioural therapy;
  - ▪ analgesics (even OTC) should be used with care because of the risk of **medication-overuse headache**;
  - ▪ **prophylaxis** may be indicated.

##### Education of patients

A patient information leaflet on TTH and its management, developed by *Lifting The Burden*, is provided here in Section 4 (also, Additional file 22).

**Key points** of information are:

- ▪ TTH is a **very common** disorder but, while it may be disabling and troublesome when headaches are frequent, it is **benign**;
- ▪ **episodic TTH** can be successfully treated, usually with OTC analgesics;
- ▪ **over-frequent use** of medications, even OTC, will make headaches worse;
- ▪ **chronic TTH** cannot be regularly treated with analgesics and usually requires other long-term continuous medication and/or non-pharmacological interventions;
- ▪ a headache **calendar** helps good management by recording over time the symptoms and pattern of attacks and medication use;
- ▪ **predisposing factors** sometimes include stress and/or poor head and neck posture;
- ▪ **regular activity** (*eg*, sport or exercise 2–3 times per week) may help frequent TTH.

##### Acute intervention

Symptomatic treatment with OTC analgesics (Table 11) is appropriate for episodic TTH occurring on **≤2 days/week**.

**Table 11:** Analgesics for episodic tension-type headache

Ibuprofen 400–800 mg	• For adults, and • Drug of choice for children (200–400 mg according to age and weight)
Acetylsalicylic acid 600–1000 mg	• Adults only
Either of these in combination with paracetamol 1000 mg	• Formal evidence is lacking, but the different mechanisms of action may enhance effect
Any of these in combination with caffeine	• Commonly included in analgesic combination-medications
Paracetamol 1000 mg	• On its own has lower efficacy • Therefore reserved for those in whom NSAIDS are contraindicated

***Drugs to avoid***

- ▪ **Opioids** (including codeine and dihydrocodeine) are ineffective for headache, associated with multiple adverse effects, potentially addictive and commonly implicated in medication-overuse headache.
- ▪ **Barbiturates** have no place in the treatment of TTH.
- ▪ **Metamizol** has limited evidence for efficacy and is associated with agranulocytosis.
- ▪ **Triptans** are specific for migraine, and ineffective in TTH.

***Principles of acute intervention***

- ▪ Episodic TTH occurring on **≤2 days/week** can usually be successfully treated with **OTC analgesics alone**;
- ▪ As the **frequency of headaches increases**, so does the risk of medication overuse:
  - ▪ episodic TTH on **>2 days/week** is a clear indication for **prophylaxis** (see below) in place of, rather than in addition to, acute intervention;
  - ▪ acute treatments are **unlikely to be effective in chronic TTH** and put the patient at clear risk of medication-overuse headache.

##### Prophylaxis

***Principles of prophylaxis***

- ▪ A **calendar** should be kept to assess efficacy and promote adherence. An example of a simple calendar is included here among the management aids (see 3.3 *Headache diary and calendar to aid diagnosis and follow-up in primary care* (also, Additional file 17)).
- ▪ Patients receiving medication more often used as an antidepressant should be **advised of this, and why**; otherwise, they may default when they find out.
- ▪ Prophylaxis that appears **ineffective** should not be discontinued too soon; 2–3 months may be the minimum to achieve and observe efficacy.
- ▪ **Tapered withdrawal** may be considered after 6 months of good control, but prolonged treatment is sometimes indicated.

***Effective drugs***

A narrow range of drugs have efficacy (Table 12), although none is specifically licensed for TTH prophylaxis. Use of drugs off-licence rests on individual clinical responsibility.

**Table 12:** Prophylactic drugs with some evidence of efficacy in frequent episodic or chronic tension-type headache

Amitriptyline, 10–100 mg at night	• Drug of choice for frequent episodic or chronic TTH; • Intolerance is reduced by starting at a low dose (10 mg) and incrementing by 10–25 mg each 1–2 weeks
Nortriptyline (replacing amitriptyline at the same dose)	• Fewer anticholinergic side-effects but less good evidence of efficacy
Mirtazapine, 15–30 mg once daily	• Second-line option
Venlafaxine, 75–150 mg once daily	• Third-line option

***Drugs to avoid***

- ▪ **Onabotulinum toxin A** is ineffective in TTH.

***Non-pharmacological prophylaxis***

- ▪ There is limited evidence that **acupuncture** is effective in reducing intensity and frequency of TTH episodes. While some patients experience benefit, this may be due to placebo effect. Acupuncture has differing forms, and is highly dependent on the skill of the therapist.
- ▪ There is well-documented evidence of efficacy of various forms of **biofeedback**. They are highly dependent on the skill of the therapist.

##### Follow-up

Every patient to whom treatment is offered, or whose treatment is changed, requires follow-up to ensure that optimum treatment has been established.

- ▪ Use of a **calendar** is recommended to monitor acute medication use or overuse, or to encourage adherence to prophylactic medication, and to record treatment effect. An example of a simple calendar is included here among the management aids (see 3.3 *Headache diary and calendar to aid diagnosis and follow-up in primary care* (also, Additional file 17)).
- ▪ The use of **outcome measures** is recommended to guide follow-up. The following are included here among the management aids:
  - ▪ the **HURT questionnaire** was developed expressly for primary care (see 3.5.2 *The Headache Under-Response to Treatment (HURT) questionnaire* (also, Additional file 20));
  - ▪ the **HALT-30 Index** records lost productive time during the preceding month (see 3.4.2 *The Headache-Attributed Lost Time (HALT) Indices* (also, Additional file 19)).

***When prophylaxis fails***

- ▪ Failure may be due to subtherapeutic dosage (itself perhaps due to non-adherence) or insufficient duration of treatment.
- ▪ The following actions are recommended:
  - ▪ review the **diagnosis**;
  - ▪ review **adherence**;
  - ▪ review **other medication**, especially for **overuse**;
- ▪ When prophylaxis still fails to have clear benefit, **discontinue** it.
- ▪ When all options fail, **specialist referral** is indicated.

##### Pain management

- ▪ Despite best efforts, **chronic TTH is often refractory** to medical treatment or may become so.
- ▪ Patients in this situation require referral into a pain management programme with emphasis on **psychological approaches**.

#### Management of cluster headache

This guide can be separately downloaded (Additional file 11).

Cluster headache, a type of trigeminal autonomic cephalalgia, is characterised by frequently recurring, localised, short-lasting but **extremely severe headache**, which is accompanied by a set of highly characteristic **autonomic symptoms**.

- ▪ Cluster headache is **easily recognisable** (see 2.4.2 *Typical features of the headache disorders relevant to primary care* (also, Additional file 2)).
- ▪ It should **never be missed**.

It has **two subtypes**:

- ▪ **episodic cluster headache**, with attacks occurring in bouts (clusters) that last for a few or many weeks and then remit for ≥3 months;
- ▪ **chronic cluster headache**, less common, but persisting without remissions, or with remissions of <3 months.

##### General principles

- ▪ Patients with this disorder **suffer very badly** if ineffectively treated:
  - ▪ cluster headache management is, at least initially, **better left to specialists** who see this disorder frequently;
  - ▪ on first presentation it demands **accelerated referral** for investigation and treatment;
  - ▪ **recognition** in primary care is crucial to ensure prompt referral.
- ▪ The **objective** of management in both episodic and chronic subtypes is **total attack suppression**. This is not always achievable.
- ▪ Both acute medication and prophylaxis have a role in management, but **preventative drugs are the mainstay of treatment** in most cases.
- ▪ Once effective treatment has been established, future clusters, or maintenance therapy in the case of chronic cluster headache, may be managed in primary care.

##### Acute therapies

There are limited options (Table 13), but efficacy may be high.

- ▪ Availability varies between countries.
- ▪ Most are not specifically licensed for cluster headache. Use of drugs off-licence rests on individual clinical responsibility.

**Table 13:** Acute therapies used in cluster headache by specialists

Triptans:	None can be recommended for use more than twice a day
• Sumatriptan 6 mg s/c	• The most highly-effective acute treatment
• Zolmitriptan 5 mg nasal spray	• Less-certain efficacy but an alternative for those unable or unwilling to use sumatriptan s/c
• Sumatriptan 20 mg nasal spray	• Less-certain efficacy: absorption depends largely on ingestion
Oxygen 100% at ≥12 l/min until response, or for ≥15 min	• Requires a non-rebreathing mask and regulator; • Helps some people and may be used as frequently as needed

***Drugs to avoid***

- ▪ **Oral triptans** are slow in onset of action and are not useful substitutes.
- ▪ **Analgesics**, including opioids, have little or no place in treating cluster headache.

##### Preventative therapy

Specialists employ the following:

- ▪ **transition therapy** (Table 14), used at onset of treatment to achieve more rapid response during dose escalation of any of the preventative drugs;
- ▪ **maintenance prophylaxis** (Table 15), balancing efficacy of drugs against their significant toxicity (refer to pharmacopoeia).

**Table 14:** Transition therapies used in cluster headache by specialists

Prednisolone 60–80 mg once daily	• For 2–4 days, discontinued by dose reduction over 1–3 weeks
Greater occipital nerve blockade	• Using various agents

**Table 15:** Drugs used by specialists in maintenance prophylaxis of cluster headache

Verapamil 240–960 mg daily	• ECG monitoring advised
Lithium carbonate 600–1600 mg daily	• Serum levels must be regularly monitored
Topiramate 50–100 mg twice daily	• Less evidence of efficacy, but no monitoring required

***Principles of preventative therapy***

- ▪ **Prophylaxis** of episodic cluster headache should begin **as early as possible** after the start of a new cluster bout.
- ▪ **Failure** of one drug does not predict failure of others.
- ▪ **Combinations** of drugs may be tried, but the potential for toxicity is obviously high.
- ▪ For episodic cluster headache, maintenance prophylaxis should be **discontinued** by tapering, usually 2 weeks after full remission.
- ▪ For chronic cluster headache, maintenance prophylaxis may need to be continued **long-term**.

***Other treatment options***

- ▪ **Neuromodulation**, non-invasive or invasive, is occasionally used by specialists.

##### Follow-up

Every patient with active cluster headache requires **frequent follow-up** both to ensure that optimum acute and preventative treatments are maintained and to **monitor for treatment toxicity**.

- ▪ Patients with episodic cluster headache in remission should be advised to **return promptly** at the onset of the next cluster episode.

##### Information for patients

A patient information leaflet on cluster headache and its management, developed by *Lifting The Burden*, is provided here in Section 4 (also, Additional file 23).

#### Management of medication-overuse headache (MOH)

This guide can be separately downloaded (Additional file 12).

Medication-overuse headache (MOH) is one of the syndromes characterised by **headache occurring on ≥15 days/month**. It is often daily, but variable in site, intensity and character. It greatly impairs quality of life.

MOH is an aggravation of a prior headache disorder (usually migraine, but sometimes tension-type headache) caused by chronic overuse of medication taken to treat it.

##### General principles

- ▪ **Prevention**, through **education**, is preferable to cure.
- ▪ Once MOH has developed, **early intervention** has better chance of success.
- ▪ The **necessary management** of established MOH is to **stop overuse** of the suspected medication(s).
- ▪ **Patient education**, that medication taken to relieve headache is in fact its cause, is the essential first step:
  - ▪ success in management depends crucially on **patients’ understanding** that their medication taken to relieve their headache is in fact its cause.
- ▪ Management is usually possible in **primary care**.
- ▪ The **long-term prognosis is usually very good**. Most cases revert to episodic headache, although the outcome depends on:
  - ▪ the type of headache from which MOH developed;
  - ▪ the class of medication overused (opioids causing greatest difficulty);
  - ▪ the **duration of overuse**;
  - ▪ **comorbidities** (psychiatric, or other causes of chronic pain).

##### Education of patients

A patient information leaflet on medication-overuse headache and its management, developed by *Lifting The Burden*, is provided here in Section 4 (also, Additional file 24).

**Key points** of information are:

- ▪ The “treatment” a patient is taking for headache is actually **the cause** of it.
- ▪ Effective treatment requires, in the first instance, **stopping use of the suspected medication(s)** (withdrawal):
  - ▪ there is **no other option**;
  - ▪ many patients recover from this alone.
- ▪ **Initial worsening** of symptoms for 1–2 weeks during and after withdrawal must be expected.
- ▪ The **outcome is usually very good**, with reversion in most cases, within 2 months, to the antecedent episodic headache disorder.

##### Objectives

There are **four separate objectives** in the complete management of MOH, and all are important:

- ▪ **stop** the overused medication;
- ▪ **recovery** from MOH (which should follow);
- ▪ **review** and reassess the underlying headache disorder (usually migraine or tension-type headache);
- ▪ **prevent relapse**, while allowing acceptable use of medications.

In addition, **comorbidities** may require management.

##### Principles of withdrawal

- ▪ **Worsening headache** for 1–2 weeks is almost inevitable:
  - ▪ accordingly, withdrawal should be **planned** to avoid unnecessary lifestyle disruption;
  - ▪ 1–2 weeks’ **sick leave** may be needed;
  - ▪ **admission to hospital** during withdrawal is **rarely necessary** unless:
    - ▪ overused medication(s) include opioids;
    - ▪ for management of comorbidities.
- ▪ **Withdrawal** may be undertaken in **any of three ways**, the choice being made by the patient:
  - ▪ **abruptly**:
    - ▪ there is evidence that this is the most successful approach;
  - ▪ by **tapering** over a period of 2–4 weeks:
    - ▪ withdrawal symptoms are likely to be less intense but more prolonged;
  - ▪ by **replacing** the overused medication(s) with **naproxen 500 mg twice daily** for 3–4 weeks and no longer:
    - ▪ the purpose is to break the behavioural “have headache – take medication” link;
    - ▪ many patients become headache-free on this medication;
    - ▪ naproxen must be stopped after this period (never continued).
- ▪ Headache usually shows signs of **improvement** 1–2 weeks after stopping overused medication(s).
- ▪ **Recovery** continues slowly for up to 2 months.
- ▪ **Prophylaxis** against the antecedent headache (most often migraine) may be introduced on its return, or commenced in parallel with the withdrawal process.

##### Follow-up

Every patient stopping medication overuse requires follow-up in order to provide support and observe outcome.

- ▪ **First review** is advised **after 2–3 weeks** to ensure withdrawal has been successfully achieved.
- ▪ Use of a **calendar** during withdrawal is strongly recommended to record symptoms and medication use, and to record changing headache pattern. An example of a simple calendar is included here among the management aids (see 3.3 *Headache diary and calendar to aid diagnosis and follow-up in primary care* (also, Additional file 17)).
- ▪ Most patients revert to their **antecedent headache** (usually migraine or tension-type headache) within 2 months; this will need review and appropriate management.
- ▪ The **relapse** rate is high within the first year: further follow-up is important to avoid it, and many patients require extended support.

##### Re-introducing withdrawn medication

- ▪ Previously overused medications should be reassessed:
  - ▪ **alternatives** should be used whenever possible;
  - ▪ if still needed, they may be **cautiously reintroduced** after 2 months.
- ▪ Frequency of use should be on **no more than 10 days/month**:
  - ▪ use on more than 6 days/month raises the risk of recidivism;
  - ▪ patients should avoid treating headaches on more than 3 days in a row.

#### Management of trigeminal neuralgia and persistent idiopathic facial pain

This guide can be separately downloaded (Additional file 13).

Management of these uncommon but troublesome disorders is **better left to specialists**.

- ▪ Recognition in primary care is crucial to ensure prompt referral.

##### Trigeminal neuralgia (TN)

This disorder presents as recurrent, unilateral, **brief but severe, electric-shock-like pains** in the distribution of the trigeminal nerve, abrupt in onset and termination and often triggered by innocuous stimuli.

It is not common, affecting 1–2 in every 1000 people. Women are twice as likely to be affected as men.

***Principles of management***

- ▪ TN is **extremely painful**, and untreated is physically, psychologically and socially debilitating:
  - ▪ patients may avoid the triggers of eating and drinking, **seriously impairing food and fluid intake**.
  - ▪ TN therefore demands **accelerated specialist referral** for investigation and treatment.
- ▪ Good treatment begins with **education of patients**, explaining their disorder and the purpose and means of management.
- ▪ The **objective** in management, by medical or surgical means, is abatement of attacks and pain freedom. This is not always achievable.
- ▪ **MRI is mandatory** since classical TN and secondary TN (due usually to cerebellopontine angle tumour, AV-malformation or multiple sclerosis) may be indistinguishable by symptom presentation.
- ▪ **First-line** treatment is prophylactic (antiepileptic) medication.
- ▪ Acute therapies (opioids or other analgesics) have **no place** in management since attacks are very short-lasting.
- ▪ Severe exacerbations with anorexia and dehydration, due to pain triggered by eating or drinking, may require **hospital admission** for intravenous hydration and medication.

***Education of patients***

A patient information leaflet on trigeminal neuralgia is provided here in Section 4 (also, Additional file 26).

**Key points** of information are:

- ▪ TN produces very characteristic, very severe, electric-shock-like pains:
  - ▪ along a nerve on one side of the face, usually in the **cheek or jaw**;
  - ▪ repetitively, in short-lasting bouts (up to 2 min), which:
    - ▪ occur **daily for weeks or months** but sometimes remit spontaneously;
    - ▪ usually **start without warning**, but can be **provoked** by light touch, wind, cold air, eating, drinking, brushing the teeth or speaking.
- ▪ The **cause** of TN is often not known:
  - ▪ some people have a blood vessel in close contact with and compressing the affected nerve: an **MRI brain scan** is required to show this;
  - ▪ however, there are other unknown causes.
- ▪ **Specialist referral** is therefore necessary.
- ▪ There are a **number of treatments** for TN, which often work well:
  - ▪ these are **preventative** medications, to be taken daily;
  - ▪ **painkillers do not help**;
  - ▪ occasionally, surgery is required, but as a last resort;
  - ▪ TN does **not require dental treatment**.

***Preventative medications***

A narrow range of antiepileptic drugs are effective, and used by specialists (Table 16). **Maximum dosages** may be necessary to achieve pain relief, and balancing efficacy against toxicity is difficult.

**Table 16:** Drugs used by specialists in trigeminal neuralgia prophylaxis

First line: • Carbamazepine 200–2400 mg daily • Oxcarbazepine 600–2400 mg daily	These drugs: • reduce efficacy of oral contraceptives; • may induce hyponatraemia (especially oxcarbazepine): regular monitoring is advised; • Mmay induce osteoporosis in long-term treatment: prophylaxis against this is advised
Second-line (either as monotherapy or as add-on medication): • Gabapentin 600–3600 mg daily • Pregabalin 150–600 mg daily • Lamotrigine 200–1000 mg daily (very slow up-titration necessary)	

***Principles of drug prophylaxis***

- ▪ Dosages should be **up-titrated slowly** until pain relief is achieved or side effects become unacceptable.
- ▪ Patients established on medication may be taught to **titrate up and down**, according to symptom severity.
- ▪ **Combinations** may cause fewer side-effects because lower doses may be required of each drug.
- ▪ Treatment may be **slowly tapered** after complete freedom from pain, and discontinued in the absence of relapse.

***Other treatment options in medically refractory patients***

- ▪ **Neurosurgical treatments** are relevant when medical treatment with maximum tolerated doses achieve insufficient efficacy, but:
  - ▪ microvascular decompression (appropriate when neurovascular compression, not merely contact, has been demonstrated) carries a small risk of severe complications such as cranial nerve palsy or stroke;
  - ▪ gamma-knife and/or percutaneous procedures (balloon compression, glycerol injection, thermocoagulation or pulsed radiofrequency treatment) targeting the trigeminal ganglion are less invasive but probably less efficacious.

***Follow-up***

While every patient with TN requires specialist initial management, long-term follow-up once stable is appropriate in primary care.

- ▪ Patients should be educated on:
  - ▪ how to **taper medication** cautiously once pain freedom is achieved;
  - ▪ how to **reintroduce medication** by careful up-titration if/when pain returns.

##### Persistent idiopathic facial pain (PIFP)

Previously termed “atypical facial pain”, this disorder presents as dull, aching or nagging, **poorly localized facial and/or oral pain**, which recurs daily for >2 h over >3 months. Only rarely are there electric-shock-like pain attacks as in trigeminal neuralgia.

PIFP is rare, mostly affecting younger women, but it can start at any age.

***Principles of management***

- ▪ PIFP is painful, and can be physically, psychologically and socially **debilitating**.
- ▪ It is often difficult to manage, often has comorbidities, and usually requires **specialist referral** in the first instance.
- ▪ Good treatment begins with **education of patients**, explaining their disorder and the purpose and means of management.
- ▪ Freedom from pain is difficult to achieve: the **objectives** in management, by medical, physical and/or psychological therapies, are reduction of pain intensity and developing patients’ coping mechanisms.
- ▪ Treatment is **prophylactic**: acute therapies (opioids or other analgesics) have no place in management of PIFP.

***Education of patients***

A patient information leaflet on persistent idiopathic facial pain is provided here in Section 4 (also, Additional file 27).

**Key points** of information are:

- ▪ PIFP is most often a **constant, dull, nagging or aching pain** in the cheek and lower jaw. Rarely there are electric-shock-like pains also.
- ▪ There are **no specific triggers**.
- ▪ The **causes** are unknown.
- ▪ There are **no tests** to confirm the diagnosis.
- ▪ **Preventative medications**, taken every day, are the best treatments for most people with PIFP:
  - ▪ these medications are more commonly used as antidepressants, but are very useful against chronic pain disorders even in people who are not depressed;
  - ▪ **painkillers are unhelpful** and, if taken too often, are likely to make things worse.

***Preventative medications***

Drugs with some efficacy are shown in Table 17. **Maximum dosages** may be necessary.

- ▪ Use of drugs off-licence rests on individual clinical responsibility.

**Table 17:** Drugs used in prophylaxis of persistent idiopathic facial pain

First line: • Amitriptyline or nortriptyline, 10–100 mg at night	• Intolerance is reduced by starting at a low dose (10 mg) and incrementing by 10–25 mg every 1–2 weeks; • Nortriptyline has fewer anticholinergic side-effects but less good evidence of efficacy
Second line (either as monotherapy or as add-on medication): • Gabapentin 600–3600 mg daily • Pregabalin 150–600 mg daily	

***Principles of prophylaxis***

- ▪ Patients receiving medication more often used as an antidepressant should be **advised of this, and why**; otherwise, they may default on finding out.
- ▪ Dosages should be **up-titrated slowly** until pain relief is achieved or side effects become unacceptable.
- ▪ **Combinations** may cause fewer side-effects because lower doses may be required of each drug.

***Follow-up***

While every patient with PIFP requires specialist initial management, long-term follow-up once stable is appropriate in primary care.

### Guides to referral

#### Headache management in primary care: when to refer

This guide can be separately downloaded (Additional file 14).

Most headache disorders presenting to primary care are migraine, tension-type headache or medication-overuse headache. These, usually, can be and are best managed in primary care.

##### Reasons for specialist referral

- ▪ **Diagnostic uncertainty** after due enquiry.
- ▪ Diagnosis of **any of the following**, which are best managed by specialists:
  - ▪ migraine with aura including motor weakness;
  - ▪ chronic migraine;
  - ▪ cluster headache;
  - ▪ trigeminal neuralgia;
  - ▪ persistent idiopathic facial pain.
- ▪ **Suspicion of serious secondary headache**, or of serious pathology where investigation may be necessary and is not available in primary care:
  - ▪ **progressively worsening** headache over weeks or longer;
  - ▪ headache brought on by **coughing**, **exercise** or **sexual activity**;
  - ▪ headache **associated with** any of the following:
    - ▪ **postural change** indicative of high or low intracranial pressure;
    - ▪ unexplained **fever**;
    - ▪ **stiffness of the neck**;
    - ▪ unexplained **focal neurological symptoms or signs** or with epileptic seizures;
    - ▪ **disorder of consciousness or memory**, or **change in personality**;
    - ▪ **weight-loss** or poor general condition;
  - ▪ **new headache**:
    - ▪ in any patient that is **thunderclap** in nature (intense headache with abrupt or “explosive” onset);
    - ▪ that is **daily and persistent from onset** in a patient without a prior history of headache;
    - ▪ in a patient **older than 50** years;
    - ▪ in a patient with a history of **cancer**;
    - ▪ in a patient with a history of **immunodeficiency** (including HIV infection);
    - ▪ in a patient with a history of **polymyalgia rheumatica**;
    - ▪ in a patient with a family history of **glaucoma**;
  - ▪ **unusual migraine aura**, especially:
    - ▪ prolonged aura (duration >1 h);
    - ▪ aura featuring brainstem symptoms and/or motor weakness;
    - ▪ new aura without headache in a patient older than 50 years and in the absence of a prior history of migraine.
- ▪ Persistent management failure.
- ▪ **Comorbid disorders** requiring specialist management.

## Instruments and other materials to aid diagnosis and management of headache disorders in primary care

### Introduction

Headache disorders are common, and the second-highest cause of disability in Europe [5]. Migraine, tension-type headache (TTH) and medication-overuse headache (MOH) are particularly important because they are common and responsible for almost all burden attributed to headache [5, 6].

Management of these belongs largely in primary care [1], partly because of the numbers involved but also because it is usually not difficult, requiring neither specialist skills nor investigations. Yet, throughout Europe and elsewhere, health-care providers in primary care may have received limited training in the diagnosis and treatment of headache [1]. The instruments and other materials collated here are developed, mostly by *Lifting The Burden*, specifically to aid primary-care physicians in both diagnosis and management. They should be used in conjunction with *European principles of management of headache disorders in primary care* (see Section 2, and Additional files 1, 2, 3, 4, 5, 6, 7, 8, 9, 10, 11, 12, 13 and 14).

The following are included here:

- ▪ 3.2 Diagnostic criteria for headache disorders in primary care: International Classification of Headache Disorders, 3rd edition (ICHD-3) – abbreviated form) (also, Additional file 15);
- ▪ 3.3 Headache diary and calendar to aid diagnosis and follow-up in primary care) (also, Additional files 16 and 17);
- ▪ 3.4 The Headache-Attributed Lost Time (HALT) Indices: measures of burden for headache management in primary care) (also, Additional files 18 and 19);
- ▪ 3.5 The Headache Under-Response to Treatment (HURT) questionnaire: a guide to follow-up in primary care) (also, Additional file 20);

While intended for use in primary care, these instruments and materials may also be useful in specialist practice.

Additionally, in Section 4 are Additional files 21, 22, 23, 24, 25, 26 and 27 *Patient information leaflets to aid headache management in primary care (2nd edition)*.

### Diagnostic criteria for headache disorders in primary care: the International Classification of Headache Disorders, 3rd edition (ICHD-3) – abbreviated form

This aid can be separately downloaded (Additional file 15).

#### Introduction

Headache disorders are common, and the second highest cause of disability worldwide (after low back pain) [5].

The International Classification of Headache Disorders, 3rd edition (ICHD-3), published by the International Headache Society [4], is the authoritative catalogue of headache disorders. It describes over 200 distinct headache types, subtypes or subforms, and incorporates explicit diagnostic criteria for each one.

Only a small number of these disorders are important in primary care. The purpose of this diagnostic aid, an adaptation of ICHD-3 specifically for primary care, is to help primary-care physicians recognise and correctly diagnose these. It sets out the diagnostic criteria for the three primary headache disorders (with seven types or subtypes), nine secondary headaches and two facial pains that are most likely to be seen in primary care or are important because they are symptomatic of another serious underlying disorder.

##### How the system works

This diagnostic aid should be used **as a reference**.

The classification distinguishes between **primary headaches**, which have no other underlying causative disorder, and **secondary headaches**, which are attributed to some other disorder. Onset in close temporal relation to another disorder known to cause headache is therefore a diagnostic criterion for all secondary headaches.

The third section of the classification covers **painful cranial neuropathies and other facial pain**.

All diagnoses are numbered according to their position within the classification hierarchy. In this abbreviated version, numbers are not consecutive because many headaches are not included.

Diagnoses are made by applying the criteria set out in the classification. A diagnosis is confirmed only when **all criteria for that disorder are fulfilled**. However, symptoms may have been modified by treatment, and this possibility should be considered in deciding whether criteria are met.

One patient may simultaneously have **two or more headache disorders**. Each should be separately diagnosed because each may require separate management.

The presence of more than one headache disorder can cause confusion, especially when a patient fails to distinguish between them. When this is suspected, it is recommended that he or she prospectively fills out a diagnostic headache diary, for a month or longer, recording the important characteristics of each headache episode. Diaries not only improve diagnostic accuracy but also allow precise judgment of medication consumption. A diary is included here among the management aids (see 3.3 *Headache diary and calendar to aid diagnosis and follow-up in primary care* (also, Additional file 16)).

#### Definitions of common terms

*Attack of headache (or pain):*

Headache (or pain) that builds up, remains at a certain level for minutes to 72 h, then wanes until it is gone completely.

*Attributed to:*

This term in ICHD-3 describes the relationship between a secondary headache and the disorder believed to cause it. It requires fulfilment of criteria establishing an accepted level of evidence of causation.

*Close temporal relation:*

This term is used to describe the relation between an organic disorder and a secondary headache attributed to it.

*Duration of attack:*

Time from onset until termination of an attack of headache (or pain) meeting criteria for a particular headache type or subtype. When the patient falls asleep during an attack and wakes up relieved, duration is until time of awakening. When an attack of migraine is successfully relieved by medication but symptoms recur within 48 h, these may represent a relapse of the same attack or a new attack (see *Frequency of attacks*).

*Facial pain:*

Pain below the orbitomeatal line, above the neck and anterior to the pinnae.

*Fortification spectrum:*

Angulated, arcuate and gradually enlarging visual disturbance typical of migrainous aura.

*Frequency of attacks:*

The rate of occurrence of attacks of headache (or pain) per time period (commonly 1 month). Successful relief of a migraine attack with medication may be followed by relapse within 48 h. The IHS *Guidelines for Controlled Trials of Drugs in Migraine, 3rd edition*, recommend as a practical solution, especially in differentiating attacks recorded as diary entries over the previous month, to count as distinct attacks only those that are separated by at least 48 h headache-free.

*Headache:*

Pain located in the head, above the orbitomeatal line and/or nuchal ridge.

*Headache days:*

Number of days during an observed period of time (commonly 1 month) affected by headache for any part or the whole of the day.

*Intensity of pain:*

Level of pain, usually scored on a four-point numerical rating scale (0–3) equivalent to no, mild, moderate and severe pain, or on a visual analogue scale (commonly 10 cm). It may also be scored on a verbal rating scale expressed in terms of its functional consequence: 0, no pain; 1, mild pain, does not interfere with usual activities; 2, moderate pain, inhibits but does not wholly prevent usual activities; 3, severe pain, prevents all activities.

*New headache:*

Any type, subtype or subform of headache from which the patient was not previously suffering.

*Persistent:*

This term, used in the context of certain secondary headaches, describes headache, initially acute and caused by another disorder, that fails to remit within a specified time interval (usually 3 months) after that disorder has resolved.

*Phonophobia:*

Hypersensitivity to sound, even at normal levels, usually causing avoidance.

*Photophobia:*

Hypersensitivity to light, even at normal levels, usually causing avoidance.

*Pressing/tightening:*

Pain of a constant quality, often compared to a tight band around the head.

*Primary headache (disorder):*

Headache, or a headache disorder, not caused by or attributed to another disorder. It is distinguished from secondary headache disorder.

*Pulsating:*

Characterized by rhythmic intensifications in time with the heart beat; throbbing.

*Scintillation:*

Visual hallucinations that are bright and fluctuate in intensity, often at approximately 8–10 Hz. They are typical of migraine aura.

*Scotoma:*

Loss of part(s) of the visual field of one or both eyes. Scotoma may be absolute (no vision) or relative (obscured or reduced vision). In migraine, scotomata are homonymous.

*Secondary headache (disorder):*

Headache, or a headache disorder, caused by another underlying disorder. In ICHD-3, secondary headaches are *attributed to* the causative disorder. Secondary headaches are distinguished from primary headaches. A secondary headache may have the characteristics of a primary headache but still fulfil criteria for causation by another disorder.

#### Primary headaches

**1. Migraine**

Migraine is a common disabling primary headache disorder. In the *Global Burden of Disease Survey 2010* (GBD 2010), it was ranked as the third most prevalent disorder in the world. In GBD 2015, it was ranked third-highest cause of disability worldwide in both males and females under the age of 50 years.

Migraine has two major types. 1.1 *Migraine without aura* is a clinical syndrome characterized by headache with specific features and associated symptoms. 1.2 *Migraine with aura* is primarily characterized by the transient focal neurological symptoms that usually precede but sometimes accompany the headache. Some patients, with either type, also experience a prodromal phase, occurring hours or days before the headache, and/or a postdromal phase following headache resolution. Common prodromal symptoms include fatigue, elated or depressed mood, unusual hunger and cravings for certain foods; postdromal include fatigue, elated or depressed mood and cognitive difficulties.

When a patient fulfils criteria for both these types of migraine, both should be diagnosed.

A third type, 1.3 *Chronic migraine*, is much less common but very highly disabling.

***1.1 Migraine without aura***

*Description:*

A recurrent headache disorder manifesting in attacks lasting 4–72 h. Typical characteristics of the headache are unilateral location, pulsating quality, moderate or severe intensity, aggravation by routine physical activity and association with nausea and/or photophobia and phonophobia.

*Diagnostic criteria:*

A. At least five attacks fulfilling criteria B-D
B. Headache attacks lasting 4–72 h (when untreated)^1^
C. Headache has at least two of the following four characteristics:
  1. unilateral location
  2. pulsating quality
  3. moderate or severe pain intensity
D. aggravation by or causing avoidance of routine physical activity (*eg*, walking or climbing stairs)
E. During headache at least one of the following:
  1. nausea and/or vomiting
  2. photophobia and phonophobia
F. Not better accounted for by another ICHD-3 diagnosis.

*Note:*

1. In children and adolescents (aged under 18 years), attacks may last 2–72 h.

***1.2 Migraine with aura***

*Description:*

Recurrent attacks, lasting minutes, of unilateral fully-reversible visual, sensory or other central nervous system symptoms that usually develop gradually and are usually followed by headache and associated migraine symptoms.

*Diagnostic criteria:*

A. At least two attacks fulfilling criteria B and C
B. One or more of the following fully reversible aura symptoms:
  1. visual
  2. sensory
  3. speech and/or language
  4. motor, brainstem and/or retinal^1^
C. At least three of the following six characteristics:
  1. at least one aura symptom spreads gradually over ≥5 min
  2. two or more aura symptoms occur in succession
  3. each individual aura symptom lasts 5–60 min
  4. at least one aura symptom is unilateral^2^
  5. at least one aura symptom is positive^3^
  6. the aura is accompanied, or followed within 60 min, by headache^4^
D. Not better accounted for by another ICHD-3 diagnosis.

*Notes:*

1. Motor, brainstem and retinal symptoms are atypical, occurring in specific subtypes of migraine with aura, and should lead to referral.
2. Aphasia is regarded as a unilateral symptom.
3. Scintillations and pins and needles are positive symptoms of aura.
4. *Typical aura without headache* is a recognised subtype but, in the absence of headache, the diagnosis of aura and its **distinction from mimics that may signal serious disease** (*eg*, transient ischaemic attack) becomes more difficult and often requires investigation.

***1.3 Chronic migraine***

*Description:*

Headache occurring on 15 or more days/month for more than 3 months, which, on at least 8 days/month, has the features of migraine headache.

*Diagnostic criteria:*

A. Headache (migraine-like or tension-type-like^1^) on ≥15 days/month for >3 months, and fulfilling criteria B and C
B. Occurring in a patient who has had at least five attacks fulfilling criteria B-D for 1.1 *Migraine without aura* and/or criteria B and C for 1.2 *Migraine with aura*
C. On ≥8 days/month for >3 months, fulfilling any of the following^2^:
  1. criteria C and D for 1.1 *Migraine without aura*
  2. criteria B and C for 1.2 *Migraine with aura*
  3. believed by the patient to be migraine at onset and relieved by a triptan or ergot derivative
D. Not better accounted for by another ICHD-3 diagnosis^3, 4^.

*Notes:*

1. It is impossible to distinguish the individual episodes of headache in patients with such frequent or continuous headaches. In this situation, attacks with and those without aura are both counted in diagnosing 1.3 *Chronic migraine*, as are both migraine-like and tension-type-like headaches.
2. Characterization of frequently recurring headache generally requires a headache diary to record information on pain and associated symptoms day-by-day for at least 1 month.
3. Because tension-type-like headache is within the diagnostic criteria for 1.3 *Chronic migraine*, this diagnosis excludes the diagnosis of 2. *Tension-type headache* or its types.
4. The most common cause of symptoms suggestive of chronic migraine is medication overuse, as defined under 8.2 *Medication-overuse headache*. Around 50% of patients apparently with 1.3 *Chronic migraine* revert to an episodic migraine type after drug withdrawal; such patients are in a sense wrongly diagnosed as 1.3 *Chronic migraine*. Equally, many patients apparently overusing medication do not improve after drug withdrawal; the diagnosis of 8.2 *Medication-overuse headache* may be inappropriate for these. Therefore, patients meeting criteria for 1.3 *Chronic migraine* and for 8.2 *Medication-overuse headache* should be coded for both. After drug withdrawal, migraine will either revert to an episodic type or remain chronic, and should be re-diagnosed accordingly; either diagnosis may be rescinded.

**2. Tension-type headache**

This is the most common headache. In the *Global Burden of Disease Survey 2010* (GBD 2010), it was ranked as the second most prevalent disorder in the world (behind dental caries). Two types are important.

***2.2 Frequent episodic tension-type headache***

*Description:*

Frequent episodes of headache, typically bilateral, pressing or tightening in quality and of mild to moderate intensity, lasting minutes to days. The pain lacks the specific characteristics of migraine: it does not worsen with routine physical activity and is not associated with nausea, although either photophobia or phonophobia may be present.

*Diagnostic criteria:*

A. At least 10 episodes of headache occurring on 1–14 days/month on average for >3 months (≥12 and <180 days/year) and fulfilling criteria B-D
B. Lasting from 30 min to 7 days
C. At least two of the following four characteristics:
  1. bilateral location
  2. pressing or tightening (non-pulsating) quality
  3. mild or moderate intensity
  4. not aggravated by routine physical activity such as walking or climbing stairs
D. Both of the following:
  1. no nausea or vomiting
  2. no more than one of photophobia or phonophobia
E. Not better accounted for by another ICHD-3 diagnosis^1^.

*Note:*

1. 2.2 *Frequent tension-type headache* often coexists with 1.1 *Migraine without aura*, in which case both diagnoses should be given. A diagnostic headache diary may be required to separate them.

***2.3 Chronic tension-type headache***

*Description:*

A disorder evolving from frequent episodic tension-type headache, with daily or very frequent episodes of headache, typically bilateral, pressing or tightening in quality and of mild to moderate intensity, lasting hours to days, or unremitting. The pain does not worsen with routine physical activity, but may be associated with mild nausea, photophobia or phonophobia.

*Diagnostic criteria:*

A. Headache occurring on ≥15 days/month on average for >3 months (≥180 days/year), fulfilling criteria B-D
B. Lasting hours to days, or unremitting
C. At least two of the following four characteristics:
  1. bilateral location
  2. pressing or tightening (non-pulsating) quality
  3. mild or moderate intensity
  4. not aggravated by routine physical activity such as walking or climbing stairs
D. Both of the following:
  1. no more than one of photophobia, phonophobia or mild nausea
  2. neither moderate or severe nausea nor vomiting
E. Not better accounted for by another ICHD-3 diagnosis^1, 2^.

*Notes:*

1. Both 2.3 *Chronic tension-type headache* and 1.3 *Chronic migraine* require headache on 15 or more days/month. For 2.3 *Chronic tension-type headache*, headache must, on at least 15 days, meet criteria B-D for 2.2 *Frequent episodic tension-type headache*; for *1.3 Chronic migraine* headache must, on at least 8 days, meet criteria B-D for 1.1 *Migraine without aura*. A patient can therefore fulfil all criteria for both these diagnoses, for example by having headache on 25 days/month meeting migraine criteria on 8 days and tension-type headache criteria on 17 days. In these cases, only the diagnosis 1.3 *Chronic migraine* should be given.
2. In many uncertain cases there is overuse of medication. When this fulfils criterion B for any of the subtypes of 8.2 *Medication-overuse headache* and the criteria for 2.3 *Chronic tension-type headache* are also fulfilled, both disorders should be diagnosed. After drug withdrawal, there may be reversion to episodic tension-type headache. When the disorder remains chronic after withdrawal, the diagnosis of 8.2 *Medication-overuse headache* may be rescinded.

**3. Trigeminal autonomic cephalalgias**

This group of uncommon disorders shares the clinical features of short-duration headache and prominent cranial parasympathetic autonomic features. Only one, with a prevalence of one per 1000 in males and lower in females, is expected to be seen and diagnosed in primary care. The others are even rarer and, if seen, may be mistaken for it. All should be referred for specialist management in the first instance.

***3.1 Cluster headache***

*Description:*

Attacks of severe, strictly unilateral pain which is orbital, supraorbital, temporal or in any combination of these sites, lasting 15–180 min and occurring from once every other day to eight times a day. The pain is associated with ipsilateral conjunctival injection, lacrimation, nasal congestion, rhinorrhoea, forehead and facial sweating, miosis, ptosis and/or eyelid oedema, and/or with restlessness or agitation.

*Diagnostic criteria:*

A. At least five attacks fulfilling criteria B-D
B. Severe or very severe unilateral orbital, supraorbital and/or temporal pain lasting 15–180 min (when untreated)
C. Either or both of the following:
  1. at least one of the following symptoms or signs, ipsilateral to the headache:
    - conjunctival injection and/or lacrimation
    - nasal congestion and/or rhinorrhoea
    - eyelid oedema
    - forehead and facial sweating
    - miosis and/or ptosis
  2. a sense of restlessness or agitation
D. Occurring with a frequency between one every other day and 8 per day
E. Not better accounted for by another ICHD-3 diagnosis.

Two subtypes are important.

***3.1.1 Episodic cluster headache***

*Description:*

Cluster headache attacks occurring in periods lasting from 7 days to 1 year, separated by pain-free periods lasting at least 3 months.

*Diagnostic criteria:*

A. Attacks fulfilling criteria for 3.1 *Cluster headache* and occurring in bouts (cluster periods)
B. At least two cluster periods lasting from 7 days to 1 year (when untreated) and separated by pain-free remission periods of ≥3 months.

***3.1.2 Chronic cluster headache***

*Description:*

Cluster headache attacks occurring for 1 year or longer without remission, or with remission periods lasting less than 3 months.

*Diagnostic criteria:*

A. Attacks fulfilling criteria for 3.1 *Cluster headache*, and criterion B below
B. Occurring without a remission period, or with remissions lasting <3 months, for at least 1 year.

#### Secondary headaches

Secondary headache disorders have another causative disorder underlying them; therefore, the headache occurs in close temporal relation to the other disorder, and/or worsens or improves in parallel with worsening or improvement of that disorder. These associations are keys to their diagnosis.

*General diagnostic criteria for secondary headaches:*

A. Any headache fulfilling criterion C
B. Another disorder scientifically documented to be able to cause headache has been diagnosed^1, 2^
C. Evidence of causation demonstrated by at least two of the following:
  1. headache has developed in temporal relation to the onset of the presumed causative disorder
  2. either or both of the following:
    - headache has significantly worsened in parallel with worsening of the presumed causative disorder
    - headache has significantly improved in parallel with improvement of the presumed causative disorder
  3. headache has characteristics typical for the causative disorder
  4. other evidence exists of causation
D. Not better accounted for by another ICHD-3 diagnosis.

*Notes:*

1. The diagnostic criteria for secondary headache disorders **do not set out criteria for diagnosing the underlying disorder**.
2. This criterion may require tests or procedures that cannot be undertaken in primary care. In such cases, the diagnosis cannot be confirmed in primary care. The crucial role of primary care is to recognise the possibility of the diagnosis.

The secondary headaches described below are those that are common or otherwise important (must not be missed) in primary care.

**5. Headache attributed to trauma or injury to the head and/or neck**

***5.2 Persistent headache attributed to traumatic injury to the head***

Persistent post-traumatic headache is often part of the post-traumatic syndrome, which includes symptoms such as equilibrium disturbance, poor concentration, decreased work ability, irritability, depressive mood and sleep disturbances.

*Description:*

Headache of more than 3 months’ duration caused by traumatic injury to the head.

*Diagnostic criteria:*

A. Any headache fulfilling criteria C and D
B. Traumatic injury to the head has occurred
C. Headache is reported to have developed within 7 days after one of the following:
  1. the injury to the head
  2. regaining of consciousness following the injury to the head
  3. discontinuation of medication(s) impairing ability to sense or report headache following the injury to the head
D. Headache persists for >3 months after its onset
E. Not better accounted for by another ICHD-3 diagnosis^1^.

*Note:*

1. When headache following head injury becomes persistent, the possibility of 8.2 *Medication-overuse headache* needs to be considered.

**6. Headache attributed to cranial and/or cervical vascular disorder**

***6.2.2 Acute headache attributed to non-traumatic subarachnoid haemorrhage***

Non-traumatic subarachnoid haemorrhage (SAH) is one of the most common causes of persistent, intense and incapacitating headache of abrupt onset (thunderclap headache). It is a serious condition, and delayed diagnosis often has a catastrophic outcome: mortality is 40–50%, with 10–20% of patients dying before arriving at hospital; 50% of survivors are left disabled.

*Description:*

Headache caused by non-traumatic SAH, typically severe and sudden in onset, peaking in seconds (thunderclap headache) or minutes. It can be the sole symptom of non-traumatic SAH.

*Diagnostic criteria:*

A. Any new headache fulfilling criteria C and D
B. SAH in the absence of head trauma has been diagnosed
C. Evidence of causation demonstrated by at least two of the following:
  1. headache has developed in close temporal relation to other symptoms and/or clinical signs of SAH, or has led to the diagnosis of SAH
  2. headache has significantly improved in parallel with stabilization or improvement of other symptoms or clinical or radiological signs of SAH
  3. headache has sudden or thunderclap onset
D. Either of the following:
  1. headache has resolved within 3 months
  2. headache has not yet resolved but 3 months have not yet passed
E. Not better accounted for by another ICHD-3 diagnosis.

***6.4.1 Headache attributed to giant cell arteritis***

Giant cell arteritis (GCA) is conspicuously associated with headache, but its characteristics are variable. GCA must be recognized: any persisting headache with recent onset in a patient over 60 years of age should suggest it. Recent repeated attacks of amaurosis fugax associated with headache are very suggestive of GCA. Blindness is a major risk, but preventable by immediate steroid treatment. The time interval between visual loss in one eye and in the other is usually less than 1 week.

*Description:*

Headache, with variable features, caused by and symptomatic of GCA. Headache may be the sole symptom of GCA, a disease most conspicuously associated with headache.

*Diagnostic criteria:*

A. Any new headache fulfilling criterion C
B. GCA has been diagnosed
C. Evidence of causation demonstrated by at least two of the following:
  1. headache has developed in close temporal relation to other symptoms and/or clinical or biological signs of onset of GCA, or has led to the diagnosis of GCA
  2. either or both of the following:
    - headache has significantly worsened in parallel with worsening of GCA
    - headache has significantly improved or resolved within 3 days of high-dose steroid treatment
  3. headache is associated with scalp tenderness and/or jaw claudication
D. Not better accounted for by another ICHD-3 diagnosis.

**7. Headache attributed to non-vascular intracranial disorder**

***7.2 Headache attributed to low cerebrospinal fluid pressure***

*Description:*

Headache caused by low cerebrospinal fluid (CSF) pressure, usually orthostatic and accompanied by neck pain, tinnitus, changes in hearing, photophophia and/or nausea. It remits after normalization of CSF pressure.

Three subtypes are distinguished by aetiology: following-recent dural puncture, attributed to persistent CSF leakage (CSF fistula) or spontaneous.

*Diagnostic criteria:*

A. Any headache^1^ fulfilling criterion C
B. Either or both of the following:
  1. low CSF pressure (<60 mm CSF)
  2. evidence of CSF leakage on imaging
C. Headache has developed in temporal relation to the low CSF pressure or CSF leakage, or led to its discovery
D. Not better accounted for by another ICHD-3 diagnosis.

*Note:*

1. 7.2 *Headache attributed to low cerebrospinal fluid pressure* is usually but not invariably orthostatic. Headache that significantly worsens soon after sitting upright or standing and/or improves after lying horizontally is likely to be caused by low CSF pressure, but this cannot be relied upon as a diagnostic criterion.

***7.4.1 Headache attributed to intracranial neoplasm***

Headache is a common symptom of intracranial tumours, more so in young patients (including children), but it rarely remains the only symptom: neurological deficits and seizures are common.

*Description:*

Headache caused by one or more space-occupying intracranial tumours.

*Diagnostic criteria:*

A. Any headache^1^ fulfilling criterion C
B. A space-occupying intracranial neoplasm has been demonstrated
C. Evidence of causation demonstrated by at least two of the following:
  1. headache has developed in temporal relation to development of the neoplasm, or led to its discovery
  2. either or both of the following:
    - headache has significantly worsened in parallel with worsening of the neoplasm
    - headache has significantly improved in temporal relation to successful treatment of the neoplasm
  3. headache has at least one of the following four characteristics:
    - progressive
    - worse in the morning and/or when lying down
    - aggravated by Valsalva-like manœuvres
    - accompanied by nausea and/or vomiting
D. Not better accounted for by another ICHD-3 diagnosis.

*Note:*

1. There are no pathognomonic features of 7.4.1 *Headache attributed to intracranial neoplasm*, although progression or deterioration is a key feature. The other suggestive symptoms (severe, worse in the morning and associated with nausea and vomiting) are not a classical triad; they are more likely in the context of intracranial hypertension and with posterior fossa tumours. Nevertheless, a history indicating raised intracranial pressure should first suggest intracranial neoplasm.

**8. Headache attributed to a substance or its withdrawal**

***8.1.3 Carbon monoxide-induced headache***

Carbon monoxide intoxication is particularly associated with headache, which, at low levels of exposure, may be the only symptom. Usually resulting from open fires or faulty gas boilers in the home, it is not rare in some countries, and likely to present to primary care.

*Description:*

Headache caused by exposure to carbon monoxide (CO), resolving spontaneously within 72 h after its elimination.

Dependent on carboxyhaemoglobin level, headache ranges from mild without other symptoms, through moderate and pulsating with irritability, to severe with nausea, vomiting, blurred vision and, ultimately, impaired consciousness.

*Diagnostic criteria:*

A. Bilateral headache fulfilling criterion C
B. Exposure to CO has occurred
C. Evidence of causation demonstrated by all of the following:
  1. headache has developed within 12 h of exposure to CO
  2. headache intensity varies with the severity of CO intoxication
  3. headache has resolved within 72 h of elimination of CO
D. Not better accounted for by another ICHD-3 diagnosis.

**8.2 Medication-overuse headache**

This disorder occurs in patients chronically overusing medication to treat a prior headache disorder, usually 1. *Migraine* or 2. *Tension-type headache*; both the prior headache and 8.2 *Medication-overuse headache* (MOH) should be diagnosed.

Correct diagnosis of MOH is important because patients will not improve without withdrawal of the offending medication. On the other hand, most patients with MOH improve after withdrawal, as does their responsiveness to preventative treatment.

*Description:*

Headache occurring on 15 or more days/month in a patient with a pre-existing primary headache and developing as a consequence of regular overuse of acute or symptomatic headache medication for more than 3 months. It usually, but not invariably, resolves after the overuse is stopped.

*Diagnostic criteria:*

A. Headache occurring on ≥15 days/month in a patient with a pre-existing headache disorder
B. Regular overuse for >3 months of one or more drugs that can be taken for acute and/or symptomatic treatment of headache^1, 2^
C. Not better accounted for by another ICHD-3 diagnosis.

*Notes:*

1. Drugs may be ergotamine, one or more triptans, non-opioid analgesics including paracetamol (acetaminophen), acetylsalicylic acid and other non-steroidal anti-inflammatory drugs (NSAIDs), opioids, combination analgesics (typically containing simple analgesics plus opioids, butalbital and/or caffeine) or any combination of these.
2. Overuse is defined as intake on ≥15 days/month for non-opioid analgesics alone and *in all other cases* as intake on ≥10 days/month.

**9. Headache attributed to infection**

***9.1.1 Headache attributed to bacterial meningitis or meningoencephalitis***

Headache is the commonest and may be the first symptom of these infections, which should be suspected whenever headache is associated with fever, altered mental state, focal neurological deficits or generalized seizures.

*Description:*

Headache of variable duration caused by bacterial meningitis or meningoencephalitis. It may develop with mild flu-like symptoms and is typically acute and associated with neck stiffness, nausea, fever and changes in mental state and/or other neurological symptoms and/or signs.

In most cases, headache resolves with resolution of the infection. Rarely it persists (as the subform 9.1.1.3 *Persistent headache attributed to past bacterial meningitis or meningoencephalitis*) for more than 3 months after resolution of the infection.

*Diagnostic criteria:*

A. Headache of any duration fulfilling criterion C
B. Bacterial meningitis or meningoencephalitis has been diagnosed
C. Evidence of causation demonstrated by at least two of the following:
  1. headache has developed in temporal relation to the onset of the bacterial meningitis or meningoencephalitis
  2. headache has significantly worsened in parallel with worsening of the bacterial meningitis or meningoencephalitis
  3. headache has significantly improved in parallel with improvement in the bacterial meningitis or meningoencephalitis
  4. headache is either or both of the following:
    - holocranial
    - located in the nuchal area and associated with neck stiffness
D. Not better accounted for by another ICHD-3 diagnosis.

**11. Headache or facial pain attributed to disorder of the cranium, neck, eyes, ears, nose, sinuses, teeth, mouth or other facial or cervical structure**

***11.3.1 Headache attributed to acute angle-closure glaucoma***

Acute angle-closure glaucoma generally causes eye and/or periorbital pain, visual acuity loss (blurring), conjunctival injection and oedema, nausea and vomiting. As intraocular pressure rises, so does the risk of permanent visual loss. Early diagnosis is essential.

*Description:*

Headache, usually unilateral, caused by acute angle-closure glaucoma and associated with other symptoms and clinical signs of this disorder (eye and/or periorbital pain, visual acuity loss [blurring], conjunctival injection and oedema, nausea and vomiting).

*Diagnostic criteria:*

A. Any headache fulfilling criterion C
B. Acute angle-closure glaucoma has been diagnosed, with proof of increased intraocular pressure
C. Evidence of causation demonstrated by at least two of the following:
  1. headache has developed in temporal relation to the onset of the glaucoma
  2. headache has significantly worsened in parallel with progression of the glaucoma
  3. headache has significantly improved or resolved in parallel with improvement in or resolution of the glaucoma
  4. pain location includes the affected eye
D. Not better accounted for by another ICHD-3 diagnosis.

#### Painful cranial neuropathies and other facial pain

**13. Painful lesions of the cranial nerves and other facial pain**

***13.1.1 Trigeminal neuralgia***

The diagnosis of 13.1.1 *Trigeminal neuralgia* must be established clinically. Investigations are designed to identify cause.

*Description:*

A disorder characterized by recurrent unilateral brief electric shock-like pains, abrupt in onset and termination, limited to the distribution of one or more divisions of the trigeminal nerve and triggered by innocuous stimuli. It may develop without apparent cause or be a result of another disorder. Additionally, there may be concomitant continuous pain of moderate intensity within the distribution(s) of the affected nerve division(s).

*Diagnostic criteria:*

A. Recurrent paroxysms of unilateral facial pain in the distribution(s) of one or more divisions of the trigeminal nerve, with no radiation beyond, and fulfilling criteria B and C
B. Pain has all of the following characteristics:
  1. lasting from a fraction of a second to 2 min^1^
  2. severe intensity^2^
  3. electric shock-like, shooting, stabbing or sharp in quality
C. Precipitated by innocuous stimuli within the affected trigeminal distribution^3^
D. Not better accounted for by another ICHD-3 diagnosis.

*Notes:*

1. Paroxysms may become more prolonged over time.
2. Pain may become more severe over time.
3. Some attacks may be, or appear to be, spontaneous, but there must be a history or finding of pain provoked by innocuous stimuli to meet this criterion.

***13.12 Persistent idiopathic facial pain (PIFP)***

*Description:*

Persistent facial and/or oral pain, with varying presentations but recurring daily for more than 2 h/day over more than 3 months, in the absence of clinical neurological deficit.

*Persistent idiopathic facial pain* may be comorbid with other pain conditions such as chronic widespread pain and irritable bowel syndrome. In addition, it presents with high levels of psychiatric comorbidity and psychosocial disability.

*Diagnostic criteria:*

A. Facial and/or oral pain fulfilling criteria B and C
B. Recurring daily for >2 h/day for >3 months
C. Pain has both of the following characteristics:
  1. poorly localized, and not following the distribution of a peripheral nerve
  2. dull, aching or nagging quality
D. Clinical neurological examination is normal
E. A dental cause has been excluded by appropriate investigations
F. Not better accounted for by another ICHD-3 diagnosis.

### Headache diary and calendar to aid diagnosis and follow-up in primary care

These aids can be separately downloaded (Additional files 16 and 17).

#### Introduction

Good management of most headache disorders requires **monitoring of symptoms over time**. Diaries and calendars aid both patients and physicians. The principal distinction between these is in the amount of information collected.

**Diaries** capture more descriptive features of symptoms (headache intensity and character, associated symptoms), perhaps using free text. They are recommended in primary care, for 1–2 months, as aids to **diagnosis** and in **pre-treatment assessment**.

Specifically, **diaries are useful for** recording:

- ▪ symptoms and temporal patterns that contribute to correct **diagnosis**;
- ▪ acute medication use (class, dosage and frequency), identifying **base-line** usage or overuse;
- ▪ lost productive time as part of **pre-treatment assessment**.

Diaries are particularly helpful, and may be essential, in the diagnosis of conditions characterised by **headache on ≥15 days per month**, including medication-overuse headache.

**Calendars** essentially note the temporal occurrence of headache episodes and related events such as menstruation and medication intake. They are recommended in primary care **during follow-up**, once the headache is diagnosed.

Specifically, **calendars are useful for**:

- ▪ revealing **associations** with the menstrual cycle and, possibly, with other triggers;
- ▪ monitoring acute **medication use** or overuse during follow-up;
- ▪ encouraging **adherence** to prophylactic medication;
- ▪ recording treatment effect on headache frequency, and **charting outcomes**.

#### Diary and calendar for use in primary care

**Fig. 1:**
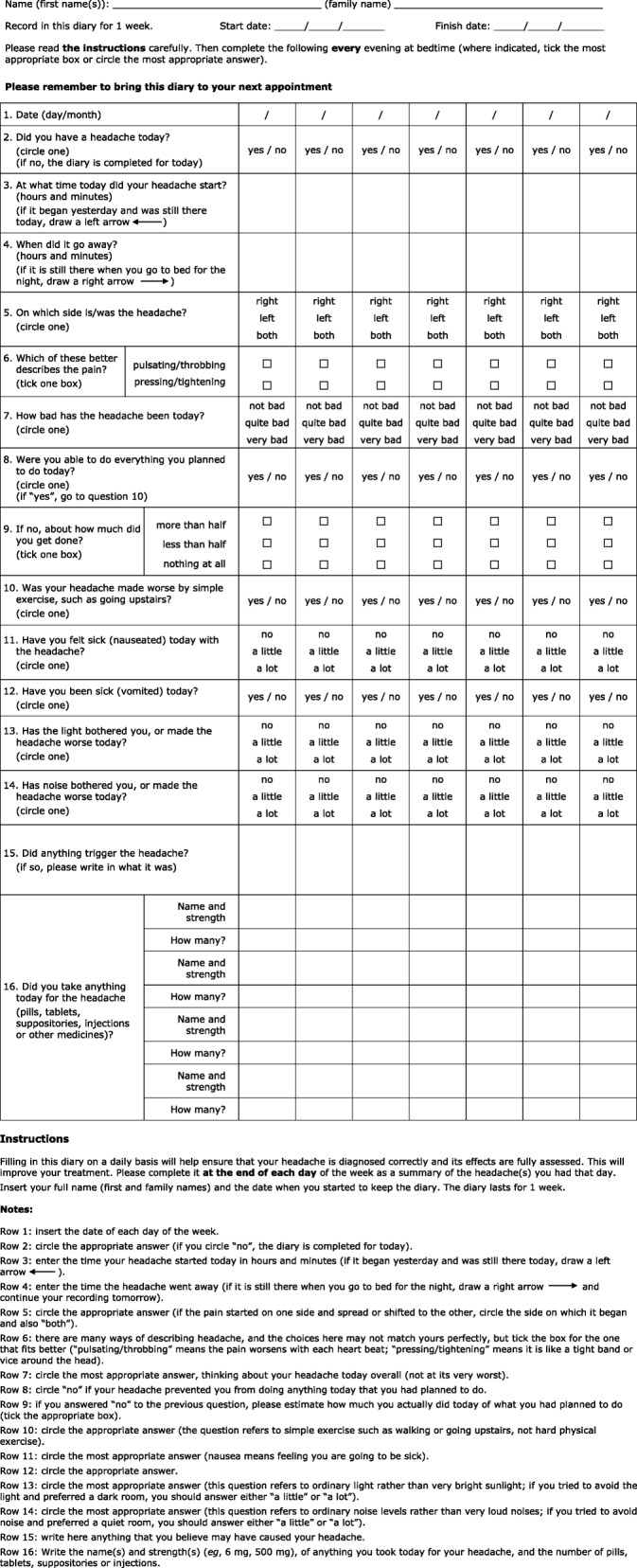
Diagnostic headache diary

**Fig. 2:**
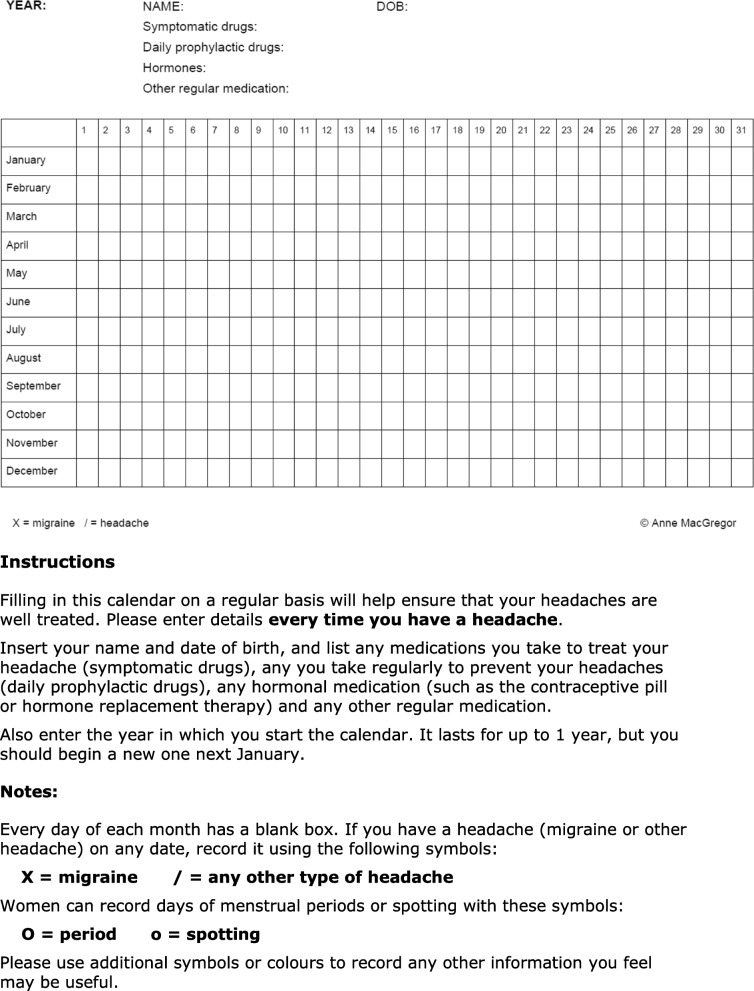
Headache calendar for follow-up

Many diaries and calendars have been developed, mostly in paper form. An example of each, developed by specialists in headache centres but useful in primary care, is included here (Figs. 1 and 2 (also, Additional files 16 and 17)).

#### On-line diaries and smartphone apps

There are many of these available, but of varying quality and utility. Some appear to gather data for marketing purposes.

On the other hand, some can be useful in establishing the characteristics of individual attacks, response to treatment and associations with potential triggers over time. Some, probably better suited to specialist care, enable data to be shared directly with health-care professionals.

### The Headache-Attributed Lost Time (HALT) Indices: measures of burden for headache management in primary care

These aids can be separately downloaded (Additional files 18 and 19).

#### Introduction

Assessment of a headache disorder requires more than diagnosis: there needs to be some **measure of impact** on the patient’s life and lifestyle, both as a prelude to planning best management and to establish the baseline against which to evaluate treatment.

The burden attributable to headache disorders has multiple components: there are many ways in which recurrent or persistent headache can damage life. No simple measure can summarise them all in a single index, but the Migraine Disability Assessment (MIDAS) instrument [7] has proved extremely useful. The concept behind MIDAS is estimation of productive time lost through the disabling effect of headache; the result is expressed by a number with intuitively meaningful units (*eg*, days/month).

Despite its name, MIDAS is not truly a measure of disability: unless headache is very severe, people have an element of choice in whether or not to take time out of work or other activities when affected by headache. One person may “work through”, another may not. Furthermore, the choice is likely to be influenced by external factors, such as availability of sickness pay. Nevertheless, because productive time is an important casualty of headache, its measurement is highly relevant to burden assessment.

#### The Headache-Attributed Lost Time (HALT) Indices

The HALT Index was first described in 2007 [8] as a direct and close derivative of MIDAS. It was developed by *Lifting The Burden* to use wording that is more easily translated than the American-English of MIDAS [7]. HALT has five questions similar to the first five questions of MIDAS.

Questions 1 and 2 ask about *absenteeism* due to headache and reduced productivity while at work despite headache (*presenteeism*). “Work” in this context may be as a paid employee or in self-employment. For children it includes schoolwork. To estimate total lost productive time from work, days **wholly lost through absenteeism** are added to **days of presenteeism with <50% productivity**; by way of counterbalance, headache-affected days are ignored in which productivity was nevertheless >50%. Questions 3 and 4 address household work in the same manner. “Household work” refers to the range of chores necessary in daily home living; while the nature of these may to an extent be gender-related, “household work” is not intended only to encompass work that tends, in many cultures, to be left to women (often termed “housework” in English).

An instruction is given to avoid double-counting (on a single day, productivity both at work and in the performance of housework may suffer reductions of >50%).

Question 5 relates to days on which social occasions are missed because of headache.

**Fig. 3:**
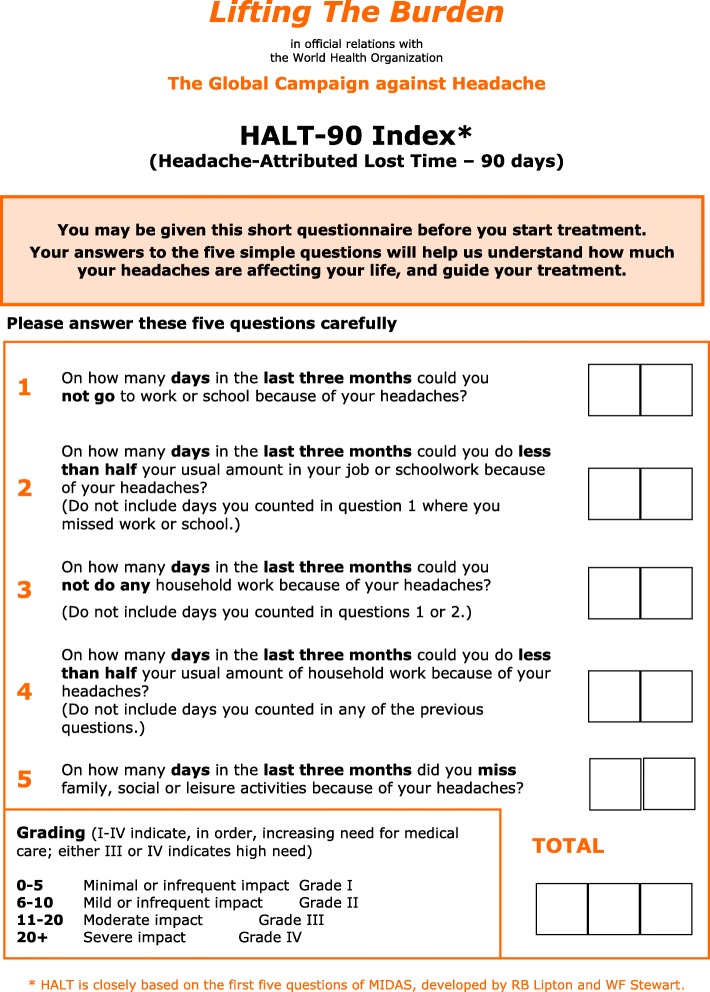
The Halt-90 Index

**Fig. 4:**
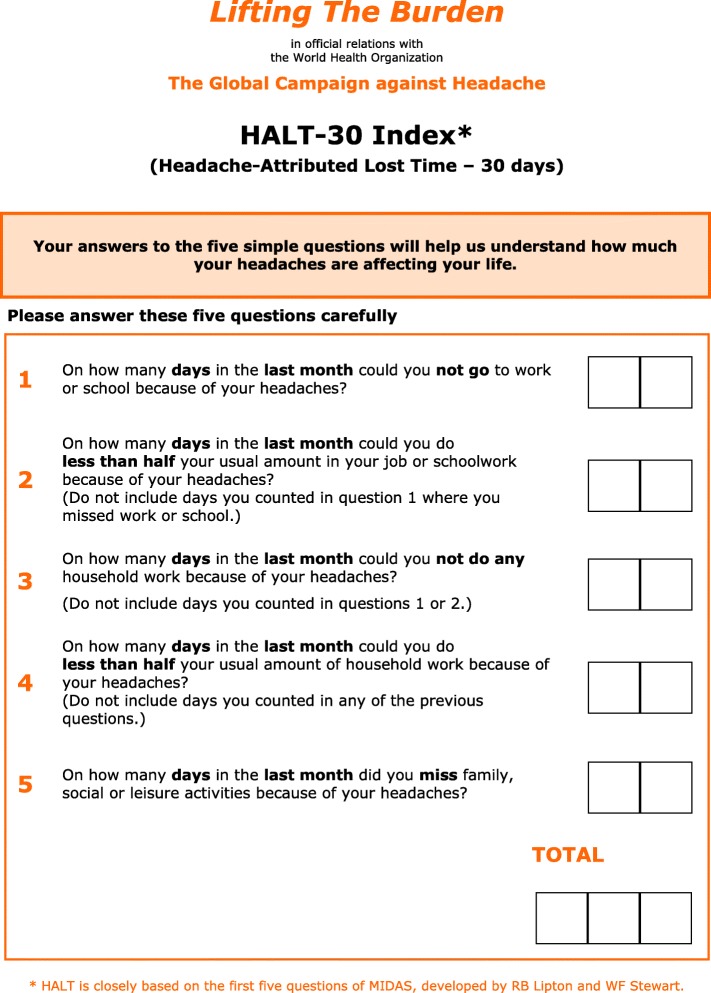
The Halt-30 Index

There are three versions of HALT [9]. Two of these, included here, are useful in headache management while serving different purposes. **HALT-90** (Fig. 3 (also, Additional file 18)) counts days affected by headache during the preceding 3 months (90 days). In the initial assessment of a patient, this best balances two conflicting demands: the need to reflect a patient’s illness over a representative period against the problems of recall error when that period is prolonged. During follow-up, the purpose of assessment shifts towards measurement of change attributable to treatment. Measures reflecting shorter periods than 3 months serve this purpose better: **HALT-30** (Fig. 4 (also, Additional file 19)) accordingly records days affected during the preceding 1 month (30 days).

##### Scoring HALT

HALT (30 or 90) can generate **three summed scores** from the first four questions (Figs. 3 and 4), the unit of each being whole days per one or 3 months:

- lost (paid) work time;
- lost household work time;
- total lost productive time – the sum of (a) and (b).

Question 5, however, gives rise to a simple count for which the unit is not whole days, and an error is introduced when this count is added to any of these scores. Furthermore, including question 5 in a summation of responses further invites double counting when a day lost at work is followed by a missed social event during the evening of the same day. Nevertheless, the count of lost social events *does* reflect additional burden, so question 5 is retained in HALT-90 (Fig. 3) and included in the **total summed score** (sum of all five questions), which gives rise to **grading**, as with MIDAS [7] (see Table 18).

**Table 18:** Grading of HALT-90^a^

Days lost in last 3 months	Assessed impact	Grade (indicating increasing need for medical care)
0–5	Minimal or infrequent	I
6–10	Mild or infrequent	II
11–20	Moderate	III (indicates high need for care)
≥20	Severe	IV (indicates high need for care)

^a^Following the grading of MIDAS [7]

Grading has value in indicating the level of a patient’s personal need and, perhaps, priority for treatment. But for assessment as a prelude to planning management, or for establishing the baseline impact, the individual summed scores are more informative than overall grades. Grading is not used by HALT-30.

### The Headache Under-Response to Treatment (HURT) questionnaire: a guide to follow-up in primary care

This aid can be separately downloaded (Additional file 20).

#### Introduction

Whenever treatment of a patient is started, or changed, **follow-up** either ensures that optimum treatment has been established or recognises that it has not. In the latter case, it should then identify any further change(s) to treatment that may be needed.

Resources, services and expectations vary greatly between countries and cultures. Even in optimal circumstances, outcomes are rarely perfect. It is not always easy to know whether or not the outcome that has been achieved by an individual patient is the best that the patient can reasonably expect. For the non-specialist, one question that sometimes arises is: “What further effort, in hope of a better outcome, is justified?” A second question, which follows if it is thought that more should be done, may be “What is it that needs changing?”

*Lifting The Burden* developed the **HURT questionnaire** [10] as an instrument that would not only assess outcome but also provide answers to these two questions, offering guidance to non-specialists on appropriate actions towards treatment optimisation.

#### The Headache Under-Response to Treatment (HURT) questionnaire

**Fig. 5:**
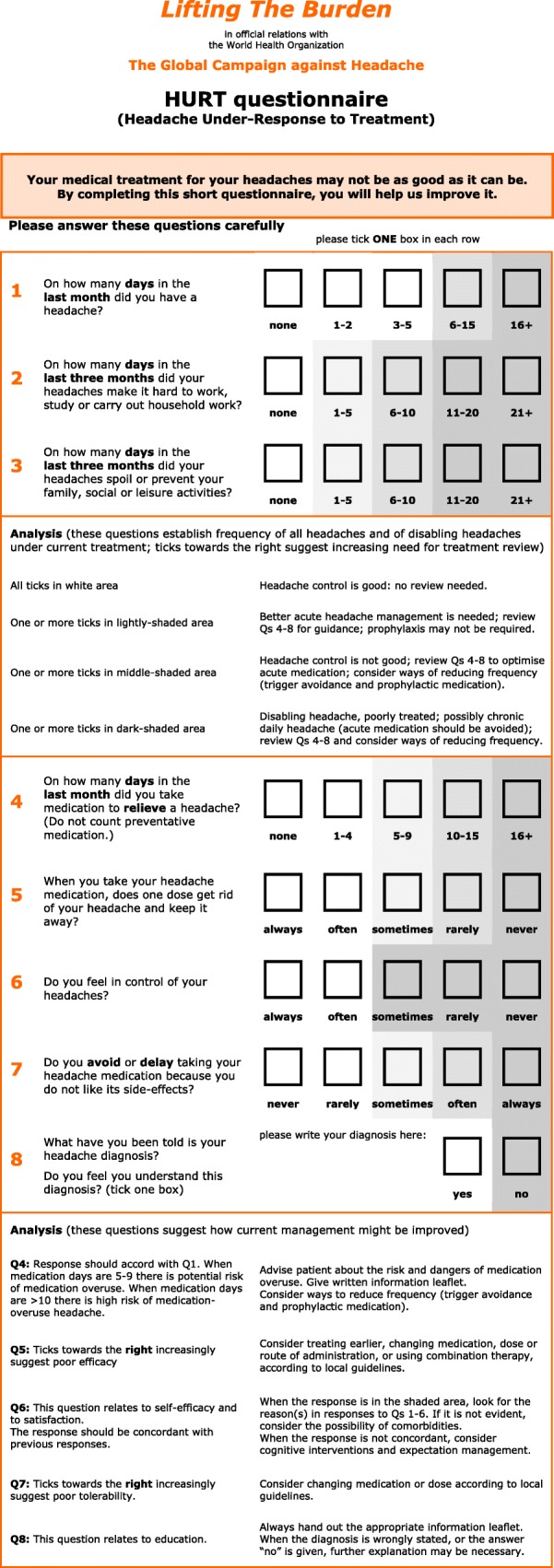
The HURT questionnaire

HURT is an **8-item self-administered questionnaire** (Fig. 5 (also, Additional file 20)): therefore, it is quick and easy to use in primary care.

It addresses headache frequency, disability, medication use and effect, patients’ perceptions of headache “control” and their understanding of their diagnosis. Responses are either numerated in days over a 1- or 3-month recall-period or selected from Likert options. In either case, responses either fall into an area of “no concern” or are graded into one of three flagged areas indicating increasingly important treatment deficiencies; clinical advice is provided for each of latter.

HURT has undergone psychometric validation and clinical testing in various settings and cultures [10].

## Patient information leaflets to aid headache management in primary care (2nd edition)

These leaflets can be downloaded from the Additional file (see below).

### Introduction

Headache management is greatly facilitated when the patient understands his or her headache disorder and the treatment being proposed for it. Adherence is improved and a better outcome is likely.

Good treatment of patients with any headache disorder therefore begins with **explanations of their disorder and the purpose and means of management**.

Many people with recurrent headache **wrongly fear underlying disease**.

- ▪ Explanation is a crucial element of preventative management in patients with frequent migraine or tension-type headache, who are at particular risk of **escalating medication consumption**.

**The general principles of headache management place education and reassurance of patients first**. These should never be omitted, but they take time, which is often not available. To assist, *Lifting The Burden* (LTB) has produced a series of Patient Information Leaflets (PILs).

### *Lifting The Burden*’s patient information leaflets

The purpose of LTB’s PILs is to provide the information and explanations to supplement any advice given directly by health-care providers. One or more may be handed to patients at the time of diagnosis, or later when needed.

This purpose requires all content to be:

- ▪ accurate;
- ▪ appropriate, comprehensive, informative and helpful;
- ▪ cross-culturally relevant and understandable.

In the original development of these PILs (first edition), LTB accordingly convened a writing and review group, drawn from all world regions, of headache specialists, primary-care physicians and patient representatives and advocates (see Acknowledgements, above).

**Seven PILs**, produced with the help of an expert panel (see Acknowledgements, above), constitute the second edition:

- ▪ revisions (second editions) of the four leaflets on the important headache disorders in primary care:
  - ▪ migraine (Additional file 21);
  - ▪ tension-type headache (Additional file 22);
  - ▪ cluster headache (Additional file 23);
  - ▪ medication-overuse headache (Additional file 24);
  - ▪ and of the fifth, explaining the relationships between female hormones and headache, which commonly raise questions from patients (Additional file 25);
- ▪ two new leaflets providing information for people affected by trigeminal neuralgia (Additional file 26) or persistent idiopathic facial pain (Additional file 27).

## Translation, and the preservation of original meaning, of materials developed to improve headache management

### Introduction

The Global Campaign against Headache aims to reduce the burden of headache worldwide. It is, by definition and action, a worldwide campaign, pursuing this aim through activities in many countries in a programme intended to improve access to effective and appropriate headache services [2, 11–13]. Foremost among the steps this requires is education about headache: both of health-care professionals and of people affected by headache disorders [1].

The programme also entails the production of a range of written materials, on the one hand to support education and on the other as aids to headache management delivered, in the main, by non-experts in primary care [1, 14]. These materials are invariably developed in English, but they need to be useful in health services in countries, and to people of many cultures, throughout the world.

Ready access by people everywhere requires translation into numerous languages. While it is said that 13 languages (Arabic, Bengali, Chinese [Mandarin], Dutch, English, German, French, Hindi/Urdu, Italian, Japanese, Portuguese, Russian and Spanish) can together reach half the world’s population, these languages are diverse, and translation is a technical challenge. Documents are produced for the Global Campaign with great care: translations should throw none of this away by failing to preserve their original meaning. The documents are of different types – some technical and some intended for lay users. When written materials are to become a supporting part of health care, the crucial importance of preserving meaning during translation becomes especially evident [15–17].

While the apparently simple aim of translation is to produce a translated version that is *equivalent* to the original version, “equivalence” in this context is not itself a simple concept. There is more than one type of equivalence. Predominant are semantic equivalence (equivalence in the meaning of words [15]) and conceptual equivalence (important in the case of an instrument required to measure the same theoretical construct in different languages [18]). A suggested essential requirement of translations is that they are *symmetrical*, which means that the original and translated versions not only are loyal to meaning but also use language that is equally *familiar* to the target populations [18].

The likelihood of achieving all of these is greatly enhanced when translation follows standardised protocols, and is underpinned by explicit quality-control procedures. Without these, there is rather low probability that translated products will carry and impart the same meaning as the originals to users from a wide variety of cultures.

Below we briefly describe good translation methodology, the different types of documents produced for the Global Campaign against Headache and the three protocols developed originally by an expert group convened by LTB [19]. We explain the purposes behind the protocols, and the importance of following them despite that they may appear somewhat onerous. We also update the protocols below, in a second edition (also, Additional files 28, 29 and 30).

These should be used from now on for all Campaign materials, whether related to clinical management, policy or research.

#### Translation methods

The different methods of translation aimed at securing quality include multiple forward translations with reconciliation, committee translation, and forward and back translation with reconciliation. International guidelines have tended to recommend forward and back translation [15, 16], used for example in translating the SF-36 in the International Quality of Life Assessment (IQOLA) project [20] and the EuroQoL five-dimensional questionnaire (EQ-5D) [21]. Specifically for instruments used in headache management, Peters and Passchier recommended the following steps to achieve high-quality translations [17]:

1. written guidance for translators and evaluators;
2. forward and back translation, using at least two forward translators and one back translator;
3. evaluation of translation for quality and equivalence;
4. pilot testing among a sample drawn from the target population (seeking comments on content and comprehensibility);
5. psychometric testing, when appropriate.

None of these steps ensures good quality per se, but they contribute collectively to a high level of control of the translation. This increases the likelihood of good translation, and of equivalence between the original and target-language versions. It should be noted that focus on the translation process alone is insufficient: evaluation by representatives of target users is necessary to complete quality *assurance*.

### *Lifting The Burden*’s approach, and three translation protocols

The methodological recommendations referred to above [15–18, 20, 21] were for instruments used in research rather than clinical management. LTB on the other hand creates three different types of document according to purpose (Table 19).

**Table 19:** The three types of document produced for the Global Campaign, the expert consensus group and their five essential criteria

Document types	Group members	Essential criteria
**Lay**, such as information leaflets for people with headache; **Technical**, expected to be read only by professionals and used in management: management guidelines are an example; **Hybrid**, to be read and understood by people with headache but used either in clinical practice or in research: examples are lay-administered diagnostic questionnaires, diagnostic or follow-up diaries, the HALT Indices (measures of impact) and the HURT questionnaire (an outcome measure).	**JM Bertolote**, Department of Mental Health and Substance Abuse, World Health Organization, Geneva, Switzerland; **C Houchin**, Oxford Outcomes Ltd., Oxford, UK; **T Kandoura**, Oriental Institute, University of Oxford, Oxford, UK; **M Peters**, Nuffield Department of Population Health, University of Oxford, Oxford, UK **TJ Steiner**, Division of Brain Sciences, Imperial College London, London, UK	The protocols must: • Conform to accepted translation guidelines; • Ensure rigour of the translation process and quality of the translated products; • Be suitable and have utility across different countries and cultures; • Include target-user evaluation; • Be pragmatic, recognising that unduly onerous protocols would be rejected and therefore unhelpful.

In 2007, LTB convened a consensus group, whose members combined expertise in cross-cultural translation and familiarity with the aims and endeavours of LTB, and charged them with developing translation protocols for each document type. The group adapted the earlier recommendations accordingly, producing three different protocols to suit the three types. In the process, they stipulated five essential criteria (Table 19) [19], to which all three protocols conform.

Although there are many similarities between the three protocols, key differences were introduced to make translation less onerous to the extent this was possible without compromising quality.

#### Translation protocol for lay documents (2nd edition)

This protocol can be separately downloaded (Additional file 28).

These guidelines are for the translation of documents (“lay documents”) produced for the Global Campaign against Headache as information for lay people, including people with headache, the general public and the lay media.

Translations of all lay documents should follow these guidelines to ensure a high quality of translation and to be approved by LTB.

##### Procedure

Translation should follow five steps.

***1. Coordination of the translation***

A translation coordinator, who oversees but does not carry out the translation, is selected according to the following criteria:

- ▪ bilingual in English and the target language (ideally a native speaker and resident of the country of the target language);
- ▪ has ability to mediate between different translators and to understand the points of view of lay and professional translators.

If the coordinator is not a native speaker, a referee (native speaker) must be nominated. The referee cannot be involved in the translation process, and is called upon to arbitrate should irreconcilable views among translators prevent the production of a consensus-based translation.

The tasks of the coordinator include:

- ▪ selecting the translators, assessor and review panel (and referee when necessary);
- ▪ organising and overseeing the translation, including meeting with the translators to produce a consensus-based translation;
- ▪ organising and overseeing the quality assurance of the translation;
- ▪ producing the report of the translation process.

***2. Translation into target language***

Two independent translations into the target language of the original document must be produced.

The two translations may be carried out by two individual translators, by two pairs of translators (one translates and the second of the pair reviews the translation) or by two independent panels of translators (with 3–4 members in each panel). If a translator pair or a panel is used, one person should be identified as lead, and be responsible for liaising with the translation coordinator. The two individuals, pairs or panels may not confer with each other until each has produced their translation.

Translators are selected according to the following criteria:

- ▪ native speaker of the target language;
- ▪ at least one (individual, pair or panel) must be headache or medical expert(s)
- ▪ (ideally, the other is a professional translator or bilingual person, pair or panel skilled in language/linguistics, such as a teacher or journalist; if no such translator is available, then a second headache or medical expert [individual, pair or panel] may be used).

Translators are instructed to:

- ▪ keep translations simple, avoiding technical language, so that the documents can be understood by lay people of average reading ability;
- ▪ make semantic and conceptual translations (rather than literal), so that the meanings of the words and phrases remain as in the original document;
- ▪ keep a record of any parts that they found difficult to translate.

***3. Production of a consensus-based translation***

The coordinator works with the two translators, or the leads of the translation pairs or panels, to reconcile differences between the two translations and produce a consensus-based translation. There are three steps to this process:

- ▪ the translators each send their translations to the coordinator;
- ▪ the coordinator makes an initial comparison of the two translations and highlights and records any parts of them that are substantially different;
- ▪ the coordinator and translators (or leads) meet (or, alternatively, hold a teleconference) to discuss these parts and any other problem areas, agreeing through consensus on one translation.

If the translators cannot reach a consensus on any part, the coordinator, if a native speaker, makes the final decision. If the coordinator is not a native speaker, the referee is called upon to make the final decision.

***4. Quality assessment***

*a) Linguistic review*

One assessor is selected according to the following criteria:

- ▪ a lay person (not medically qualified and not a researcher);
- ▪ a native speaker of the target language (and, ideally, a resident of the relevant country) with good understanding of linguistic factors (such as grammar, readability) but not necessarily bilingual.

The assessor is instructed:

- ▪ that the document is to be understood by lay people of average reading ability;
- ▪ to assess the consensus-based translation for readability, grammatical correctness and cultural suitability;
- ▪ to keep a record of his/her comments and send these to the coordinator.

*b) Target audience review*

A second quality assessment judges suitability for the intended audience. It is carried out by a review panel of six people selected according to the following criteria:

- ▪ affected by headache disorders;
- ▪ native speakers of the target language and not necessarily bilingual.

Each panel member assesses the consensus-based translation individually, without reference to the others, sending comments to the coordinator.

*c) Production of final quality-assured translation*

Minor changes suggested by the assessor or panel members may be implemented by the coordinator (in consultation if necessary with the referee).

When substantial changes are suggested, the coordinator must liaise with the translators, and referee if necessary, in order to agree on an alternative translation. If substantial changes are agreed, the quality of the new translation should be re-assessed by the same processes.

***5. Report of the translation process***

The coordinator should produce a report in English on the translation process, documenting the details (qualifications and experience) of the translators, referee, assessors and review panel members. Furthermore, the report will contain:

- ▪ the original document;
- ▪ the two first translations, the consensus-based translation, any other intermediate versions and the final translation;
- ▪ a record of any substantial difficulties encountered during the translation (difficulties may include problematic words or parts of the document that were difficult to translate, points of disagreement and alternatives, or any aspects on which it was difficult to achieve consensus or that were highlighted during the quality assessment of the translation).

The report is to be sent to LTB (mail@l-t-b.org), addressed to the Company Secretary.

##### Resolving problems

Any problems with or queries about this translation process should be addressed to LTB (mail@l-t-b.org).

#### Translation protocol for technical documents (2nd edition)

This protocol can be separately downloaded (Additional file 29).

These guidelines are for the translation of documents (“technical documents”) produced for the Global Campaign against Headache and aimed at health-care professionals.

Translations of all technical documents should follow these guidelines to ensure a high quality of translation and to be approved by *Lifting The Burden*.

##### Procedure

Translation should follow five steps.

***1. Coordination of the translation***

A translation coordinator, who oversees but does not carry out the translation, is selected according to the following criteria:

- ▪ a headache expert;
- ▪ bilingual in English and the target language (ideally a native speaker and a resident of the country of the target language);
- ▪ has ability to mediate between different translators and to understand the points of view of lay and professional translators.

If the coordinator is not a native speaker, a referee (native speaker) must be nominated. The referee cannot be involved in the translation process, and is called upon to arbitrate should irreconcilable views among translators prevent the production of a consensus-based translation.

The tasks of the coordinator include:

- ▪ selecting the translators and assessors (and referee when necessary);
- ▪ organising and overseeing the translation, including meeting with the translators to produce a consensus-based translation;
- ▪ organising and overseeing the quality assurance of the translation;
- ▪ producing the report of the translation process.

***2. Translation into target language***

Two independent translations into the target language of the original document must be produced.

The two translations may be carried out by two individual translators, by two pairs of translators (one translates and the second of the pair reviews the translation) or by two independent panels of translators (with 3–4 members in each panel). If a translator pair or a panel is used, one person should be identified as lead, and be responsible for liaising with the translation coordinator. The two individuals, pairs or panels may not confer with each other until each has produced their translation.

Translators are selected according to the following criteria:

- ▪ native speakers of the target language;
- ▪ at least one (individual, pair or panel) must be headache expert(s) or primary-care physician(s), according to the intended audience of the document;
- ▪ (ideally, the other is a professional translator or bilingual person, pair or panel skilled in language/linguistics, such as a teacher or journalist; if no such translator is available, then a second headache expert or primary-care physician [individual, pair or panel] may be used).

Translators are instructed to:

- ▪ keep translations professional, using technical language;
- ▪ make semantic and conceptual translations (rather than literal), so that the meanings of the words and phrases remain as in the original document;
- ▪ avoid invention (adding their own ideas to the text);
- ▪ keep a record of any parts that they found difficult to translate.

***3. Production of a consensus-based translation***

The coordinator works with the two translators, or the leads of the translation pairs or panels, to reconcile differences between the two translations and produce a consensus-based translation. There are three steps to this process:

- ▪ the translators each send their translations to the coordinator;
- ▪ the coordinator makes an initial comparison of the two translations and highlights and records any parts of them that are substantially different;
- ▪ the coordinator and translators (or leads) meet (or, alternatively, hold a teleconference) to discuss these parts and any other problem areas, agreeing through consensus on one translation.

If the translators cannot reach a consensus on any part, the coordinator, if a native speaker, makes the final decision. If the coordinator is not a native speaker, the referee is called upon to make the final decision.

***4. Quality assessment***

Three assessors are selected according to the following criteria:

- ▪ either headache experts or primary-care physicians, according to the intended audience of the document;
- ▪ native speakers of the target language (and, ideally, a resident of the relevant country) with good understanding of linguistic factors (such as grammar, readability) but not necessarily bilingual.

The assessors are instructed:

- ▪ that the document is to be utilized by health-care professionals (specified, when appropriate);
- ▪ to assess the consensus-based translation for readability, grammatical correctness, medical correctness and cultural suitability;
- ▪ to keep records of their comments and send these to the coordinator.

Each assessor reviews the consensus-based translation individually, without reference to the others, sending comments to the coordinator.

Minor changes suggested by the assessors may be implemented by the coordinator (in consultation if necessary with the referee).

When substantial changes are suggested, the coordinator must liaise with the translators, and referee if necessary, in order to agree on an alternative translation. If substantial changes are agreed, the quality of the new translation should be re-assessed by the same processes.

***5. Report of the translation process***

The coordinator should produce a report in English on the translation process, documenting the details (qualifications and experience) of the translators, referee and assessors. Furthermore, the report will contain:

- ▪ the original document;
- ▪ the two first translations, the consensus-based translation, any other intermediate versions and the final translation;
- ▪ a record of any substantial difficulties encountered during the translation (difficulties may include problematic words or parts of the document that were difficult to translate, points of disagreement and alternatives, or any aspects on which it was difficult to achieve consensus or that were highlighted during the quality assessment of the translation).

The report is to be sent to LTB (mail@l-t-b.org), addressed to the Company Secretary.

##### Resolving problems

Any problems with or queries about this translation process should be addressed to LTB (mail@l-t-b.org).

#### Translation protocol for hybrid documents (2nd edition)

This protocol can be separately downloaded (Additional file 30).

These guidelines are for the translation of documents (“hybrid documents”) produced for the Global Campaign against Headache and aimed at people with headache, but to be used in support either of clinical practice or of research (such as questionnaires, diaries, survey instruments).

Translations of all hybrid documents should follow these guidelines to ensure a high quality of translation and to be approved by *Lifting The Burden*.

##### Procedure

Translation should follow six steps.

***1. Coordination of the translation***

A translation coordinator, who oversees but does not carry out the translation, is selected according to the following criteria:

- ▪ has technical knowledge (*ie*, understands the concepts underlying the questions or instrument being translated);
- ▪ bilingual in English and the target language (ideally a native speaker and a resident of the country of the target language);
- ▪ has ability to mediate between different translators and to understand the points of view of lay and professional translators.

If the coordinator is not a native speaker, a referee (native speaker) must be nominated. The referee cannot be involved in the translation process, and is called upon to arbitrate should irreconcilable views among translators prevent the production of a consensus-based translation.

The tasks of the coordinator include:

- ▪ selecting the forward- and back-translators, assessor and review panel (and referee when necessary);
- ▪ liaising when necessary with the document author;
- ▪ organising and overseeing the forward- and back-translations, including meeting with the translators first to produce a consensus-based forward-translation and again (when necessary) to resolve discrepancies discovered during back-translation;
- ▪ organising and overseeing the quality assurance of the translation;
- ▪ producing the report of the translation process.

***2. Translation into target language***

Two independent forward-translations into the target language of the original document must be produced.

The two translations may be carried out by two individual translators, by two pairs of translators (one translates and the second of the pair reviews the translation) or by two independent panels of translators (with 3–4 members in each panel). If a translator pair or a panel is used, one person should be identified as lead, and be responsible for liaising with the translation coordinator. The two individuals, pairs or panels may not confer with each other until each has produced their translation.

Translators are selected according to the following criteria:

- ▪ native speaker of the target language;
- ▪ at least one (individual, pair or panel) must be headache or medical expert(s);
- ▪ (ideally, the other is a professional translator or bilingual person, pair or panel skilled in language/linguistics, such as a teacher or journalist; if no such translator is available, then a second headache or medical expert [individual, pair or panel] may be used).

Translators are provided by the coordinator with an explanation of the purpose and concepts underlying the elements of the document (obtained, when necessary, from the document author).

Translators are instructed to:

- ▪ keep translations simple, avoiding technical language, so that the documents can be understood by lay people of average reading ability;
- ▪ make semantic and conceptual translations (rather than literal), so that the meanings of the words and phrases remain as in the original document;
- ▪ keep a record of any parts that they found difficult to translate.

***3. Production of a consensus-based translation***

The coordinator works with the two translators, or the leads of the translation pairs or panels, to reconcile differences between the two translations and produce a consensus-based translation. There are three steps to this process:

- ▪ the translators each send their translations to the coordinator;
- ▪ the coordinator makes an initial comparison of the two translations and highlights and records any parts of them that are substantially different;
- ▪ the coordinator and translators (or leads) meet (or, alternatively, hold a teleconference) to discuss these parts and any other problem areas, agreeing through consensus on one forward translation.

If the translators cannot reach a consensus on any part, the coordinator, if a native speaker, makes the final decision. If the coordinator is not a native speaker, the referee is called upon to make the final decision.

***4. Back-translation***

One back-translation of the consensus-based forward translation is carried out by one translator selected according to the following criteria:

- ▪ a native speaker of English;
- ▪ either a headache or medical expert, or a professional or bilingual lay translator skilled in language/linguistic issues.

The back-translation is sent to the coordinator to forward to the original author with a request to compare the original and back-translated versions and assess their conceptual equivalence. If the author believes conceptual equivalence is not maintained, he or she should be asked to explain the reasons to the coordinator.

Following this conceptual comparison, minor amendments may be implemented by the coordinator (in consultation with the referee when appropriate). When substantial discrepancies have been highlighted, the coordinator calls a second meeting (or teleconference) with the forward-translators and back-translator to locate their causes and eliminate them by making changes either to the consensus-based forward-translation or to the back-translation as appropriate.

This process produces the back-checked consensus-based translation.

***5. Quality assessment***

*a) Linguistic review*

One assessor is selected according to the following criteria:

- ▪ a lay person (not medically qualified and not a researcher);
- ▪ a native speaker of the target language (and, ideally, a resident of the relevant country) with good understanding of linguistic factors (such as grammar, readability) but not necessarily bilingual.

The assessor is instructed:

- ▪ that the document is to be understood by lay people of average reading ability;
- ▪ to assess the back-checked consensus-based translation for readability, grammatical correctness and cultural suitability;
- ▪ to keep a record of his/her comments and send these to the coordinator.

*b) Target audience review*

A second quality assessment judges suitability for the intended audience. A review panel of six people are selected according to the following criteria:

- ▪ affected by headache disorders;
- ▪ native speakers of the target language and not necessarily bilingual.

Each panel member assesses the back-checked consensus-based translation individually, without reference to the others, sending comments to the coordinator.

*c) Production of final quality-assured translation*

Minor changes suggested by the assessor or panel members may be implemented by the coordinator (in consultation if necessary with the referee).

When substantial changes are suggested, the coordinator must liaise with the forward-translators, and referee if necessary, to agree on an alternative translation. If substantial changes are agreed, the back-translation process should be repeated and, subsequently, the quality of the new translation should be re-assessed.

***6. Report of the translation process***

The coordinator should produce a report in English on the translation process, documenting the details (qualifications and experience) of the translators, referee, assessors and review panel members. Furthermore, the report will contain:

- ▪ the original document;
- ▪ the two forward-translations, the consensus-based translation, the back-translation, the back-checked consensus-based translation, any other intermediate versions and the final translation;
- ▪ a record of any substantial difficulties encountered during the translation (difficulties may include problematic words or parts of the document that were difficult to translate, points of disagreement and alternatives, or any aspects on which it was difficult to achieve consensus or that were highlighted during the quality assessment of the translation).

The report is to be sent to LTB (mail@l-t-b.org), addressed to the Company Secretary.

##### Resolving problems

Any problems with or queries about this translation process should be addressed to LTB (mail@l-t-b.org).

#### Commonalities between the three translation protocols

All protocols aim for semantic and conceptual equivalence: literal translations often produce wording that is not acceptable, is unnatural or has wrong meaning in the target language. Lay and hybrid translation protocols avoid technical jargon, while recognising that medical terminology must nonetheless be accurate.

All protocols prescribe two independent forward translations with reconciliation to produce a consensus version. Even when a translator appears to have all the requisite skills, a single translation is unreliable: a non-expert in the field may misunderstand, while experts tend to “invent” – introducing their own ideas to “improve” the original. Multiple forward translations are a guard against biased translation and misinterpretations, while helping to highlight areas that are difficult to translate or have not been translated well.

All protocols rely on a coordinator, and specify the necessary skills of the translators. The coordinator, bilingual but a native speaker of the *target* language, selects the translators and organises and oversees (but does not carry out) the translations. Ideally the coordinator should live in the country of the target language in order to be wholly familiar with its culture, but this raises some issues: what, for example, is the native country for Spanish? The obvious answer is neither a complete answer nor necessarily correct: cultural (and to some extent linguistic) differences between Spain and Spanish-speaking countries in Latin America are not negligible. These issues may influence the selection of coordinator and, probably more importantly, of the translators. For Global Campaign translations, support in these selections can be given by LTB.

The forward translations are both best made by translators who are translating into their native language [15]. But, further, they must speak this target language correctly and with linguistic competence, which is not always the case for native speakers and cannot be assumed. The forward translators should also have an understanding of the *culture* in which the target language is used, and again, ideally, should therefore be living in the country of the target language. Although emphasis in good translating is often put on linguistic skills, translators also need some knowledge and understanding of the topic area or content of the material [22]. This might, according to the nature of what is being translated, be from the perspective of health-care professional or person with headache, but all three LTB protocols require that at least one forward translator is a headache or medical expert.

The coordinator decides whether individual or panel translations are more suitable for the culture and language. Individual translations require fewer translators but, where skilled and otherwise qualified individual translators are not available, a group of translators meeting together as a panel can contribute a wider range of competencies to the translation process. A panel translation is considered to be one translation: the two forward translations should be generated independently.

From the two forward translations, one consensus version is produced in a reconciliation process involving direct collaboration between the coordinator and the translators. This step resolves discrepancies between the two forward translations [23] and allows – in fact, requires – comparison of the translated version with the original. The coordinator’s role here is to negotiate agreement between the translators, having the final say when the two translators cannot agree.

All protocols require quality evaluation, conducted with representatives of the respective target audiences (either people with headache or health-care professionals). These, too, should be native speakers of the target language, but not necessarily bilingual (in English). This additional process ensures that translations make sense, have meaning and are otherwise acceptable to the target audience. Specifically it allows translations to be amended, when necessary, to be more “user-friendly”.

Finally, all protocols require a full report of these processes, including all translated versions (intermediate and final) and listings of any encountered translation difficulties. This report is sent to LTB. Reporting back in this way to LTB helps to ensure that the translation procedures have been followed, and also that there are not several translations into one target language. It also allows LTB to make already translated documents widely available.

#### Differences between the three translation protocols

Important differences between the three protocols adapt the recommended procedures according to the type of document being translated. They make translation less onerous whenever this is possible without compromising quality.

First, the criteria for coordinators differ. For hybrid translations, the coordinator must have technical knowledge – *ie*, the ability to understand the concepts underlying the instrument to be translated. Hybrid documents are often questionnaires, and accurate translation of items requires capture of the *conceptual* rather than the *literal* meaning. In contrast, the coordinator for technical translations must be a headache expert, since the target audience for these is medical and health professionals. A headache expert is more likely to know the correct terminology for this target audience, which is of importance when coordinating the production of a consensus version of the translation.

Second, the protocol for hybrid documents requires back translation as an additional step. These documents may be used for research purposes and cross-cultural comparisons, and this further process increases the likelihood of conceptual equivalence, whereas the approach to lay and technical documents is more pragmatic (*ie*, two forward translations only). This decision reflected the view that more emphasis should rest on quality evaluation by the more-clearly defined target audiences for both lay and technical documents.

Consequently, a third difference lies in how translation quality is evaluated. For hybrid and lay documents, evaluation includes a linguistic review in addition to testing by the target audience. This is conducted by a person with a good understanding of *language*, who need not be a person with headache or a health-care professional. This process is important to exclude jargon, and to make hybrid and lay documents understandable at least to those of average reading ability.

#### Updates to the protocols

The three protocols were originally published in 2007 [19]. In this second edition, the changes are minor: there are new support details, but no material changes have been necessary in the methods prescribed in each.

Further updates will be made when circumstances require them. Meanwhile, these second-edition protocols should be used from now on for all Campaign materials, whether related to clinical management, policy or research.

### Resources for translation of *Lifting The Burden* documents

These three protocols serve several purposes, including standardisation of translations for Global Campaign materials. They set out clear steps for the coordinator, translators and evaluators. Their success in achieving their purpose will depend on their being carefully followed.

Those proposing to undertake translation into any language of any Global Campaign product should do so in consultation with LTB. It may be that an accepted translation already exists. By this token, all translations completed in accordance with the appropriate protocol, along with the translation report, should be lodged with LTB (addressed to the Company Secretary [mail@l-t-b.org]). Any problems or queries may be addressed to LTB (mail@l-t-b.org).

The Global Campaign depends heavily on volunteers in all its endeavours. Clearly, the main resource-requirement in translating is for volunteers able to coordinate, perform or evaluate the translation. Hybrid translations call for a minimum of 11 people, lay translations a minimum of 10 people, and technical documents a minimum of five. More are required if panels are used to produce any of the translations.

Although these may seem large numbers of people, other translation protocols (*eg*, EuroQol [21], ISOQOL [20]) impose similar or greater demands. The IQOLA project used six translators, a national principal investigator (equivalent to our coordinator) and pilot testing with up to 50 respondents [20]. The recommendations of the ISPOR Task Force for Translation and Cultural Adaptation call for 9–12 people [15]. While the translation protocol for hybrid documents is more elaborate because of the additional back translation, and requires more translators, these documents, usually questionnaires, tend to be relatively short. Hence, back translation is not too onerous. Longer lay and technical documents can, of course, often be divided into small sections to reduce the burden of translation on any one translator.

## Additional files

Additional file 1: Guides to diagnosis: Headache as a presenting complaint. (PDF 166 kb)

Additional file 2: Guides to diagnosis: Typical features of the headache disorders relevant to primary care. (PDF 237 kb)

Additional file 3: Guides to diagnosis: Diagnosis of headache disorders. (PDF 218 kb)

Additional file 4: Guides to management: General aspects of headache management. (PDF 187 kb)

Additional file 5: Guides to management: Advice to patients. (PDF 190 kb)

Additional file 6: Guides to management: Management of migraine. (PDF 376 kb)

Additional file 7: Guides to management: Management of migraine; Acute or symptomatic management of episodic migraine. (PDF 376 kb)

Additional file 8: Guides to management: Management of migraine; Prophylactic management of episodic migraine. (PDF 192 kb)

Additional file 9: Guides to management: Management of migraine; Management of chronic migraine. (PDF 136 kb)

Additional file 10: Guides to management: Management of tension-type headache. (PDF 217 kb)

Additional file 11: Guides to management: Management of cluster headache. (PDF 207 kb)

Additional file 12: Guides to management: Management of medication-overuse headache. (PDF 190 kb)

Additional file 13: Guides to management: Management of trigeminal neuralgia and persistent idiopathic facial pain. (PDF 185 kb)

Additional file 14: Guide to referral: Headache management in primary care: when to refer. (PDF 145 kb)

Additional file 15: Instruments and other materials to aid diagnosis and management of headache disorders in primary care: Diagnostic criteria for headache disorders in primary care: The International Classification of Headache Disorders, 3rd edition (ICHD-3) – abbreviated form. (PDF 258 kb)

Additional file 16: Instruments and other materials to aid diagnosis and management of headache disorders in primary care: Diagnostic headache diary. (PDF 193 kb)

Additional file 17: Instruments and other materials to aid diagnosis and management of headache disorders in primary care: Headache calendar for follow-up. (PDF 155 kb)

Additional file 18: Instruments and other materials to aid diagnosis and management of headache disorders in primary care: The HALT-90 Index. (PDF 287 kb)

Additional file 19: Instruments and other materials to aid diagnosis and management of headache disorders in primary care: The HALT-30 Index. (PDF 283 kb)

Additional file 20: Instruments and other materials to aid diagnosis and management of headache disorders in primary care: The HURT questionnaire. (PDF 290 kb)

Additional file 21: Patient information leaflets to aid headache management in primary care (2nd edition): Migraine. (PDF 171 kb)

Additional file 22: Patient information leaflets to aid headache management in primary care (2nd edition): Tension-type headache. (PDF 162 kb)

Additional file 23: Patient information leaflets to aid headache management in primary care (2nd edition): Cluster headache. (PDF 165 kb)

Additional file 24: Patient information leaflets to aid headache management in primary care (2nd edition): Medication-overuse headache. (PDF 166 kb)

Additional file 25: Patient information leaflets to aid headache management in primary care (2nd edition): Female hormones and headache. (PDF 160 kb)

Additional file 26: Patient information leaflets to aid headache management in primary care (2nd edition): Trigeminal neuralgia. (PDF 120 kb)

Additional file 27: Patient information leaflets to aid headache management in primary care (2nd edition): Persistent idiopathic facial pain. (PDF 251 kb)

Additional file 28: Translation, and the preservation of original meaning, of materials developed to improve headache management: Translation protocol for lay documents (2nd edition). (PDF 146 kb)

Additional file 29: Translation, and the preservation of original meaning, of materials developed to improve headache management: Translation protocol for technical documents (2nd edition). (PDF 145 kb)

Additional file 30: Translation, and the preservation of original meaning, of materials developed to improve headache management: Translation protocol for hybrid documents (2nd edition). (PDF 177 kb)

## Footnotes

1 ICHD terminology aligns with that of the International Classification of Diseases (ICD).
