# Supplementary material for: Aids to management of headache disorders in primary care (2nd edition): on behalf of the European Headache Federation and Lifting The Burden: the Global Campaign against Headache
Source: J Headache Pain. 2019 May 21;20(1):57. doi: 10.1186/s10194-018-0899-2 (PMC6734476; doi:10.1186/s10194-018-0899-2)
Supplement: Supplementary file 1 — Guides to diagnosis: Headache as a presenting complaint. (PDF 166 kb) [file 10194_2018_899_MOESM1_ESM.pdf]

in collaboration with

Lifting The Burden

European principles of management of headache disorders in primary care (2nd edition)

1. Headache as a presenting complaint

Most people have occasional headache. This is a **symptom**, which many people regard as "normal". Headache becomes a problem at some time in the lives of about 40% of adults and lesser but still substantial proportions of children and adolescents. These people have a **headache disorder**.

Table 1. The headache disorders of particular importance in primary care

**Migraine**	• usually episodic, occurring in 15-25% of the general population, in women more than men in a ratio of up to 3:1;<br>• a chronic type is recognised, with headache occurring on more days than not
**Tension-type headache**	• usually episodic, affecting most people from time to time but, in at least 10%, recurring frequently;<br>• in up to 3% of adults and some children it is chronic, occurring on more days than not
**Cluster headache**	• extremely intense and frequently recurring but short-lasting headache attacks, affecting up to 3 in 1,000 men and up to 1 in 2,000 women
**Medication-overuse headache**	• a secondary headache, but occurring only as a complication of a pre-existing headache disorder, usually migraine or tension-type headache, present on most days (≥15 days/month) and affecting 1-2% of adults, women more than men, and about 0.5% of children and adolescents

The International Classification of Headache Disorders (ICHD) [4] (available in abbreviated form as Supplementary materials #15) recognises over 200 headache disorders, and divides them into three groups.

- **Primary headache disorders** include migraine, tension-type headache (TTH) and cluster headache, all of which are important in primary care (Table 1).
- **Secondary headache disorders** have another causative disorder underlying them; therefore the headache occurs in close temporal relation to the other disorder, and/or worsens or improves in parallel with worsening or improvement of that disorder. These associations are keys to their diagnosis. Secondary headache disorders include medication-overuse headache (MOH), also important in primary care (Table 1).

- **Painful cranial neuropathies and other facial pains** include two disorders, trigeminal neuralgia and persistent idiopathic facial pain, that need to be recognised in primary care.

A patient may have **more than one of these disorders** concomitantly.

Which headaches should be managed where?

Four headache disorders are of particular importance in primary care (Table 1). All have a neurobiological basis. They are variably painful and disabling, but all may cause lost productivity and impair quality of life. Collectively they are the second highest cause of disability worldwide [5], and therefore very costly.

- **Migraine, TTH and MOH** can and should, almost always, be managed well in primary care.
    - Specific advice on each of these is given in Supplementary materials #6, Supplementary materials #10 and Supplementary materials #12.
- The exception is **chronic migraine**. This uncommon type should be recognised in primary care, but it is difficult to treat and likely to require specialist management.
    - Specific advice on this is in Supplementary materials #9.
- **Cluster headache** should be diagnosed in primary care because it is easily recognisable, but referred for specialist management.
    - Specific advice on this is in Supplementary materials #11.
- Among painful cranial neuropathies and other facial pains are **trigeminal neuralgia** and **persistent idiopathic facial pain**. These should be recognised when present but require specialist management.
    - Specific advice on each of these is in Supplementary materials #13.
- Any headache **not responding satisfactorily** to management in primary care should also be referred for specialist management.
- Of the large number of other secondary headache disorders, **some are serious**. Overall these account for <1% of patients presenting with headache, but they **must be recognised**.
    - Advice on these is provided in Supplementary materials #3.

More general advice on indications for referral to specialist management is set out in Supplementary materials #14.
