# Supplementary material for: Aids to management of headache disorders in primary care (2nd edition): on behalf of the European Headache Federation and Lifting The Burden: the Global Campaign against Headache
Source: J Headache Pain. 2019 May 21;20(1):57. doi: 10.1186/s10194-018-0899-2 (PMC6734476; doi:10.1186/s10194-018-0899-2)
Supplement: Supplementary file 2 — Guides to diagnosis: Typical features of the headache disorders relevant to primary care. (PDF 237 kb) [file 10194_2018_899_MOESM2_ESM.pdf]

in collaboration with

Lifting The Burden

European principles of management of headache disorders in primary care (2nd edition)

2. Typical features of the headache disorders relevant to primary care

The **distinguishing features** of the important primary headache disorders are summarised in Table 1.

Migraine

Migraine is typically a **moderate-to-severe headache** accompanied by **nausea, vomiting** and **sensitivity to light and/or noise**. It is more prevalent among women than among men.

Migraine is usually episodic, occurring in attacks lasting hours to a few days. The **two principal types** are migraine without aura and the less common migraine with aura. One patient may have both types. There is also an uncommon chronic type.

Migraine without aura

Adults with this disorder describe:

- recurrent episodic **moderate or severe headaches** which, *typically but not always*:
  - are **unilateral** and/or **pulsating**;
  - last (when untreated) from 4 hours to 3 days;
  - are **associated with**:
    - nausea and/or vomiting;
    - photophobia, phonophobia and sometimes osmophobia;
  - are aggravated by routine physical activity, and **disabling**;
  - and during which they limit their activity and prefer **dark and quiet**;
- **freedom** from these symptoms **between attacks**.

In **children:**

- attacks may be shorter-lasting;
- headache is more often bilateral and less often pulsating;
- gastrointestinal disturbance is often more prominent.

Table 1. Summary of features distinguishing the important primary headache disorders (NB: two or more of these disorders may occur concomitantly)

	Migraine	Tension type headache (TTH)	Cluster headache (CH)
**Temporal pattern**	**Episodic migraine:**<br>recurrent attack-like episodes, lasting from 4 hours to 3 days;<br>frequency often 1-2/month but variable from 1/year to 2/week or more;<br>freedom from symptoms between attacks<br>**Chronic migraine:**<br>episodicity lost: headache on ≥15 days/month, having migrainous features on ≥8 days/month	**Frequent episodic TTH:**<br>recurrent attack-like episodes lasting hours to a few days;<br>1-14 days affected per month;<br>freedom from symptoms between attacks<br>**Chronic TTH:**<br>≥15 days affected per month (often daily and unremitting)	**Episodic CH:**<br>frequent (typically ≥1 daily) short-lasting attacks (15-180 minutes):<br>• recurring in bouts, usually once or sometimes twice a year, which are typically of 6-12 weeks' duration;<br>• then remitting for ≥3 months<br>**Chronic CH:**<br>similar, but without such remissions between bouts
**Typical headache characteristics**	often unilateral;<br>often pulsating	can be unilateral but more often generalised; may spread to the neck;<br>typically described as pressure or tightness	strictly unilateral (although side-shifts occur occasionally), around the eye or over the temple
**Headache intensity**	typically moderate to severe	typically mild to moderate	extremely severe
**Associated symptoms**	aura (in a minority of attacks);<br>often nausea and/or vomiting;<br>often photo- and/or phonophobia	**Frequent episodic TTH:**<br>none typical; mild photophobia or phonophobia may occur<br>**Chronic TTH:**<br>sometimes mild nausea, but not vomiting	strictly ipsilateral autonomic features:<br>• any or all of red and/or watering eye, running or blocked nostril, ptosis
**Reactive behaviour**	avoidance of physical activity (maybe bed rest); preference for dark and quiet	none specific	marked agitation: cannot lie still during attacks

Migraine with aura

This type affects about one third of people with migraine, although only a minority of these experience aura symptoms with every attack. It is characterised by:

- **aura** preceding or less commonly accompanying headache and consisting of **one or more neurological symptoms** (see Table 2);
- **headache** that is similar to migraine without aura, or may be rather featureless.

Typical aura without headache may occur in patients with a past history of migraine with aura.

Table 2. Symptoms of aura (developing gradually over ≥5 minutes and usually resolving within 60 minutes)

**Typical**	• **visual symptoms** (occurring in >90% of auras): usually a slowly-enlarging scintillating scotoma (patients may draw a jagged crescent if asked); *and/or*<br>• **unilateral paraesthesiae** and/or numbness of hand, arm and/or face
**Less usual**	• brainstem symptoms (*eg*, vertigo, tinnitus, diplopia, ataxia);<br>• speech and/or language disturbances
**Rare**	• motor weakness

Chronic migraine

This highly disabling migraine type develops, in a small minority of patients, from episodic migraine. Over time, attacks become more frequent, with **loss of clear periodicity**. Simultaneously, the specific characteristics of migraine become less pronounced.

Chronic migraine is **not simply more frequent migraine**. It is essentially characterised by:

- **headache** occurring on **≥15 days/month** for at least 3 months which:
- on **≥8 days/month** meets diagnostic criteria for **migraine** (or responds to migraine-specific drug treatment);

and often **complicated by**:

- depression and/or anxiety;
- low back and/or neck pain;
- medication overuse.

Transformation of episodic migraine to a chronic headache disorder is very often **causally associated with medication overuse**:

- the correct diagnosis is then **medication-overuse headache** (MOH);
- chronic migraine and MOH are **not mutually exclusive** but, when medication is being overused, it may be that only MOH and not chronic migraine is present.

Tension-type headache (TTH)

This disorder is typically a **mild-to-moderate headache** of highly variable frequency and duration, **without associated symptoms** or the specific features of migraine. It tends to be more common in women than in men.

It has **three types**. Infrequent episodic TTH, occurring less than once a month, is not medically important. The others are frequent episodic TTH and chronic TTH.

Frequent episodic tension-type headache

- occurs in attack-like episodes on **1-14 days/month**, each lasting hours to a few days;
- can be unilateral but is more often generalised;
- is typically described as **pressure or tightness** like a vice or tight band around the head, often spreading to the neck;
- lacks the associated symptom complex of migraine.

Chronic tension-type headache

This type has features similar to those of frequent episodic TTH but:

- occurs on **≥15 days/month for >3 months**, and may be daily and unremitting;
- may be associated with mild nausea.

Cluster headache

This disorder is characterised by frequently recurring, localised, **short-lasting** but **extremely severe headache** accompanied by a set of very recognisable **autonomic symptoms**. It affects men three times as commonly as women.

It **should never be missed**. It demands accelerated specialist referral, investigation and treatment.

Cluster headache occurs in attacks, which *very typically*:

- are characterised by headache of **excruciating intensity**, which is:
  - strictly **unilateral and localised** around the eye or over the temple;
  - accompanied by highly characteristic and strictly ipsilateral **autonomic features**, including any or all of:

        - red and watering eye;
        - running or blocked nostril;
        - ptosis;
    - associated with marked **agitation** (the patient, unable to stay in bed, paces the room, even going outdoors);
- occur **once or more daily**, very often at night (causing awakening);
- last **15-180 minutes** (commonly 30-60).

Cluster headache has **two subtypes**, episodic and (less common) chronic.

Episodic cluster headache

- occurs in **bouts** (clusters) of recurring attacks, typically **once or twice a year**, which:
    - are of 6-12 weeks' duration (but may be longer);
    - then remit until the next cluster, at least 3 months later.

Chronic cluster headache

- persists, still as recurring attacks but **without remissions**, or with remissions of <3 months;
- may develop from and/or revert to episodic cluster headache.

Medication-overuse headache (MOH)

This is one of the syndromes characterised by **headache occurring on ≥15 days/month**. It is often daily, but variable in site, intensity and character. It greatly impairs quality of life. It is more common in women.

Medication-overuse headache:

- occurs **daily or near-daily** (by definition on ≥15 days/month);
- is present – and often at its worst – early in the morning;
- is **causally associated** with regular use, over >3 months, of:
    - non-opioid analgesics on ≥15 days/month, and/or
    - opioids, ergots or triptans, or any combination of these, on ≥10 days/month.

MOH is an **aggravation of a prior headache** (usually migraine or tension-type headache) by chronic overuse of medication taken to treat headache or other pain. A history can usually be elicited of increasingly frequent and difficult-to-treat headache episodes, with increasing medication use, over months to many years.

All acute headache medications may have this effect. Frequency, regularity and duration of intake are important determinants of risk.

MOH tends to worsen initially when attempts are made to reduce consumption of the overused medication(s), but in most cases improves within 2 months after overuse is stopped.

Important causes of facial pain

Many causes of facial pain may bring patients to GPs. Two in particular, although not common, require recognition.

Trigeminal neuralgia (TN)

This disorder presents as recurrent, unilateral, **brief but severe, electric-shock-like pains** in the distribution of the trigeminal nerve, abrupt in onset and termination and often triggered by innocuous stimuli.

- Trigeminal neuralgia (TN) affects women twice as commonly as men, and mostly those above 50 years of age (but may occur in younger people). It has no other known risk factors.
- It is often associated with neurovascular compression of the trigeminal nerve close to its point of entry to the brainstem (**classical trigeminal neuralgia**).
- TN is one of the **most painful disorders**, demanding **accelerated specialist referral**, investigation and treatment.
- **MRI** of the brain (including brainstem) is essential.
    - This may demonstrate neurovascular compression, but is required in any case to exclude secondary causes that give rise to pains indistinguishable from classical TN. These occur more often in younger people.

Classical trigeminal neuralgia:

- occurs in **bouts of repeated**, stabbing or **electric-shock-like pains** in the distribution of one or more divisions of the trigeminal nerve (usually the 2nd and/or 3rd), which are:
    - **excruciating**;
    - of **sudden onset**;
    - highly characteristically **triggered** by sensory stimuli to the affected side of the face (touching, washing, applying make-up) or by talking, eating, chewing, drinking or smoking;
    - **short-lasting** (from less than a second up to two minutes);
    - **strictly unilateral,** and not switching side between bouts;
    - often **serial**, with up to hundreds of pain paroxysms during one day;
- may also feature a **constant aching pain** between attacks, in the affected area, of moderate intensity.

Bouts may remit completely for months or years in an unpredictable pattern. Otherwise, treatment may require surgical decompression.

Secondary trigeminal neuralgia

- has characteristics similar to classical trigeminal neuralgia, but is secondary to another disorder (usually cerebellopontine angle tumour, AV-malformation or multiple sclerosis).

Persistent idiopathic facial pain (PIFP)

Previously termed "atypical facial pain", this disorder presents as variable but persistent, **poorly localized facial and/or oral pain**. It is more common in women.

Persistent idiopathic facial pain (PIFP):

- is **dull**, aching or nagging;
- **recurs daily** for >2 hours and **persists** over >3 months;
- is **unassociated** with neurological deficit;
- is aggravated by **stress.**

PIFP is associated with high levels of **psychiatric comorbidity** and psychosocial disability, and difficult to manage. It usually requires **specialist referral**. However:

- patients are often referred for exclusion of sinus and dental problems, then returned untreated to primary care;
- referral to a specialist clinic with a pain management programme is preferable.

Temporomandibular disorder (TMD) is in the differential diagnosis of PIFP. This is itself a very complex problem:

- the pain associated with TMD is usually most prominent in the pre-auricular areas of the face, masseter muscles and/or temporal regions;
- there is significant overlap between TMD and tension-type headache and jaw, dental and bite disorders.
