# Supplementary material for: Aids to management of headache disorders in primary care (2nd edition): on behalf of the European Headache Federation and Lifting The Burden: the Global Campaign against Headache
Source: J Headache Pain. 2019 May 21;20(1):57. doi: 10.1186/s10194-018-0899-2 (PMC6734476; doi:10.1186/s10194-018-0899-2)
Supplement: Supplementary file 3 — Guides to diagnosis: Diagnosis of headache disorders. (PDF 218 kb) [file 10194_2018_899_MOESM3_ESM.pdf]

in collaboration with

Lifting The Burden

European principles of management of headache disorders in primary care (2nd edition)

3. Diagnosis of headache disorders

The universally accepted basis for the diagnosis of any headache is the International Classification of Headache Disorders, an abbreviated version of which is available as Supplementary materials #15. In all health-care settings, diagnostic practice should employ ICHD terminology[1].

Differential diagnosis of the headache disorders relevant to primary care

Diagnosis of episodic migraine or episodic tension-type headache requires multiple attacks; neither diagnosis should be made after a first attack without exclusion of other disorders.

- Each of the primary headaches is in the differential diagnosis of each of the others.
- Medication-overuse headache is in the differential diagnosis of chronic migraine or chronic tension-type headache.
- The distinguishing features of these are described in Supplementary materials #2.
- Otherwise, the differential diagnosis potentially includes **a small number of serious secondary headaches that are important to recognise** (see *Warning features in the history or on examination*, below).

Taking a diagnostic history

The **history is all-important** in the diagnosis of the primary headache disorders and of medication-overuse headache. There are no useful diagnostic tests.

Table 1 indicates diagnostic questions to elicit any that may be present of the distinguishing features (see Supplementary materials #2).

---

[1] ICHD terminology aligns with that of the International Classification of Diseases (ICD).

Table 1. Diagnostic questions to ask in the history

**How many different headaches types does the patient have?** A separate history is needed for each.	
**Time questions**	• Why consulting now?<br>• How recent in onset?<br>• How frequent, and what temporal pattern (episodic or daily and/or unremitting)?<br>• How long do headache episodes last?
**Character questions**	• Manner and speed of headache onset (abrupt, progressive over minutes, hours, days or longer)?<br>• Intensity of pain?<br>• Nature and quality of pain?<br>• Site and spread of pain?<br>• Associated symptoms?
**Cause questions**	• Predisposing and/or trigger factors?<br>• Aggravating and/or relieving factors?<br>• Family history of similar headache?
**Response questions**	• What does the patient do during the headache?<br>• How much is activity limited or prevented?<br>• What medications are used, and how frequently?
**State of health between attacks**	• Completely well, or residual symptoms?

Diagnostic diary

A diary kept over a few weeks can be a very helpful diagnostic aid, clarifying the pattern and frequency of headaches and associated symptoms as well as medication use or overuse. An example is available as Supplementary materials #16.

Warning features in the history

The history should also elicit any warning features of a serious secondary headache disorder:

- **any new headache**, or a significant change in headache characteristics, should provoke a new diagnostic enquiry;
- **very frequent headache** should always lead to detailed enquiry into medication use, since overuse is a likely cause;
- in addition, there are a number of **specific warning features** ("red flags") that may be elicited (Table 2).

Table 2. Specific warning features ("red flags") in the history

Warning feature	What to beware of
Thunderclap headache (intense headache with "explosive" or abrupt onset)	Subarachnoid haemorrhage
Headache with atypical aura (duration >1 hour, or including motor weakness)	TIA or stroke
Aura without headache in the absence of a prior history of migraine with aura	
Aura occurring for the first time in a patient during use of combined hormonal contraceptives	Risk of stroke (requires discontinuation)
New headache within 3 months of head trauma	Subdural haematoma
Progressive headache, worsening over weeks or longer	Intracranial space-occupying lesion
Headache aggravated by postures or manoeuvres that raise intracranial pressure	
Headache brought on by coughing, exercise or sexual activity	
Mild-to-moderate progressive or recurrent headache with irritability, dizziness (light-headedness), nausea and/or tiredness and confusion	Carbon monoxide poisoning
Headache associated with unexplained focal neurological symptoms or with epileptic seizures	Suggests secondary headache
Headache associated with change in memory or personality	
Headache associated with weight-loss	
New headache in a patient older than 50 years	Temporal arteritis or intracranial tumour
New headache in a patient with a history of cancer or immunodeficiency (including HIV infection)	Likely to be secondary headache
New headache in a patient with a history of polymyalgia rheumatica	Temporal (giant cell) arteritis
New headache in a patient with a family history of glaucoma	Glaucoma

Physical examination of headache patients

Migraine, tension-type headache, cluster headache and medication-overuse headache are diagnosed **solely on history**. Signs are present in cluster headache patients when seen during attacks (red and/or watering eye, running or blocked nostril and/or ptosis ipsilateral to the pain).

- **Blood pressure** measurement in all cases is good practice.
- Physical examination is **mandatory** when the **history is suggestive of secondary headache**, and then may elicit warning signs (Table 3).

Table 3. Warning features on examination, when associated with headache

Warning feature	What to beware of
Otherwise unexplained pyrexia	Meningitis
Neck stiffness	Meningitis or subarachnoid haemorrhage
Focal neurological signs	Secondary headache
Disorders of consciousness or memory	
Change in personality	
Weight-loss or poor general condition	

Investigation of headache patients

- Routine blood tests as a screen for general health may be worthwhile in primary care.
- **Special investigations**, including neuroimaging, are **not indicated unless** the history or examination suggests headache may be secondary to another condition.

Diagnostic caveats

The following tend to be **greatly overdiagnosed**:

- **cervicogenic headache** (headache caused by a disorder of the cervical spine and its component bony, disc and/or soft tissue elements, usually but not invariably accompanied by neck pain);
- headache attributed to **arterial hypertension** (chronic arterial hypertension below 180/110 mm Hg does not appear to *cause* headache);
- headache attributed to **refractive error** (rare in adults, although some evidence exists for it in children);
- headache attributed to "**sinusitis**" (a misdiagnosis commonly applied to migraine);
- **trigeminal neuralgia** (recurrent unilateral brief electric shock-like pains, abrupt in onset and termination, limited to the distribution of one or more divisions of the trigeminal nerve and triggered by innocuous stimuli);
- **occipital neuralgia** (paroxysmal shooting or stabbing pain in the posterior part of the scalp, in the distributions of greater, lesser and/or third occipital nerves).
