# Supplementary material for: Aids to management of headache disorders in primary care (2nd edition): on behalf of the European Headache Federation and Lifting The Burden: the Global Campaign against Headache
Source: J Headache Pain. 2019 May 21;20(1):57. doi: 10.1186/s10194-018-0899-2 (PMC6734476; doi:10.1186/s10194-018-0899-2)
Supplement: Supplementary file 4 — Guides to management: General aspects of headache management. (PDF 187 kb) [file 10194_2018_899_MOESM4_ESM.pdf]

4. General aspects of headache management

The purpose of these principles of management is to provide guidance, while demonstrating that headache **management in most cases is not difficult**.

The following are important for all headache disorders managed in primary care.

Educating and reassuring patients

Many people with recurrent headache wrongly fear underlying disease, so education and **appropriate reassurance** should never be omitted.

Good treatment of patients with any headache disorder therefore begins with **explanations** of their disorder and the purpose and means of management.

- Explanation is a crucial element of **preventative management** in patients with migraine or frequent episodic tension-type headache, who are at particular risk of escalating medication consumption.
- While patients want to know the **cause** of their headache, this may not be possible. Both genetic and environmental factors contribute to processes that are not well understood.
- Patients may need to be persuaded that **tests are not helpful**.
- Patients with primary headache disorders may be advised that these tend to **remit with advancing age**.

Advice on further information that may be requested by patients is provided in Supplementary materials #5.

A series of patient information leaflets provide basic explanations of migraine (Supplementary materials #21), tension-type headache (Supplementary materials #22), cluster headache (Supplementary materials #23), medication-overuse headache (Supplementary materials #24), trigeminal neuralgia (Supplementary materials #26) and persistent idiopathic facial pain (Supplementary materials #27), and their management.

Acknowledging and assessing impact

Assessment of impact **at start of treatment** establishes need and priority for treatment and measures the baseline for later evaluation of treatment. In addition to symptom-burden, impact of recurrent headache particularly includes disability.

The **HALT-90 Index** (Supplementary materials #18), developed by *Lifting The Burden*, is an easy-to-use instrument for assessing burden in terms of **lost productive time**.

In addition, recurrent disabling headache:

- may lead to **lifestyle compromise**, either in response to attacks or in a bid to avoid them (in this way, episodic headache can have continuous impact);
- has impact not only on the person with it but also on **other people** (family, work colleagues and employer).

Realistic aims of management

Primary headache disorders cannot be cured, but in most cases can be **effectively managed**. This means controlled by reductions in attack frequency and severity to minimise impact.

Causes and triggers

Many patients seek help in identifying triggers, but the importance of these should not be over-emphasised.

- Correctly identified triggers offer the possibility of **avoidance** (perhaps by life-style change) as a sometimes major contribution to management.
- When triggers are relevant to individual patients, they are usually **self-evident**.
- Triggers may be less readily identified when they are **cumulative** in their effect, jointly lowering the threshold above which attacks are initiated.
- Even when they are correctly identified, triggers are **not always avoidable**.

Follow-up

Every patient to whom treatment is offered, or whose treatment is changed, requires follow-up in order to ensure that optimum treatment has been established.

- The use of **outcome measures** is recommended to evaluate treatment and guide follow-up. The following are available:
  - the **HURT questionnaire** (Supplementary materials #20), developed by *Lifting The Burden* expressly to guide management in primary care;
  - the **HALT-30 Index** (Supplementary materials #19), to record lost productive time in the preceding month;

    - a headache calendar (see below).
- **Persistent management failure** is an indication for specialist referral.

Diaries and calendars

The principal distinction between these is in the amount of information collected. An example of each is available as Supplementary materials #16 and Supplementary materials #17.

Diaries capture more descriptive features of symptoms (headache intensity and character, associated symptoms), perhaps using free text.

- Diaries, used particularly as an aid to **diagnosis,** are useful for:
    - recording symptoms and temporal patterns that contribute to correct diagnosis;
    - recording acute medication use or overuse prior to diagnosis;
    - reporting lost productive time as part of pre-treatment assessment.

Calendars essentially note the temporal occurrence of headache episodes and related events such as menstruation and medication intake.

- Calendars, used in **follow-up**, are recommended in primary care for:
    - revealing associations with the menstrual cycle and possibly other triggers;
    - monitoring acute medication use or overuse during follow-up;
    - encouraging adherence to prophylactic medication;
    - recording treatment effect on headache frequency, and charting outcomes.
