# Supplementary material for: Aids to management of headache disorders in primary care (2nd edition): on behalf of the European Headache Federation and Lifting The Burden: the Global Campaign against Headache
Source: J Headache Pain. 2019 May 21;20(1):57. doi: 10.1186/s10194-018-0899-2 (PMC6734476; doi:10.1186/s10194-018-0899-2)
Supplement: Supplementary file 5 — Guides to management: Advice to patients. (PDF 190 kb) [file 10194_2018_899_MOESM5_ESM.pdf]

5. Advice to patients

Patients with headache disorders commonly request information. Many find or have found misleading information on the internet.

In addition to the advice below, **a series of patient information leaflets** are available in the Supplementary materials.

- Four describe the important headache disorders (**migraine** [Supplementary materials #21], **tension-type headache** [Supplementary materials #22], **cluster headache** [Supplementary materials #23] and **medication-overuse headache** [Supplementary materials #24]), and their management.
- A fifth offers information on **female hormones and headache** (Supplementary materials #25).
- Two further leaflets briefly describe **trigeminal neuralgia** (Supplementary materials #26) and **persistent idiopathic facial pain** (Supplementary materials #27).

Advice on non-drug treatments

Patients enquiring about the following may be given this summary advice.

- **Diets**. While healthy eating is always advisable, there is no reliable evidence that gluten-free, lactose-free, ketogenic or other specific diets prevent or improve headache disorders.
- **Biofeedback and relaxation therapies** can be helpful, and are potentially useful options when drug treatments must be avoided.
- **Cognitive behavioural therapy** may help patients develop coping strategies and better manage their symptoms. There is no good evidence to confirm benefit.
- **Physiotherapy** has proven benefits in some patients with tension-type headache. It requires skilled and individualised therapy, which is not widely available in many countries.
- **Aerobic exercise**. Limited data support the benefits of aerobic exercise on migraine and tension-type headache. Exercise has other important health benefits: improving physical strength, fitness and sleep, relieving depression and reducing blood pressure, cholesterol and weight.

- **Acupuncture** has differing forms, and is highly dependent on the skill of the therapist. There is **limited evidence** that acupuncture can be effective in reducing intensity and frequency of migraine attacks, but large clinical trials have failed to distinguish between acupuncture and sham procedures.
- **Devices**. Many are on the market, some very costly and promoted with insupportable claims of efficacy. "Testimonials" can be attributed to placebo effect and should be disregarded. The only clear recommendation possible is that successful trial usage should precede any expensive purchase.
  - A range of transcutaneous electrical nerve stimulators (TENS) and noninvasive neuromodulating devices for peripheral vagal nerve stimulation, supraorbital nerve stimulation and single-pulse transcranial magnetic stimulation are available, with evidence of efficacy in some people.
- **Herbals** are not recommended. Clinical trials data are limited and provide no evidence of safety in prolonged use. Herbals may interfere with other medications.
  - **Feverfew** preparations on sale everywhere are highly variable in content and their toxicity is not well understood.
  - **Butterbur** has some efficacy in migraine, but preparations on sale are variable in content and not all are free of liver toxins.
- **Nutraceuticals** are mostly not recommended. The following have some evidence for efficacy in migraine, and may be tried *where preparations of pharmaceutical quality are available*:
  - coenzyme Q10 (CoQ10 (100 mg three times daily);
  - magnesium (as citrate, starting at 100 mg three times daily to avoid diarrhoea, and increasing to 200 mg three times daily);
  - riboflavin (200 mg twice daily).
- **Homoeopathy** is of unproven value. There is no arguable case for over-the-counter sales of homoeopathic remedies.
- **Reflexology** has no scientific basis.
- **Cold packs or menthol gel** applied to the head and/or neck are found by some people to relieve pain or discomfort while being harmless and inexpensive.
- **Dental treatment**, including splints and bite-raising appliances, is of unproven value in treating headache and should be discouraged for this purpose.
- **Spectacles** should be professionally prescribed and worn when needed, but refractive errors are rarely a cause of troublesome headache.

  For the same reason, **accommodation training**, sometimes offered by optometrists, is not an accepted treatment for headache or likely to be beneficial.
- **Surgical procedures.** No surgical procedures produce benefit in migraine or tension-type headache. **Hysterectomy** has no place in migraine management.

Advice on hormonal contraception and HRT

With one important exception, migraine is not a contraindication to hormonal contraception or hormone replacement therapy (HRT).

- **Migraine with aura** and the **ethinylestradiol** component of combined hormonal contraceptives (CHCs) are **independent risk factors for stroke** in young women.
    - Every woman seeking hormonal contraception in primary care should be screened for migraine with aura and, if positive, offered **progestogen-only contraception** or non-hormonal alternatives.
- Otherwise, **headache is often a side-effect of CHCs** (pills, patches or vaginal rings), and many women report onset or aggravation of migraine after starting them.
    - Such symptoms usually resolve with continued use; if not, alternatives to CHCs should be offered.
- Other women, particularly those with menstrually-related migraine (without aura), report improvement, especially when CHCs are taken continuously without a week's break.

The following **advice on hormonal contraception** may be given to patients with migraine:

- CHCs **increase risk of stroke** in young women with **migraine with aura**, who should therefore use alternatives;
- **a change** from migraine without aura to migraine with aura after starting CHCs is a clear signal to **stop immediately**;
- **progestogen-only contraception** is acceptable with any type or subtype of migraine.

The following **advice on hormone replacement therapy (HRT)** may be given to patients with migraine:

- **HRT is not contraindicated** in migraine with or without aura;
- decisions about commencing or continuing HRT should be made according to generally applicable criteria.
