# Supplementary material for: Aids to management of headache disorders in primary care (2nd edition): on behalf of the European Headache Federation and Lifting The Burden: the Global Campaign against Headache
Source: J Headache Pain. 2019 May 21;20(1):57. doi: 10.1186/s10194-018-0899-2 (PMC6734476; doi:10.1186/s10194-018-0899-2)
Supplement: Supplementary file 6 — Guides to management: Management of migraine. (PDF 376 kb) [file 10194_2018_899_MOESM6_ESM.pdf]

# 6. Management of migraine

Migraine is typically a **moderate-to-severe headache** accompanied by **nausea, vomiting and sensitivity to light and/or noise**. It is commonly disabling. It is usually episodic, but there is an uncommon chronic form.

## Principles of management

- Good treatment of migraine begins with **education of patients**, explaining their disorder and the purpose and means of management.
- **Impact** of migraine should be assessed prior to planning treatment:
  - the **HALT-90 Index** (Supplementary materials #18) assesses burden in terms of lost productive time.
- **Triggers and predisposing factors** should not be overemphasised but should nonetheless be considered early in management (with life-style modification when called for).
- Almost all patients with migraine will require **drug therapy for acute attacks**, but not necessarily prescription drugs (see Supplementary materials #7).
- Any patient who is not well controlled with acute therapy alone and whose quality of life is impaired by migraine, whether adult or child, should be offered **prophylaxis** in addition (see Supplementary materials #8).
- Every patient to whom treatment is offered, or whose treatment is changed, requires **follow-up** to ensure that optimum treatment has been established.

## Education of patients

A patient information leaflet on migraine and its management, developed by *Lifting The Burden*, is available as Supplementary materials #21.

**Key points** of information are:

- migraine is a **common** disorder which, while it may be disabling, is **benign**;
- it is often **familial**, and probably genetically inherited;
- it cannot be cured but can be **successfully treated**;

- **trigger or predisposing factors** are common in migraine, and should be identified and avoided or modified when possible, but not all can be;
- a headache **calendar** helps good management by recording over time:
  - the symptoms and pattern of attacks (*eg*, menstrual relationship);
  - medication use (thus identifying overuse);
- **regular activity** (*eg*, sport or exercise 2-3 times per week) may reduce intensity and frequency of migraine attacks.

### Hormonal contraception and HRT

Many women report onset or aggravation of migraine after starting combined hormonal contraceptives (CHCs). Others, particularly those with menstrually-related migraine, report improvement, especially when CHCs are taken continuously without a week's break.

The following **advice on hormonal contraception** may be given:

- migraine with aura and the ethinylestradiol component of CHCs are **independent risk factors for stroke** in women, especially in those under 50 years;
- **alternatives to CHCs** are therefore very strongly recommended for women with migraine with aura;
- **a change** from migraine without aura to migraine with aura after starting CHCs is a clear signal to **stop immediately**;
- **progestogen-only contraception** is acceptable with any type or subtype of migraine.

The following **advice on hormone replacement therapy** (HRT) may be given:

- HRT is **not contraindicated** in migraine with or without aura;
- decisions about commencing or continuing HRT should be made according to generally applicable criteria.

A patient information leaflet on female hormones and headache, developed by *Lifting The Burden*, is available as Supplementary materials #25.

## Follow-up

- Use of a **calendar** is recommended to encourage adherence with prophylactic medication and record treatment effect. An example of a simple calendar is available as Supplementary materials #17.
- The use of **outcome measures** is recommended to guide follow-up. The following are included here among the management aids:
  - the **HURT questionnaire** (Supplementary materials #20) was developed expressly for primary care;
  - the **HALT-30 Index** (Supplementary materials #19) records lost productive time during the preceding month.

**Persistent management failure** is an indication for specialist referral.
