# Supplementary material for: Aids to management of headache disorders in primary care (2nd edition): on behalf of the European Headache Federation and Lifting The Burden: the Global Campaign against Headache
Source: J Headache Pain. 2019 May 21;20(1):57. doi: 10.1186/s10194-018-0899-2 (PMC6734476; doi:10.1186/s10194-018-0899-2)
Supplement: Supplementary file 7 — Guides to management: Management of migraine; Acute or symptomatic management of episodic migraine. (PDF 376 kb) [file 10194_2018_899_MOESM7_ESM.pdf]

in collaboration with

Lifting The Burden

European principles of management of headache disorders in primary care (2nd edition)

7. Acute or symptomatic management of episodic migraine

General principles

- All **adults** with episodic migraine should have access to acute medication.
- **Children** with short-lasting attacks may respond well to bed-rest without medical treatment.
- In adults and children, *regular* use of acute medication at high frequency (on >2 days/week) risks the development of **medication-overuse headache**.
- Many patients seek help in identifying **triggers** (see below). The importance of trigger factors in migraine is nonetheless often overemphasised.

Trigger and predisposing factors

- Correctly identified triggers offer the possibility of **avoidance** (perhaps by life-style change) as a sometimes major contribution to management.
- When triggers are relevant to individual patients, they are usually **self-evident**.
- **Cyclical hormonal fluctuations** may be an obvious factor in menstruating women.
- **Irregular lifestyle**, poor sleep pattern and "stress" are important predisposing factors in anybody with migraine. Missing meals is a potent trigger factor.
- Triggers may be less readily identified when they are **cumulative** in their effect, jointly lowering the threshold above which attacks are initiated.
- Even when they are correctly identified, triggers are **not always avoidable**.
- Contrary to popular belief, there is **no "migraine diet"**. The only dietary triggers with good evidential support are certain alcoholic drinks (especially red wine).

Drug intervention

All patients should climb a **treatment ladder** (stepped management), usually treating three attacks at each step before proceeding to the next. This strategy, when followed correctly, reliably achieves the most effective and cost-effective individualised care.

Step one: symptomatic therapy

- non-opioid analgesic
- plus, when needed, an antiemetic.

Recommended drugs and doses are shown in Table 1.

Table 1. Recommended drugs and doses for acute migraine therapy, step one

<table>
<tr><th>Analgesics</th><th>Antiemetics</th></tr>
<tr><td colspan="2">Adults</td></tr>
<tr><td>Non-steroidal anti-inflammatory drugs:
• acetylsalicylic acid 900-1000 mg or
• ibuprofen 400-800 mg or
• diclofenac 50-100 mg</td><td rowspan="3">• domperidone 10 mg (supportive evidence of efficacy is for 20 mg, but the European Medicines Agency recommends restriction to 10 mg orally [up to three times daily] or 30 mg by suppository [up to twice daily]), or
• metoclopramide 10 mg (the European Medicines Agency restricts dosing to 10 mg [up to three times daily])</td></tr>
<tr><td>or (where these are contraindicated):
• paracetamol 1000 mg*</td></tr>
<tr><td>or (possibly benefiting from the different mechanisms of action):
• combinations of paracetamol with acetylsalicylic acid or ibuprofen</td></tr>
<tr><td colspan="2">Children (when needed)</td></tr>
<tr><td>Ibuprofen 200-400 mg according to age and weight</td><td>• domperidone (dosage according to age and weight)</td></tr>
</table>

*Paracetamol on its own has lower efficacy and is **not** first-line treatment.

Drugs to avoid

- **Opioids** (including codeine and dihydrocodeine) are ineffective for migraine, associated with multiple adverse effects, potentially addictive and commonly implicated in medication-overuse headache;
- **Barbiturates** have no place in the treatment of migraine.

Principles of step one

- Use **soluble analgesics** (or mouth-dispersible formulations with water) when available.
- Take **early** in the attack.
- Use **adequate dosage** (see table 1: in most cases, adequate doses require more than a single tablet).
- A **prokinetic antiemetic counters gastric stasis**, an early feature of migraine, which impairs bioavailability of oral medication.
- Rectal formulations (where available) may be preferable in the presence of vomiting.
- Proceed to **step two after three attacks without success** (local guidelines may recommend trying more than one analgesic in step one before proceeding to step two).

Step two: specific therapy

- Where available, and unless contraindicated, specific therapy (Table 2) should be **offered to all patients failing step one**.
- Availability of drugs varies from country to country.

Table 2. Specific anti-migraine drugs, formulations and doses for step two (listed alphabetically)

Almotriptan	• tablets 12.5 mg
Eletriptan	• tablets 20 mg and 40 mg<br>• tablets 80 mg (not widely available)<br>(for some people, 80 mg is effective when 40 mg is not)
Frovatriptan	• tablets 2.5 mg
Naratriptan	• tablets 2.5 mg
Rizatriptan	• tablets and mouth-dispersible wafers 10 mg<br>• tablets 5 mg (to be used when propranolol is being taken concomitantly)
Sumatriptan	• tablets and rapidly dissolving tablets 50 mg and 100 mg<br>• nasal spray 10 mg (licensed for adolescents) and 20 mg<br>• subcutaneous injection 6 mg
Zolmitriptan	• tablets and mouth-dispersible tablets 2.5 mg and 5 mg<br>• nasal spray 5 mg

Drugs to avoid

- **Ergotamine** is a poor substitute for triptans: it has very low and unpredictable bioavailability, which impairs its efficacy, and poor tolerability. It is no longer recommended for routine use.

Principles of step two

- Triptans are more effective when taken while **headache is still mild** (but not during aura)
  (this instruction should be given only to patients who can reliably distinguish migraine from tension-type headache).
- The initial dose of all oral triptans (except eletriptan in some cases) is one tablet.
- A **second dose** for non-response is not recommended by most triptan manufacturers but, taken not less than 2 hours after the first, may nonetheless be effective in some cases.
- Triptans should **not be used regularly on ≥10 days/month** to avoid the risk of medication-overuse headache.
- Triptans differ slightly, but there are large and unpredictable individual **variations in responses** to them:
  - one may work where another has not;
  - patients are best served if they can try several, in different formulations, and choose between them.
- When **nausea** is present, domperidone 10 mg may be added.
- When **vomiting** is present, zolmitriptan nasal spray (absorbed through the nasal mucosa) or sumatriptan subcutaneous injection may be preferred.
- Efficacy of sumatriptan may be increased by combination with naproxen 500-1000 mg (there are no data on combinations of other triptans and NSAIDs).
- When all other triptans are ineffective, sumatriptan by subcutaneous injection 6 mg should be considered.
- Triptans are associated with return of symptoms within 48 hours (**relapse**) in up to 40% of patients who have initially responded (see below).

Treatment of relapse

- A **repeat dose** of a triptan is usually effective.
- A further relapse may occur:
  - in a minority of patients, this happens **repeatedly**, a major management problem with high risk of developing **medication-overuse headache**;
  - a different triptan should be tried in future attacks;
  - concomitant use of a triptan and naproxen may reduce susceptibility to relapse.

Contraindications and special precautions in step two

- Triptans should not be taken **during aura** of migraine with aura, but at the onset of headache.
- All triptans should be **avoided** by people with:
  - uncontrolled hypertension (one reason for measuring blood pressure);
  - coronary heart disease, cerebrovascular disease or peripheral vascular disease;
  - multiple risk factors for coronary or cerebrovascular disease;
- In the **elderly**, all of these are more common, and triptans should therefore be used with **greater caution**.
- In **pregnancy**: limited safety data are available only for sumatriptan, which should be used with caution and **only under specialist supervision**.
- In addition, there are **specific precautions** attached to some triptans (see pharmacopoeia).

Step two for children and adolescents

- **Failure of step one in children is an indication for specialist referral**.
  - No specific anti-migraine drug has been shown to have efficacy in children (under 12 years old).
- **For adolescents** (12-17 years), the following have efficacy and are approved:
  - sumatriptan nasal spray 10 mg;
  - zolmitriptan nasal spray 2.5 mg and/or 5 mg (in some countries).

Follow-up

Every patient to whom treatment is offered, or whose treatment is changed, requires follow-up to ensure that optimum treatment has been established.

- Use of a **calendar** is recommended to monitor acute medication use or overuse. An example of a simple calendar is available as Supplementary materials #17.
- The use of **outcome measures** is recommended to guide follow-up. The following are included here among the management aids:
  - the **HURT questionnaire** (Supplementary materials #20) was developed expressly for primary care;
  - the **HALT-30 Index** (Supplementary materials #19) records lost productive time during the preceding month.
- **Failure of acute therapy** may be an indication for prophylaxis (see Supplementary materials #8).
