# Supplementary material for: Aids to management of headache disorders in primary care (2nd edition): on behalf of the European Headache Federation and Lifting The Burden: the Global Campaign against Headache
Source: J Headache Pain. 2019 May 21;20(1):57. doi: 10.1186/s10194-018-0899-2 (PMC6734476; doi:10.1186/s10194-018-0899-2)
Supplement: Supplementary file 8 — Guides to management: Management of migraine; Prophylactic management of episodic migraine. (PDF 192 kb) [file 10194_2018_899_MOESM8_ESM.pdf]

in collaboration with

Lifting The Burden

European principles of management of headache disorders in primary care (2nd edition)

8. Prophylactic management of episodic migraine

General principle

Any patient with migraine who is **not well controlled with acute therapy alone**, whether adult or child, should be offered prophylaxis **in addition to** acute medication.

Indications for prophylaxis

Prophylactic therapy should be **added** when migraine **impairs quality of life**, *and*

- attacks cause disability on **two or more days per month**, *and*
- **acute therapy has been optimised** but does not prevent this, or is poorly tolerated, *or*
- there is a risk of **over-frequent use of acute therapy**, even when it is effective; *and*
- the **patient is willing** to take daily medication.

Frequent absences from school because of migraine are an additional indication for prophylaxis in **children** (who should be referred for specialist assessment).

Principles of prophylaxis

- A **calendar** should be kept by every patient on prophylaxis to assess efficacy and promote adherence. An example of a simple calendar is available as Supplementary materials #14.
- **Poor adherence** is a major factor impairing efficacy of migraine prophylactics; once-daily dosing is associated with better adherence.
- The **dose** of any drug **should start low** in the suggested range and be increased in the absence of troublesome side-effects.

- Drugs that appear ineffective should **not be discontinued too soon**; 2-3 months may be the minimum to achieve and observe efficacy.
- Failure of one drug **does not predict failure of others** in a different class.
- **Tapered withdrawal** may be considered after 6 months of good control, and should be considered no later than after 1 year.
- **Children** requiring prophylactic medication should be referred for specialist assessment.

Table 1. Migraine prophylactic drugs with evidence of efficacy in adults (beta blockers are listed alphabetically; otherwise, drugs are listed in a suggested order of use)

Beta-adrenergic blockers without partial agonism:<br>• atenolol 25-100 mg twice daily<br>• bisoprolol 5-10 mg once daily<br>• metoprolol 50-100 mg twice daily *or* modified-release 200 mg once daily<br>• propranolol LA 80-160 mg once to twice daily	• observe general contraindications, including comorbid depression<br>• propranolol has best evidence of efficacy, but not evidence of best efficacy<br>• cardioselective and non-lipophyllic drugs (bisoprolol, atenolol, metoprolol) are likely to be better tolerated
Amitriptyline 10-100 mg at night	• may be preferred when migraine coexists with tension-type headache, depression or sleep disturbance
Topiramate 50 mg twice daily	• titrate over 4 weeks from 25 mg once daily<br>• contraindicated in pregnancy
Candesartan 16 mg once daily	• start at 8 mg once daily and titrate weekly<br>• contraindicated in pregnancy
Sodium valproate 600-1500 mg daily	• titrate upwards<br>• **avoid altogether in women of child-bearing potential** (even on contraception); absolutely contraindicated in pregnancy
Flunarizine 5-10 mg once daily	• observe general contraindications, including comorbid depression
CGRP monoclonal antibodies (to the peptide or its receptor):<br>• erenumab 70 or 140 mg s/c once monthly<br>• fremanezumab 225 mg s/c once monthly or 675 mg s/c once quarterly<br>• galcanezumab 240 mg s/c, then 120 mg s/c once monthly	• newly licensed, not yet universally available or reimbursed, usually restricted to specialist care and reserved for those failing (or not tolerating) other prophylactics<br>• all self-administered by auto-injector<br>• high relative cost

Effective drugs for prophylaxis

A range of drugs have proven efficacy (Table 1), all with contraindications and side-effects (refer to pharmacopoeia).

- Availability and regulatory approval vary from country to country, and many are not specifically licensed for migraine prophylaxis. Use of drugs off-licence rests on individual clinical responsibility.
- Across the range, expected benefit is no greater than 50% fewer attacks in 50% of users after 3 months of treatment (with individual benefit varying between zero and [rarely] 100%).
- Once daily dosing is associated with better adherence, an important determinant of efficacy.

Other treatments patients may ask about

- **Onabotulinum toxin A (Botox)**. This is **not effective** in episodic migraine and is not recommended for this condition.
- **Surgical procedures**. There is **no evidence** to support any surgical procedure as a treatment for episodic migraine.
  - In particular, migraine is not improved by closure of patent foramen ovale (PFO). This procedure should not be undertaken for migraine prophylaxis: it carries a small but relevant risk of serious adverse events including stroke, pericardial tamponade, atrial fibrillation and death.
- **Acupuncture** has differing forms, and is highly dependent on the skill of the therapist. There is **limited evidence** that acupuncture can be effective in reducing intensity and frequency of migraine attacks, but large clinical trials have failed to distinguish between acupuncture and sham procedures. Benefits experienced by some patients may be attributable to placebo effect.
- **Devices**. Many are on the market, some very costly and promoted with insupportable claims of efficacy. "Testimonials" can be attributed to placebo effect and should be disregarded. The only clear recommendation possible is that successful trial usage should precede any expensive purchase.
  - A range of transcutaneous electrical nerve stimulators (TENS) and noninvasive neuromodulating devices for peripheral vagal nerve, supraorbital nerve and single-pulse transcranial magnetic stimulation are available, with evidence of efficacy in some people.
- **Herbals** are **not recommended**. Evidence of both efficacy and safety in prolonged use is poor. They may interfere with other medications.
  - **Feverfew** preparations are highly variable in content, and not all of pharmaceutical quality. Their toxicity is not well understood.
  - **Butterbur** has some efficacy and is approved for use in some countries, but preparations on sale are variable in content and not all of pharmaceutical quality (not guaranteed to be free of liver toxins).
- **Nutraceuticals** are mostly not recommended. The following have some evidence for efficacy, and may be tried where preparations of pharmaceutical quality are available:
  - **coenzyme Q10** (CoQ10) (100 mg three times daily);

  - **magnesium** (as citrate, starting at 100 mg three times daily to avoid diarrhoea, and increasing to 200 mg three times daily);
  - **riboflavin** (200 mg twice daily).
- **Homoeopathy** is of unproven value. There is no arguable case for over-the-counter sales of homoeopathic remedies.

Prophylaxis in pregnancy

- This is **better avoided**, and rarely required since migraine often remits during pregnancy.
- Sodium valproate is **absolutely contraindicated**; topiramate and candesartan are contraindicated.
- **Propranolol and amitriptyline** have best evidence of safety, but specialist guidance is recommended.
- Riboflavin (vitamin B2), 200 mg twice daily, may be tried, but may not show efficacy for 3 months.

Follow-up

Every patient to whom prophylactic treatment is offered, or whose treatment is changed, requires follow-up to ensure that optimum treatment has been established.

- Use of a **calendar** is recommended to encourage adherence with prophylactic medication and record treatment effect. An example of a simple calendar is available as Supplementary materials #14.
- The use of **outcome measures** is recommended to guide follow-up. The following are included here among the management aids:
  - the **HURT questionnaire** (Supplementary materials #17) was developed expressly for primary care;
  - the **HALT-30 Index** (Supplementary materials #16) records lost productive time during the preceding month.

When prophylaxis fails

- Failure may be due to subtherapeutic dosage (itself perhaps due to non-adherence) or insufficient duration of treatment.
- The following actions are recommended:
  - review the **diagnosis**;
  - review adherence;
  - review **other medication**, especially for **overuse**.
- When prophylaxis still fails to have clear benefit, **discontinue** it.
- When all options fail, **specialist referral** is indicated.
