# Supplementary material for: Aids to management of headache disorders in primary care (2nd edition): on behalf of the European Headache Federation and Lifting The Burden: the Global Campaign against Headache
Source: J Headache Pain. 2019 May 21;20(1):57. doi: 10.1186/s10194-018-0899-2 (PMC6734476; doi:10.1186/s10194-018-0899-2)
Supplement: Supplementary file 9 — Guides to management: Management of migraine; Management of chronic migraine. (PDF 136 kb) [file 10194_2018_899_MOESM9_ESM.pdf]

9. Management of chronic migraine

Chronic migraine develops in a small minority of people with episodic migraine. It is one of the syndromes characterised by **headache on ≥15 days/month**, but is **not simply migraine that is more frequent**: it is often complicated by medication overuse, depression, anxiety and low back and/or neck pain.

Chronic migraine should be:

- **suspected** in any patient:
  - with a history of migraine
  - who reports (or records in a diary) **headache on ≥15 days/month**;
- **diagnosed**, in the absence of medication overuse, in patients with:
  - **headache on ≥15 days/month** over the last 3 months, which
  - on **≥8 days/month**:
    - fulfilled the diagnostic criteria for migraine, *or*
    - responded to migraine-specific drug treatment.

The **presence of medication overuse** in such patients complicates the diagnosis:

- medication-overuse headache (MOH) is another syndrome characterised by headache on ≥15 days/month;
- chronic migraine and MOH are **not mutually exclusive** but, even when the conditions above are met, only MOH and not chronic migraine may be present when medication is being overused;
- medication overuse, whether or not occurring with chronic migraine, must **always be recognised and managed as a separate medical problem**.

Medication-overuse, and MOH, can often be successfully managed in primary care (see Supplementary materials #9), but patients with **chronic migraine** should be **referred for specialist care**.

Principles of management

Chronic migraine is **difficult to treat**. Management in specialist care includes:

- **education** of patients about chronicity and its causes and risk factors;
- recognition and management of **medication overuse**, when present;
- management of any **comorbidities**;
- use of **preventative drugs** (Table 1);
- **follow up**, with both medical and psychological care.

Preventative drugs

Those used in specialist care, with evidence of efficacy, are shown in Table 1.

Table 1. Drugs used by specialists in chronic migraine prophylaxis

<table>
<tr><td colspan="2">Topiramate, 50 mg or more twice daily</td></tr>
<tr><td>Onabotulinum toxin A, 155-195 units by multisite injection</td><td>• not licensed for chronic migraine in some countries, or
• not reimbursed, and/or
• regulators require prior failure of two or more of the drugs used in prophylaxis of episodic migraine</td></tr>
<tr><td>CGRP monoclonal antibodies (to the peptide or its receptor):
• erenumab 70 or 140 mg s/c once monthly
• fremanezumab 225 mg s/c once monthly or 675 mg s/c once quarterly
• galcanezumab 240 mg s/c, then 120 mg s/c once monthly</td><td>• newly licensed, not yet universally available or reimbursed, usually restricted to specialist care and reserved for those failing (or not tolerating) other prophylactics
• all self-administered by auto-injector
• high relative cost</td></tr>
</table>
