# Supplementary material for: Aids to management of headache disorders in primary care (2nd edition): on behalf of the European Headache Federation and Lifting The Burden: the Global Campaign against Headache
Source: J Headache Pain. 2019 May 21;20(1):57. doi: 10.1186/s10194-018-0899-2 (PMC6734476; doi:10.1186/s10194-018-0899-2)
Supplement: Supplementary file 10 — Guides to management: Management of tension-type headache. (PDF 217 kb) [file 10194_2018_899_MOESM10_ESM.pdf]

in collaboration with

Lifting The Burden

European principles of management of headache disorders in primary care (2nd edition)

10. Management of tension-type headache (TTH)

Tension-type headache (TTH) is typically a **mild-to-moderate headache** of highly variable frequency and duration, **without associated symptoms** or the specific features of migraine.

Two types of TTH are medically important:

- **frequent episodic TTH**, with headache attacks on 1-14 days/month on average;
- **chronic TTH**, one of the syndromes characterised by headache occurring on ≥15 days/month, either with highly-frequent attacks or, occasionally, continuous and unremitting.

General principles

- Good treatment of patients with troublesome TTH (of either type) begins with their **education**, explaining their disorder and the purpose and means of management.
- **Impact** of TTH should be assessed prior to planning treatment:
    - the **HALT-90 Index** (Supplementary materials #18) assesses burden in terms of lost productive time.
- **Infrequent** headaches (on ≤2 days/week) are managed with over-the counter (OTC) analgesics.
- When headache is **more frequent**:
    - advice on **lifestyle** may be helpful, possibly accompanied by psychological intervention such as cognitive behavioural therapy;
    - analgesics (even OTC) should be used with care because of the risk of **medication-overuse headache**;
    - **prophylaxis** may be indicated.

Education of patients

A patient information leaflet on TTH and its management, developed by *Lifting The Burden*, is available as Supplementary materials #22.

Key points of information are:

- TTH is a **very common** disorder but, while it may be disabling and troublesome when headaches are frequent, it is **benign**;
- **episodic TTH** can be successfully treated, usually with OTC analgesics;
- **over-frequent use** of medications, even OTC, will make headaches worse;
- **chronic TTH** cannot be regularly treated with analgesics and usually requires other long-term continuous medication and/or non-pharmacological interventions;
- a headache **calendar** (Supplementary materials #17) helps good management by recording over time the symptoms and pattern of attacks and medication use;
- **predisposing factors** sometimes include stress and/or poor head and neck posture;
- **regular activity** (*eg*, sport or exercise 2-3 times per week) may help frequent TTH.

Acute intervention

Symptomatic treatment with OTC analgesics (Table 1) is appropriate for episodic TTH occurring on ≤**2 days/week**.

Table 1. Analgesics for episodic tension-type headache

Ibuprofen 400-800 mg	• for adults, and<br>• drug of choice for children (200-400 mg according to age and weight)
Acetylsalicylic acid 600-1000 mg	• adults only
Either of these in combination with paracetamol 1000 mg	• formal evidence is lacking, but the different mechanisms of action may enhance effect
Any of these in combination with caffeine	• commonly included in analgesic combination-medications
Paracetamol 1000 mg	• on its own has lower efficacy<br>• therefore reserved for those in whom NSAIDS are contraindicated

Drugs to avoid

- **Opioids** (including codeine and dihydrocodeine) are ineffective for headache, associated with multiple adverse effects, potentially addictive and commonly implicated in medication-overuse headache.
- **Barbiturates** have no place in the treatment of TTH.
- **Metamizol** has limited evidence for efficacy and is associated with agranulocytosis.
- **Triptans** are specific for migraine, and ineffective in TTH.

Principles of acute intervention

- Episodic TTH occurring on ≤**2 days/week** can usually be successfully treated with **OTC analgesics alone**;
- As the **frequency of headaches increases**, so does the risk of medication overuse:
  - episodic TTH on **>2 days/week** is a clear indication for **prophylaxis** (see below) in place of, rather than in addition to, acute intervention;
  - acute treatments are **unlikely to be effective in chronic TTH** and put the patient at clear risk of medication-overuse headache.

Prophylaxis

Principles of prophylaxis

- A **calendar** should be kept to assess efficacy and promote adherence. An example of a simple calendar is available as Supplementary materials #17.
- Patients receiving medication more often used as an antidepressant should be **advised of this, and why**; otherwise, they may default when they find out.
- Prophylaxis that appears **ineffective** should not be discontinued too soon; 2-3 months may be the minimum to achieve and observe efficacy.
- **Tapered withdrawal** may be considered after 6 months of good control, but prolonged treatment is sometimes indicated.

Effective drugs

A narrow range of drugs have efficacy (Table 2), although none is specifically licensed for TTH prophylaxis. Use of drugs off-licence rests on individual clinical responsibility.

Table 2. Prophylactic drugs with some evidence of efficacy in frequent episodic or chronic tension-type headache

amitriptyline, 10-100 mg at night	• drug of choice for frequent episodic or chronic TTH;<br>• intolerance is reduced by starting at a low dose (10 mg) and incrementing by 10-25 mg each 1-2 weeks
nortriptyline (replacing amitriptyline at the same dose)	• fewer anticholinergic side-effects but less good evidence of efficacy
mirtazapine, 15-30 mg once daily	• second-line option
venlafaxine, 75-150 mg once daily	• third-line option

Drugs to avoid

- **Onabotulinum toxin A** is ineffective in TTH.

Non-pharmacological prophylaxis

- There is limited evidence that **acupuncture** is effective in reducing intensity and frequency of TTH episodes. While some patients experience benefit, this may be due to placebo effect. Acupuncture has differing forms, and is highly dependent on the skill of the therapist.
- There is well-documented evidence of efficacy of various forms of **biofeedback**. They are highly dependent on the skill of the therapist.

Follow-up

Every patient to whom treatment is offered, or whose treatment is changed, requires follow-up to ensure that optimum treatment has been established.

- Use of a **calendar** is recommended to monitor acute medication use or overuse, or to encourage adherence to prophylactic medication, and to record treatment effect. An example of a simple calendar is available as Supplementary materials #17.
- The use of **outcome measures** is recommended to guide follow-up. The following are included here among the management aids:
  - the **HURT questionnaire** (Supplementary materials #20) was developed expressly for primary care;
  - the **HALT-30 Index** (Supplementary materials #19) records lost productive time during the preceding month.

When prophylaxis fails

- Failure may be due to subtherapeutic dosage (itself perhaps due to non-adherence) or insufficient duration of treatment.
- The following actions are recommended:
  - review the **diagnosis**;
  - review **adherence**;
  - review **other medication**, especially for **overuse**;
- When prophylaxis still fails to have clear benefit, **discontinue** it.
- When all options fail, **specialist referral** is indicated.

Pain management

- Despite best efforts, **chronic TTH is often refractory** to medical treatment or may become so.
- Patients in this situation require referral into a pain management programme with emphasis on **psychological approaches**.
