# Supplementary material for: Aids to management of headache disorders in primary care (2nd edition): on behalf of the European Headache Federation and Lifting The Burden: the Global Campaign against Headache
Source: J Headache Pain. 2019 May 21;20(1):57. doi: 10.1186/s10194-018-0899-2 (PMC6734476; doi:10.1186/s10194-018-0899-2)
Supplement: Supplementary file 11 — Guides to management: Management of cluster headache. (PDF 207 kb) [file 10194_2018_899_MOESM11_ESM.pdf]

in collaboration with

Lifting The Burden

European principles of management of headache disorders in primary care (2nd edition)

11. Management of cluster headache

Cluster headache, a type of trigeminal autonomic cephalalgia, is characterised by frequently recurring, localised, short-lasting but **extremely severe headache**, which is accompanied by a set of highly characteristic **autonomic symptoms**.

- Cluster headache is **easily recognisable** (see Supplementary materials #2).
- It should **never be missed**.

It has **two subtypes**:

- **episodic cluster headache**, with attacks occurring in bouts (clusters) that last for a few or many weeks and then remit for ≥3 months;
- **chronic cluster headache**, less common, but persisting without remissions, or with remissions of <3 months.

General principles

- Patients with this disorder **suffer very badly** if ineffectively treated:
    - cluster headache management is, at least initially, **better left to specialists** who see this disorder frequently;
    - on first presentation it demands **accelerated referral** for investigation and treatment;
    - **recognition** in primary care is crucial to ensure prompt referral.
- The **objective** of management in both episodic and chronic subtypes is **total attack suppression**. This is not always achievable.
- Both acute medication and prophylaxis have a role in management, but **preventative drugs are the mainstay of treatment** in most cases.
- Once effective treatment has been established, future clusters, or maintenance therapy in the case of chronic cluster headache, may be managed in primary care.

Acute therapies

There are limited options (Table 1), but efficacy may be high.

- Availability varies between countries.
- Most are not specifically licensed for cluster headache. Use of drugs off-licence rests on individual clinical responsibility.

Table 1. Acute therapies used in cluster headache by specialists

**Triptans:**<br>• sumatriptan 6 mg s/c<br>• zolmitriptan 5 mg nasal spray<br>• sumatriptan 20 mg nasal spray	none can be recommended for use more than twice a day<br>• the most highly-effective acute treatment<br>• less-certain efficacy but an alternative for those unable or unwilling to use sumatriptan s/c<br>• less-certain efficacy: absorption depends largely on ingestion
Oxygen 100% at ≥12 l/min until response, or for ≥15 minutes	• requires a non-rebreathing mask and regulator;<br>• helps some people and may be used as frequently as needed

Drugs to avoid

- **Oral triptans** are slow in onset of action and are not useful substitutes.
- **Analgesics**, including opioids, have little or no place in treating cluster headache.

Preventative therapy

Specialists employ the following:

- **transition therapy** (Table 2), used at onset of treatment to achieve more rapid response during dose escalation of any of the preventative drugs;
- **maintenance prophylaxis** (Table 3), balancing efficacy of drugs against their significant toxicity (refer to pharmacopoeia).

Table 2. Transition therapies used in cluster headache by specialists

Prednisolone 60-80 mg once daily	• for 2-4 days, discontinued by dose reduction over 1-3 weeks
Greater occipital nerve blockade	• using various agents

Table 3. Drugs used by specialists in maintenance prophylaxis of cluster headache

Verapamil 240-960 mg daily	• ECG monitoring advised
Lithium carbonate 600-1600 mg daily	• serum levels must be regularly monitored
Topiramate 50-100 mg twice daily	• less evidence of efficacy, but no monitoring required

Principles of preventative therapy

- **Prophylaxis** of episodic cluster headache should begin **as early as possible** after the start of a new cluster bout.
- **Failure** of one drug does not predict failure of others.
- **Combinations** of drugs may be tried, but the potential for toxicity is obviously high.
- For episodic cluster headache, maintenance prophylaxis should be **discontinued** by tapering, usually 2 weeks after full remission.
- For chronic cluster headache, maintenance prophylaxis may need to be continued **long-term**.

Other treatment options

- **Neuromodulation**, non-invasive or invasive, is occasionally used by specialists.

Follow-up

Every patient with active cluster headache requires **frequent follow-up** both to ensure that optimum acute and preventative treatments are maintained and to **monitor for treatment toxicity**.

- Patients with episodic cluster headache in remission should be advised to **return promptly** at the onset of the next cluster episode.

Information for patients

A patient information leaflet on cluster headache and its management, developed by *Lifting The Burden*, is available as Supplementary materials #23.
