# Supplementary material for: Aids to management of headache disorders in primary care (2nd edition): on behalf of the European Headache Federation and Lifting The Burden: the Global Campaign against Headache
Source: J Headache Pain. 2019 May 21;20(1):57. doi: 10.1186/s10194-018-0899-2 (PMC6734476; doi:10.1186/s10194-018-0899-2)
Supplement: Supplementary file 12 — Guides to management: Management of medication-overuse headache. (PDF 190 kb) [file 10194_2018_899_MOESM12_ESM.pdf]

12. Management of medication-overuse headache (MOH)

Medication-overuse headache (MOH) is one of the syndromes characterised by **headache occurring on ≥15 days/month**. It is often daily, but variable in site, intensity and character. It greatly impairs quality of life.

MOH is an aggravation of a prior headache disorder (usually migraine, but sometimes tension-type headache) caused by chronic overuse of medication taken to treat it.

General principles

- **Prevention**, through **education**, is preferable to cure.
- Once MOH has developed, **early intervention** has better chance of success.
- The **necessary management** of established MOH is to **stop overuse** of the suspected medication(s).
- **Patient education**, that medication taken to relieve headache is in fact its cause, is the essential first step:
    - success in management depends crucially on **patients' understanding** that their medication taken to relieve their headache is in fact its cause.
- Management is usually possible in **primary care**.
- The **long-term prognosis is usually very good**. Most cases revert to episodic headache, although the outcome depends on:
    - the type of headache from which MOH developed;
    - the class of medication overused (opioids causing greatest difficulty);
    - the **duration of overuse**;
    - **comorbidities** (psychiatric, or other causes of chronic pain).

Education of patients

A patient information leaflet on medication-overuse headache and its management, developed by *Lifting The Burden*, is available as Supplementary materials #24.

Key points of information are:

- The "treatment" a patient is taking for headache is actually **the cause** of it.
- Effective treatment requires, in the first instance, **stopping use of the suspected medication(s)** (withdrawal):
  - there is **no other option**;
  - many patients recover from this alone.
- **Initial worsening** of symptoms for 1-2 weeks during and after withdrawal must be expected.
- The **outcome is usually very good**, with reversion in most cases, within 2 months, to the antecedent episodic headache disorder.

Objectives

There are **four separate objectives** in the complete management of MOH, and all are important:

- **stop** the overused medication;
- **recovery** from MOH (which should follow);
- **review** and reassess the underlying headache disorder (usually migraine or tension-type headache);
- **prevent relapse**, while allowing acceptable use of medications.

In addition, **comorbidities** may require management.

Principles of withdrawal

- **Worsening headache** for 1-2 weeks is almost inevitable:
  - accordingly, withdrawal should be **planned** to avoid unnecessary lifestyle disruption;
  - 1-2 weeks' **sick leave** may be needed;
  - **admission to hospital** during withdrawal is **rarely necessary** unless:
    - overused medication(s) include opioids;
    - for management of comorbidities.
- **Withdrawal** may be undertaken in **any of three ways**, the choice being made by the patient:
  - **abruptly**:
    - there is evidence that this is the most successful approach;

  - by **tapering** over a period of 2-4 weeks:
    - withdrawal symptoms are likely to be less intense but more prolonged;
  - by **replacing** the overused medication(s) with **naproxen 500 mg twice daily** for 3-4 weeks and no longer:
    - the purpose is to break the behavioural "have headache – take medication" link;
    - many patients become headache-free on this medication;
    - naproxen must be stopped after this period (never continued).
- Headache usually shows signs of **improvement** 1-2 weeks after stopping overused medication(s).
- **Recovery** continues slowly for up to 2 months.
- **Prophylaxis** against the antecedent headache (most often migraine) may be introduced on its return, or commenced in parallel with the withdrawal process.

Follow-up

Every patient stopping medication overuse requires follow-up in order to provide support and observe outcome.

- **First review** is advised **after 2-3 weeks** to ensure withdrawal has been successfully achieved.
- Use of a **calendar** during withdrawal is strongly recommended to record symptoms and medication use, and to record changing headache pattern. An example of a simple calendar is available as Supplementary materials #17.
- Most patients revert to their **antecedent headache** (usually migraine or tension-type headache) within 2 months; this will need review and appropriate management.
- The **relapse** rate is high within the first year: further follow-up is important to avoid it, and many patients require extended support.

Re-introducing withdrawn medication

- Previously overused medications should be reassessed:
  - **alternatives** should be used whenever possible;
  - if still needed, they may be **cautiously reintroduced** after 2 months.
- Frequency of use should be on **no more than 10 days/month**:
  - use on more than 6 days/month raises the risk of recidivism;
  - patients should avoid treating headaches on more than three days in a row.
