# Supplementary material for: Aids to management of headache disorders in primary care (2nd edition): on behalf of the European Headache Federation and Lifting The Burden: the Global Campaign against Headache
Source: J Headache Pain. 2019 May 21;20(1):57. doi: 10.1186/s10194-018-0899-2 (PMC6734476; doi:10.1186/s10194-018-0899-2)
Supplement: Supplementary file 13 — Guides to management: Management of trigeminal neuralgia and persistent idiopathic facial pain. (PDF 185 kb) [file 10194_2018_899_MOESM13_ESM.pdf]

in collaboration with

Lifting The Burden

European principles of management of headache disorders in primary care (2nd edition)

13. Management of trigeminal neuralgia and persistent idiopathic facial pain

Management of these uncommon but troublesome disorders is **better left to specialists**.

- **Recognition in primary care is crucial** to ensure prompt referral.

Trigeminal neuralgia (TN)

This disorder presents as recurrent, unilateral, **brief but severe, electric-shock-like pains** in the distribution of the trigeminal nerve, abrupt in onset and termination and often triggered by innocuous stimuli.

It is not common, affecting 1-2 in every 1,000 people. Women are twice as likely to be affected as men.

Principles of management

- TN is **extremely painful**, and untreated is physically, psychologically and socially debilitating:
    - patients may avoid the triggers of eating and drinking, **seriously impairing food and fluid intake**.
    - TN therefore demands **accelerated specialist referral** for investigation and treatment.
- Good treatment begins with **education of patients**, explaining their disorder and the purpose and means of management.
- The **objective** in management, by medical or surgical means, is abatement of attacks and pain freedom. This is not always achievable.
- **MRI is mandatory** since classical TN and secondary TN (due usually to cerebellopontine angle tumour, AV-malformation or multiple sclerosis) may be indistinguishable by symptom presentation.
- **First-line** treatment is prophylactic (antiepileptic) medication.

- Acute therapies (opioids or other analgesics) have **no place** in management since attacks are very short-lasting.
- Severe exacerbations with anorexia and dehydration, due to pain triggered by eating or drinking, may require **hospital admission** for intravenous hydration and medication.

Education of patients

A patient information leaflet on trigeminal neuralgia is available as Supplementary materials #26.

Key points of information are:

- TN produces very characteristic, **very severe, electric-shock-like pains**:
    - along a nerve on one side of the face, usually in the **cheek or jaw**;
    - repetitively, in short-lasting bouts (up to 2 minutes), which:
        - occur **daily for weeks or months** but sometimes remit spontaneously;
        - usually **start without warning**, but can be **provoked** by light touch, wind, cold air, eating, drinking, brushing the teeth or speaking.
- The **cause** of TN is often not known:
    - some people have a blood vessel in close contact with and compressing the affected nerve: an **MRI brain scan** is required to show this;
    - however, there are other unknown causes.
- **Specialist referral** is therefore necessary.
- There are a **number of treatments** for TN, which often work well:
    - these are **preventative** medications, to be taken daily;
    - **painkillers do not help**;
    - occasionally, surgery is required, but as a last resort;
    - TN does **not require dental treatment**.

Table 1. Drugs used by specialists in trigeminal neuralgia prophylaxis

**First line:**<br>• carbamazepine 200-2400 mg daily<br>• oxcarbazepine 600-2400 mg daily	these drugs:<br>• reduce efficacy of oral contraceptives;<br>• may induce hyponatraemia (especially oxcarbazepine): regular monitoring is advised;<br>• may induce osteoporosis in long-term treatment: prophylaxis against this is advised
**Second-line** (either as monotherapy or as add-on medication):<br>• gabapentin 600-3600 mg daily<br>• pregabalin 150-600 mg daily<br>• lamotrigine 200-1000 mg daily (very slow up-titration necessary)	

Preventative medications

A narrow range of antiepileptic drugs are effective, and used by specialists (Table 1). **Maximum dosages** may be necessary to achieve pain relief, and balancing efficacy against toxicity is difficult.

Principles of drug prophylaxis

- Dosages should be **up-titrated slowly** until pain relief is achieved or side effects become unacceptable.
- Patients established on medication may be taught to **titrate up and down**, according to symptom severity.
- **Combinations** may cause fewer side-effects because lower doses may be required of each drug.
- Treatment may be **slowly tapered** after complete freedom from pain, and discontinued in the absence of relapse.

Other treatment options in medically refractory patients

- **Neurosurgical treatments** are relevant when medical treatment with maximum tolerated doses achieve insufficient efficacy, but:
  - microvascular decompression (appropriate when neurovascular compression, not merely contact, has been demonstrated) carries a small risk of severe complications such as cranial nerve palsy or stroke;
  - gamma-knife and/or percutaneous procedures (balloon compression, glycerol injection, thermocoagulation or pulsed radiofrequency treatment) targeting the trigeminal ganglion are less invasive but probably less efficacious.

Follow-up

While every patient with TN requires specialist initial management, long-term follow-up once stable is appropriate in primary care.

- Patients should be educated on:
  - how to **taper medication** cautiously once pain freedom is achieved;
  - how to **reintroduce medication** by careful up-titration if/when pain returns.

Persistent idiopathic facial pain (PIFP)

Previously termed "atypical facial pain", this disorder presents as dull, aching or nagging, **poorly localized facial and/or oral pain**, which recurs daily for >2 hours over >3 months. Only rarely are there electric-shock-like pain attacks as in trigeminal neuralgia.

PIFP is rare, mostly affecting younger women, but it can start at any age.

Principles of management

- PIFP is painful, and can be physically, psychologically and socially **debilitating**.
- It is often difficult to manage, often has comorbidities, and usually requires **specialist referral** in the first instance.
- Good treatment begins with **education of patients**, explaining their disorder and the purpose and means of management.
- Freedom from pain is difficult to achieve: the **objectives** in management, by medical, physical and/or psychological therapies, are reduction of pain intensity and developing patients' coping mechanisms.
- Treatment is **prophylactic**: acute therapies (opioids or other analgesics) have no place in management of PIFP.

Education of patients

A patient information leaflet on persistent idiopathic facial pain is available as Supplementary materials #27.

Key points of information are:

- PIFP is most often a **constant, dull, nagging or aching pain** in the cheek and lower jaw. Rarely there are electric-shock-like pains also.
- There are **no specific triggers**.
- The **causes** are unknown.
- There are **no tests** to confirm the diagnosis.
- **Preventative medications**, taken every day, are the best treatments for most people with PIFP:
    - these medications are more commonly used as antidepressants, but are very useful against chronic pain disorders even in people who are not depressed;
    - **painkillers are unhelpful** and, if taken too often, are likely to make things worse.

Table 2. Drugs used in prophylaxis of persistent idiopathic facial pain

<table>
<tr><td>First line:
• amitriptyline or nortriptyline, 10-100 mg at night</td><td>• intolerance is reduced by starting at a low dose (10 mg) and incrementing by 10-25 mg every 1-2 weeks;
• nortriptyline has fewer anticholinergic side-effects but less good evidence of efficacy</td></tr>
<tr><td colspan="2">Second line (either as monotherapy or as add-on medication):
• gabapentin 600-3600 mg daily
• pregabalin 150-600 mg daily</td></tr>
</table>

Preventative medications

Drugs with some efficacy are shown in Table 2. **Maximum dosages** may be necessary.

- Use of drugs off-licence rests on individual clinical responsibility.

Principles of prophylaxis

- Patients receiving medication more often used as an antidepressant should be **advised of this, and why**; otherwise, they may default on finding out.
- Dosages should be **up-titrated slowly** until pain relief is achieved or side effects become unacceptable.
- **Combinations** may cause fewer side-effects because lower doses may be required of each drug.

Follow-up

While every patient with PIFP requires specialist initial management, long-term follow-up once stable is appropriate in primary care.
