# Supplementary material for: Aids to management of headache disorders in primary care (2nd edition): on behalf of the European Headache Federation and Lifting The Burden: the Global Campaign against Headache
Source: J Headache Pain. 2019 May 21;20(1):57. doi: 10.1186/s10194-018-0899-2 (PMC6734476; doi:10.1186/s10194-018-0899-2)
Supplement: Supplementary file 14 — Guide to referral: Headache management in primary care: when to refer. (PDF 145 kb) [file 10194_2018_899_MOESM14_ESM.pdf]

EUROPEAN HEADACHE FEDERATION EHF

in collaboration with

Lifting The Burden

European principles of management of headache disorders in primary care (2nd edition)

14. Headache management in primary care: when to refer

Most headache disorders presenting to primary care are migraine, tension-type headache or medication-overuse headache. These, usually, can be and are best managed in primary care.

Reasons for specialist referral

- **Diagnostic uncertainty** after due enquiry.
- Diagnosis of **any of the following**, which are best managed by specialists:
  - migraine with aura including motor weakness;
  - chronic migraine;
  - cluster headache;
  - trigeminal neuralgia;
  - persistent idiopathic facial pain.
- **Suspicion of serious secondary headache**, or of serious pathology where investigation may be necessary and is not available in primary care:
  - **progressively worsening** headache over weeks or longer;
  - headache brought on by **coughing**, **exercise** or **sexual activity;**
  - headache **associated with** any of the following:
    - **postural change** indicative of high or low intracranial pressure;
    - unexplained **fever**;
    - **stiffness of the neck**;
    - unexplained **focal neurological symptoms or signs** or with epileptic seizures;
    - **disorder of consciousness or memory**, or **change in personality**;
    - **weight-loss** or poor general condition;

  - **new headache**:
    - in any patient that is **thunderclap** in nature (intense headache with abrupt or "explosive" onset);
    - that is **daily and persistent from onset** in a patient without a prior history of headache;
    - in a patient **older than 50** years;
    - in a patient with a history of **cancer**;
    - in a patient with a history of **immunodeficiency** (including HIV infection);
    - in a patient with a history of **polymyalgia rheumatica**;
    - in a patient with a family history of **glaucoma**;
  - **unusual migraine aura**, especially:
    - prolonged aura (duration >1 hour);
    - aura featuring brainstem symptoms and/or motor weakness;
    - new aura without headache in a patient older than 50 years and in the absence of a prior history of migraine.
- Persistent **management failure**.
- **Comorbid disorders** requiring specialist management.
