# Supplementary material for: Aids to management of headache disorders in primary care (2nd edition): on behalf of the European Headache Federation and Lifting The Burden: the Global Campaign against Headache
Source: J Headache Pain. 2019 May 21;20(1):57. doi: 10.1186/s10194-018-0899-2 (PMC6734476; doi:10.1186/s10194-018-0899-2)
Supplement: Supplementary file 15 — Instruments and other materials to aid diagnosis and management of headache disorders in primary care: Diagnostic criteria for headache disorders in primary care: The International Classification of Headache Disorders, 3rd edition (ICHD-3) – abbreviated form. (PDF 258 kb) [file 10194_2018_899_MOESM15_ESM.pdf]

Lifting The Burden

in official relations with
the World Health Organization

The Global Campaign against Headache

15. Diagnostic criteria for headache disorders in primary care:

The International Classification of Headache Disorders, 3rd edition (ICHD-3) – abbreviated form

Introduction

Headache disorders are common, and the second highest cause of disability worldwide (after low back pain).

The International Classification of Headache Disorders, 3rd edition (ICHD-3), published by the International Headache Society, is the authoritative catalogue of headache disorders. It describes over 200 distinct headache types, subtypes or subforms, and incorporates explicit diagnostic criteria for each one.

Only a small number of these disorders are important in primary care. The purpose of this diagnostic aid, an adaptation of ICHD-3 specifically for primary care, is to help primary-care physicians recognise and correctly diagnose these. It sets out the diagnostic criteria for the three primary headache disorders (with seven types or subtypes), nine secondary headaches and two facial pains that are most likely to be seen in primary care or are important because they are symptomatic of another serious underlying disorder.

How the system works

This diagnostic aid should be used **as a reference**.

The classification distinguishes between **primary headaches**, which have no other underlying causative disorder, and **secondary headaches**, which are attributed to some other disorder. Onset in close temporal relation to another disorder known to cause headache is therefore a diagnostic criterion for all secondary headaches.

The third section of the classification covers **painful cranial neuropathies and other facial pain**.

All diagnoses are numbered according to their position within the classification hierarchy. In this abbreviated version, numbers are not consecutive because many headaches are not included.

Diagnoses are made by applying the criteria set out in the classification. A diagnosis is confirmed only when **all criteria for that disorder are fulfilled**. However, symptoms may have been modified by treatment, and this possibility should be considered in deciding whether criteria are met.

One patient may simultaneously have **two or more headache disorders**. Each should be separately diagnosed because each may require separate management.

The presence of more than one headache disorder can cause confusion, especially when a patient fails to distinguish between them. When this is suspected, it is recommended that he or she prospectively fills out a diagnostic headache diary, for a month or longer, recording the important characteristics of each headache episode. Diaries not only improve diagnostic accuracy but also allow precise judgment of medication consumption. A diary is available as Supplementary materials #16.

Definitions of common terms

Attack of headache (or pain):
Headache (or pain) that builds up, remains at a certain level for minutes to 72 hours, then wanes until it is gone completely.

Attributed to:
This term in ICHD-3 describes the relationship between a secondary headache and the disorder believed to cause it. It requires fulfilment of criteria establishing an accepted level of evidence of causation.

Close temporal relation:
This term is used to describe the relation between an organic disorder and a secondary headache attributed to it.

Duration of attack:
Time from onset until termination of an attack of headache (or pain) meeting criteria for a particular headache type or subtype. When the patient falls asleep during an attack and wakes up relieved, duration is until time of awakening. When an attack of migraine is successfully relieved by medication but symptoms recur within 48 hours, these may represent a relapse of the same attack or a new attack (see *Frequency of attacks*).

Facial pain:
Pain below the orbitomeatal line, above the neck and anterior to the pinnae.

Fortification spectrum:
Angulated, arcuate and gradually enlarging visual disturbance typical of migrainous aura.

Frequency of attacks:
The rate of occurrence of attacks of headache (or pain) per time period (commonly one month). Successful relief of a migraine attack with medication may be followed by relapse within 48 hours. The IHS *Guidelines for Controlled Trials of Drugs in Migraine, 3rd edition,* recommend as a practical solution, especially in differentiating attacks recorded as diary entries over the previous month, to count as distinct attacks only those that are separated by at least 48 hours headache-free.

Headache:
Pain located in the head, above the orbitomeatal line and/or nuchal ridge.

Headache days:
Number of days during an observed period of time (commonly 1 month) affected by headache for any part or the whole of the day.

Intensity of pain:
Level of pain, usually scored on a four-point numerical rating scale (0-3) equivalent to no, mild, moderate and severe pain, or on a visual analogue scale (commonly 10 cm). It may also be scored on a verbal rating scale expressed in terms of its functional consequence: 0, no pain; 1, mild pain, does not interfere with usual activities; 2, moderate pain, inhibits but does not wholly prevent usual activities; 3, severe pain, prevents all activities.

New headache:
Any type, subtype or subform of headache from which the patient was not previously suffering.

Persistent:
This term, used in the context of certain secondary headaches, describes headache, initially acute and caused by another disorder, that fails to remit within a specified time interval (usually 3 months) after that disorder has resolved.

Phonophobia:
Hypersensitivity to sound, even at normal levels, usually causing avoidance.

Photophobia:
Hypersensitivity to light, even at normal levels, usually causing avoidance.

Pressing/tightening:
Pain of a constant quality, often compared to a tight band around the head.

Primary headache (disorder):
Headache, or a headache disorder, not caused by or attributed to another disorder. It is distinguished from secondary headache disorder.

Pulsating:
Characterized by rhythmic intensifications in time with the heart beat; throbbing.

Scintillation:
Visual hallucinations that are bright and fluctuate in intensity, often at approximately 8-10 cycles/second. They are typical of migraine aura.

Scotoma:
Loss of part(s) of the visual field of one or both eyes. Scotoma may be absolute (no vision) or relative (obscured or reduced vision). In migraine, scotomata are homonymous.

Secondary headache (disorder):
Headache, or a headache disorder, caused by another underlying disorder. In ICHD-3, secondary headaches are *attributed to* the causative disorder. Secondary headaches are distinguished from primary headaches. A secondary headache may have the characteristics of a primary headache but still fulfil criteria for causation by another disorder.

Primary headaches

1. Migraine

Migraine is a common disabling primary headache disorder. In the *Global Burden of Disease Survey 2010* (GBD 2010), it was ranked as the third most prevalent disorder in the world. In GBD 2015, it was ranked third-highest cause of disability worldwide in both males and females under the age of 50 years.

Migraine has two major types. 1.1 *Migraine without aura* is a clinical syndrome characterized by headache with specific features and associated symptoms. 1.2 *Migraine with aura* is primarily characterized by the transient focal neurological symptoms that usually precede but sometimes accompany the headache. Some patients, with either type, also experience a prodromal phase, occurring hours or days before the headache, and/or a postdromal phase following headache resolution. Common prodromal symptoms include fatigue, elated or depressed mood, unusual hunger and cravings for certain foods; postdromal include fatigue, elated or depressed mood and cognitive difficulties.

When a patient fulfils criteria for both these types of migraine, both should be diagnosed.

A third type, 1.3 *Chronic migraine*, is much less common but very highly disabling.

1.1 Migraine without aura

Description:

A recurrent headache disorder manifesting in attacks lasting 4-72 hours. Typical characteristics of the headache are unilateral location, pulsating quality, moderate or severe intensity, aggravation by routine physical activity and association with nausea and/or photophobia and phonophobia.

Diagnostic criteria:

A. At least five attacks fulfilling criteria B-D
B. Headache attacks lasting 4-72 hours (when untreated)[1]
C. Headache has at least two of the following four characteristics:
   1. unilateral location
   2. pulsating quality
   3. moderate or severe pain intensity
   4. aggravation by or causing avoidance of routine physical activity (*eg*, walking or climbing stairs)
D. During headache at least one of the following:
   1. nausea and/or vomiting
   2. photophobia and phonophobia
E. Not better accounted for by another ICHD-3 diagnosis.

Note:

1. In children and adolescents (aged under 18 years), attacks may last 2-72 hours.

1.2 Migraine with aura

Description:

Recurrent attacks, lasting minutes, of unilateral fully-reversible visual, sensory or other central nervous system symptoms that usually develop gradually and are usually followed by headache and associated migraine symptoms.

Diagnostic criteria:

A. At least two attacks fulfilling criteria B and C
B. One or more of the following fully reversible aura symptoms:
   1. visual
   2. sensory
   3. speech and/or language
   4. motor, brainstem and/or retinal[1]
C. At least three of the following six characteristics:
   1. at least one aura symptom spreads gradually over ≥5 minutes
   2. two or more aura symptoms occur in succession
   3. each individual aura symptom lasts 5-60 minutes
   4. at least one aura symptom is unilateral[2]
   5. at least one aura symptom is positive[3]
   6. the aura is accompanied, or followed within 60 minutes, by headache[4]
D. Not better accounted for by another ICHD-3 diagnosis.

Notes:

1. Motor, brainstem and retinal symptoms are atypical, occurring in specific subtypes of migraine with aura, and should lead to referral.
2. Aphasia is regarded as a unilateral symptom.
3. Scintillations and pins and needles are positive symptoms of aura.
4. *Typical aura without headache* is a recognised subtype but, in the absence of headache, the diagnosis of aura and its **distinction from mimics that may signal serious disease** (*eg*, transient ischaemic attack) becomes more difficult and often requires investigation.

1.3 Chronic migraine

Description:

Headache occurring on 15 or more days/month for more than 3 months, which, on at least 8 days/month, has the features of migraine headache.

Diagnostic criteria:

A. Headache (migraine-like or tension-type-like[1]) on ≥15 days/month for >3 months, and fulfilling criteria B and C
B. Occurring in a patient who has had at least five attacks fulfilling criteria B-D for 1.1 *Migraine without aura* and/or criteria B and C for 1.2 *Migraine with aura*

C. On ≥8 days/month for >3 months, fulfilling any of the following[2]:
   1. criteria C and D for 1.1 *Migraine without aura*
   2. criteria B and C for 1.2 *Migraine with aura*
   3. believed by the patient to be migraine at onset and relieved by a triptan or ergot derivative

D. Not better accounted for by another ICHD-3 diagnosis[3;4].

Notes:

1. It is impossible to distinguish the individual episodes of headache in patients with such frequent or continuous headaches. In this situation, attacks with and those without aura are both counted in diagnosing 1.3 *Chronic migraine*, as are both migraine-like and tension-type-like headaches.
2. Characterization of frequently recurring headache generally requires a headache diary to record information on pain and associated symptoms day-by-day for at least one month.
3. Because tension-type-like headache is within the diagnostic criteria for 1.3 *Chronic migraine*, this diagnosis excludes the diagnosis of 2. *Tension-type headache* or its types.
4. The most common cause of symptoms suggestive of chronic migraine is medication overuse, as defined under 8.2 *Medication-overuse headache*. Around 50% of patients apparently with 1.3 *Chronic migraine* revert to an episodic migraine type after drug withdrawal; such patients are in a sense wrongly diagnosed as 1.3 *Chronic migraine*. Equally, many patients apparently overusing medication do not improve after drug withdrawal; the diagnosis of 8.2 *Medication-overuse headache* may be inappropriate for these. Therefore, patients meeting criteria for 1.3 *Chronic migraine* and for 8.2 *Medication-overuse headache* should be coded for both. After drug withdrawal, migraine will either revert to an episodic type or remain chronic, and should be re-diagnosed accordingly; either diagnosis may be rescinded.

2. Tension-type headache

This is the most common headache. In the *Global Burden of Disease Survey 2010* (GBD 2010), it was ranked as the second most prevalent disorder in the world (behind dental caries). Two types are important.

2.2 Frequent episodic tension-type headache

Description:

Frequent episodes of headache, typically bilateral, pressing or tightening in quality and of mild to moderate intensity, lasting minutes to days. The pain lacks the specific characteristics of migraine: it does not worsen with routine physical activity and is not associated with nausea, although either photophobia or phonophobia may be present.

Diagnostic criteria:

A. At least 10 episodes of headache occurring on 1-14 days/month on average for >3 months (≥12 and <180 days/year) and fulfilling criteria B-D
B. Lasting from 30 minutes to 7 days
C. At least two of the following four characteristics:
   1. bilateral location
   2. pressing or tightening (non-pulsating) quality
   3. mild or moderate intensity
   4. not aggravated by routine physical activity such as walking or climbing stairs
D. Both of the following:
   1. no nausea or vomiting
   2. no more than one of photophobia or phonophobia
E. Not better accounted for by another ICHD-3 diagnosis[1].

Note:

1. 2.2 *Frequent tension-type headache* often coexists with 1.1 *Migraine without aura*, in which case both diagnoses should be given. A diagnostic headache diary may be required to separate them.

2.3 Chronic tension-type headache

Description:

A disorder evolving from frequent episodic tension-type headache, with daily or very frequent episodes of headache, typically bilateral, pressing or tightening in quality and of mild to moderate intensity, lasting hours to days, or unremitting. The pain does not worsen with routine physical activity, but may be associated with mild nausea, photophobia or phonophobia.

Diagnostic criteria:

A. Headache occurring on ≥15 days/month on average for >3 months (≥180 days/year), fulfilling criteria B-D
B. Lasting hours to days, or unremitting
C. At least two of the following four characteristics:
   1. bilateral location
   2. pressing or tightening (non-pulsating) quality
   3. mild or moderate intensity
   4. not aggravated by routine physical activity such as walking or climbing stairs
D. Both of the following:
   1. no more than one of photophobia, phonophobia or mild nausea
   2. neither moderate or severe nausea nor vomiting
E. Not better accounted for by another ICHD-3 diagnosis[1;2].

Notes:

1. Both 2.3 *Chronic tension-type headache* and 1.3 *Chronic migraine* require headache on 15 or more days/month. For 2.3 *Chronic tension-type headache*,

headache must, on at least 15 days, meet criteria B-D for 2.2 *Frequent episodic tension-type headache*; for *1.3 Chronic migraine* headache must, on at least eight days, meet criteria B-D for 1.1 *Migraine without aura*. A patient can therefore fulfil all criteria for both these diagnoses, for example by having headache on 25 days/month meeting migraine criteria on eight days and tension-type headache criteria on 17 days. In these cases, only the diagnosis 1.3 *Chronic migraine* should be given.

2. In many uncertain cases there is overuse of medication. When this fulfils criterion B for any of the subtypes of 8.2 *Medication-overuse headache* and the criteria for 2.3 *Chronic tension-type headache* are also fulfilled, both disorders should be diagnosed. After drug withdrawal, there may be reversion to episodic tension-type headache. When the disorder remains chronic after withdrawal, the diagnosis of 8.2 *Medication-overuse headache* may be rescinded.

3. Trigeminal autonomic cephalalgias

This group of uncommon disorders shares the clinical features of short-duration headache and prominent cranial parasympathetic autonomic features. Only one, with a prevalence of one per 1,000 in males and lower in females, is expected to be seen and diagnosed in primary care. The others are even rarer and, if seen, may be mistaken for it. All should be referred for specialist management in the first instance.

3.1 Cluster headache

Description:

Attacks of severe, strictly unilateral pain which is orbital, supraorbital, temporal or in any combination of these sites, lasting 15-180 minutes and occurring from once every other day to eight times a day. The pain is associated with ipsilateral conjunctival injection, lacrimation, nasal congestion, rhinorrhoea, forehead and facial sweating, miosis, ptosis and/or eyelid oedema, and/or with restlessness or agitation.

Diagnostic criteria:

A. At least five attacks fulfilling criteria B-D
B. Severe or very severe unilateral orbital, supraorbital and/or temporal pain lasting 15-180 minutes (when untreated)
C. Either or both of the following:
    1. at least one of the following symptoms or signs, ipsilateral to the headache:
        a) conjunctival injection and/or lacrimation
        b) nasal congestion and/or rhinorrhoea
        c) eyelid oedema
        d) forehead and facial sweating
        e) miosis and/or ptosis
    2. a sense of restlessness or agitation
D. Occurring with a frequency between one every other day and 8 per day
E. Not better accounted for by another ICHD-3 diagnosis.

Two subtypes are important.

3.1.1 Episodic cluster headache

Description:

Cluster headache attacks occurring in periods lasting from 7 days to one year, separated by pain-free periods lasting at least 3 months.

Diagnostic criteria:

A. Attacks fulfilling criteria for 3.1 *Cluster headache* and occurring in bouts (cluster periods)
B. At least two cluster periods lasting from 7 days to 1 year (when untreated) and separated by pain-free remission periods of ≥3 months.

3.1.2 Chronic cluster headache

Description:

Cluster headache attacks occurring for one year or longer without remission, or with remission periods lasting less than 3 months.

Diagnostic criteria:

A. Attacks fulfilling criteria for 3.1 *Cluster headache*, and criterion B below
B. Occurring without a remission period, or with remissions lasting <3 months, for at least 1 year.

Secondary headaches

Secondary headache disorders have another causative disorder underlying them; therefore, the headache occurs in close temporal relation to the other disorder, and/or worsens or improves in parallel with worsening or improvement of that disorder. These associations are keys to their diagnosis.

General diagnostic criteria for secondary headaches:

A. Any headache fulfilling criterion C
B. Another disorder scientifically documented to be able to cause headache has been diagnosed[1,2]
C. Evidence of causation demonstrated by at least two of the following:
    1. headache has developed in temporal relation to the onset of the presumed causative disorder
    2. either or both of the following:
        a) headache has significantly worsened in parallel with worsening of the presumed causative disorder
        b) headache has significantly improved in parallel with improvement of the presumed causative disorder
    3. headache has characteristics typical for the causative disorder
    4. other evidence exists of causation
D. Not better accounted for by another ICHD-3 diagnosis.

Notes:

1. The diagnostic criteria for secondary headache disorders **do not set out criteria for diagnosing the underlying disorder**.
2. This criterion may require tests or procedures that cannot be undertaken in primary care. In such cases, the diagnosis cannot be confirmed in primary care. The crucial role of primary care is to recognise the possibility of the diagnosis.

The secondary headaches described below are those that are common or otherwise important (must not be missed) in primary care.

5. Headache attributed to trauma or injury to the head and/or neck

5.2 Persistent headache attributed to traumatic injury to the head

Persistent post-traumatic headache is often part of the post-traumatic syndrome, which includes symptoms such as equilibrium disturbance, poor concentration, decreased work ability, irritability, depressive mood and sleep disturbances.

Description:

Headache of more than 3 months’ duration caused by traumatic injury to the head.

Diagnostic criteria:

A. Any headache fulfilling criteria C and D
B. Traumatic injury to the head has occurred
C. Headache is reported to have developed within 7 days after one of the following:
   1. the injury to the head
   2. regaining of consciousness following the injury to the head
   3. discontinuation of medication(s) impairing ability to sense or report headache following the injury to the head
D. Headache persists for >3 months after its onset
E. Not better accounted for by another ICHD-3 diagnosis[1].

Note:

1. When headache following head injury becomes persistent, the possibility of 8.2 *Medication-overuse headache* needs to be considered.

6. Headache attributed to cranial and/or cervical vascular disorder

6.2.2 Acute headache attributed to non-traumatic subarachnoid haemorrhage

Non-traumatic subarachnoid haemorrhage (SAH) is one of the most common causes of persistent, intense and incapacitating headache of abrupt onset (thunderclap

headache). It is a serious condition, and delayed diagnosis often has a catastrophic outcome: mortality is 40-50%, with 10-20% of patients dying before arriving at hospital; 50% of survivors are left disabled.

Description:

Headache caused by non-traumatic SAH, typically severe and sudden in onset, peaking in seconds (thunderclap headache) or minutes. It can be the sole symptom of non-traumatic SAH.

Diagnostic criteria:

A. Any new headache fulfilling criteria C and D
B. SAH in the absence of head trauma has been diagnosed
C. Evidence of causation demonstrated by at least two of the following:
   1. headache has developed in close temporal relation to other symptoms and/or clinical signs of SAH, or has led to the diagnosis of SAH
   2. headache has significantly improved in parallel with stabilization or improvement of other symptoms or clinical or radiological signs of SAH
   3. headache has sudden or thunderclap onset
D. Either of the following:
   1. headache has resolved within 3 months
   2. headache has not yet resolved but 3 months have not yet passed[1]
E. Not better accounted for by another ICHD-3 diagnosis.

6.4.1 Headache attributed to giant cell arteritis

Giant cell arteritis (GCA) is conspicuously associated with headache, but its characteristics are variable. GCA must be recognized: any persisting headache with recent onset in a patient over 60 years of age should suggest it. Recent repeated attacks of amaurosis fugax associated with headache are very suggestive of GCA. Blindness is a major risk, but preventable by immediate steroid treatment. The time interval between visual loss in one eye and in the other is usually less than 1 week.

Description:

Headache, with variable features, caused by and symptomatic of GCA. Headache may be the sole symptom of GCA, a disease most conspicuously associated with headache.

Diagnostic criteria:

A. Any new headache fulfilling criterion C
B. GCA has been diagnosed
C. Evidence of causation demonstrated by at least two of the following:
   1. headache has developed in close temporal relation to other symptoms and/or clinical or biological signs of onset of GCA, or has led to its diagnosis
   2. either or both of the following:
      a) headache has significantly worsened in parallel with worsening of GCA
      b) headache has significantly improved or resolved within 3 days of high-dose steroid treatment
   3. headache is associated with scalp tenderness and/or jaw claudication
D. Not better accounted for by another ICHD-3 diagnosis.

7. Headache attributed to non-vascular intracranial disorder

7.2 Headache attributed to low cerebrospinal fluid pressure

Description:

Headache caused by low cerebrospinal fluid (CSF) pressure, usually orthostatic and accompanied by neck pain, tinnitus, changes in hearing, photophophia and/or nausea. It remits after normalization of CSF pressure.

Three subtypes are distinguished by aetiology: following-recent dural puncture, attributed to persistent CSF leakage (CSF fistula) or spontaneous.

Diagnostic criteria:

A. Any headache[1] fulfilling criterion C
B. Either or both of the following:
   1. low CSF pressure (<60 mm CSF)
   2. evidence of CSF leakage on imaging
C. Headache has developed in temporal relation to the low CSF pressure or CSF leakage, or led to its discovery
D. Not better accounted for by another ICHD-3 diagnosis.

Note:

1. 7.2 *Headache attributed to low cerebrospinal fluid pressure* is usually but not invariably orthostatic. Headache that significantly worsens soon after sitting upright or standing and/or improves after lying horizontally is likely to be caused by low CSF pressure, but this cannot be relied upon as a diagnostic criterion.

7.4.1 Headache attributed to intracranial neoplasm

Headache is a common symptom of intracranial tumours, more so in young patients (including children), but it rarely remains the only symptom: neurological deficits and seizures are common.

Description:

Headache caused by one or more space-occupying intracranial tumours.

Diagnostic criteria:

A. Any headache[1] fulfilling criterion C
B. A space-occupying intracranial neoplasm has been demonstrated
C. Evidence of causation demonstrated by at least two of the following:
   1. headache has developed in temporal relation to development of the neoplasm, or led to its discovery

2. either or both of the following:
   a) headache has significantly worsened in parallel with worsening of the neoplasm
   b) headache has significantly improved in temporal relation to successful treatment of the neoplasm
3. headache has at least one of the following four characteristics:
   a) progressive
   b) worse in the morning and/or when lying down
   c) aggravated by Valsalva-like manœuvres
   d) accompanied by nausea and/or vomiting

D. Not better accounted for by another ICHD-3 diagnosis.

Note:

1. There are no pathognomonic features of 7.4.1 *Headache attributed to intracranial neoplasm*, although progression or deterioration is a key feature. The other suggestive symptoms (severe, worse in the morning and associated with nausea and vomiting) are not a classical triad; they are more likely in the context of intracranial hypertension and with posterior fossa tumours. Nevertheless, a history indicating raised intracranial pressure should first suggest intracranial neoplasm.

8. Headache attributed to a substance or its withdrawal

8.1.3 Carbon monoxide-induced headache

Carbon monoxide intoxication is particularly associated with headache, which, at low levels of exposure, may be the only symptom. Usually resulting from open fires or faulty gas boilers in the home, it is not rare in some countries, and likely to present to primary care.

Description:

Headache caused by exposure to carbon monoxide (CO), resolving spontaneously within 72 hours after its elimination.

Dependent on carboxyhaemoglobin level, headache ranges from mild without other symptoms, through moderate and pulsating with irritability, to severe with nausea, vomiting, blurred vision and, ultimately, impaired consciousness.

Diagnostic criteria:

A. Bilateral headache fulfilling criterion C
B. Exposure to CO has occurred
C. Evidence of causation demonstrated by all of the following:
   1. headache has developed within 12 hours of exposure to CO
   2. headache intensity varies with the severity of CO intoxication
   3. headache has resolved within 72 hours of elimination of CO
D. Not better accounted for by another ICHD-3 diagnosis.

8.2 Medication-overuse headache

This disorder occurs in patients chronically overusing medication to treat a prior headache disorder, usually 1. *Migraine* or 2. *Tension-type headache*; both the prior headache and 8.2 *Medication-overuse headache* (MOH) should be diagnosed.

Correct diagnosis of MOH is important because patients will not improve without withdrawal of the offending medication. On the other hand, most patients with MOH improve after withdrawal, as does their responsiveness to preventative treatment.

Description:

Headache occurring on 15 or more days/month in a patient with a pre-existing primary headache and developing as a consequence of regular overuse of acute or symptomatic headache medication for more than 3 months. It usually, but not invariably, resolves after the overuse is stopped.

Diagnostic criteria:

A. Headache occurring on ≥15 days/month in a patient with a pre-existing headache disorder
B. Regular overuse for >3 months of one or more drugs that can be taken for acute and/or symptomatic treatment of headache[1;2]
C. Not better accounted for by another ICHD-3 diagnosis.

Notes:

1. Drugs may be ergotamine, one or more triptans, non-opioid analgesics including paracetamol (acetaminophen), acetylsalicylic acid and other non-steroidal anti-inflammatory drugs (NSAIDs), opioids, combination analgesics (typically containing simple analgesics plus opioids, butalbital and/or caffeine) or any combination of these.
2. Overuse is defined as intake on ≥15 days/month for non-opioid analgesics alone and *in all other cases* as intake on ≥10 days/month.

9. Headache attributed to infection

9.1.1 Headache attributed to bacterial meningitis or meningoencephalitis

Headache is the commonest and may be the first symptom of these infections, which should be suspected whenever headache is associated with fever, altered mental state, focal neurological deficits or generalized seizures.

Description:

Headache of variable duration caused by bacterial meningitis or meningoencephalitis. It may develop with mild flu-like symptoms and is typically acute and associated with neck stiffness, nausea, fever and changes in mental state and/or other neurological symptoms and/or signs.

In most cases, headache resolves with resolution of the infection. Rarely it persists (as the subform 9.1.1.3 *Persistent headache attributed to past bacterial meningitis or meningoencephalitis*) for more than 3 months after resolution of the infection.

Diagnostic criteria:

A. Headache of any duration fulfilling criterion C
B. Bacterial meningitis or meningoencephalitis has been diagnosed
C. Evidence of causation demonstrated by at least two of the following:
   1. headache has developed in temporal relation to the onset of the bacterial meningitis or meningoencephalitis
   2. headache has significantly worsened in parallel with worsening of the bacterial meningitis or meningoencephalitis
   3. headache has significantly improved in parallel with improvement in the bacterial meningitis or meningoencephalitis
   4. headache is either or both of the following:
      a) holocranial
      b) located in the nuchal area and associated with neck stiffness
D. Not better accounted for by another ICHD-3 diagnosis.

11. Headache or facial pain attributed to disorder of the cranium, neck, eyes, ears, nose, sinuses, teeth, mouth or other facial or cervical structure

11.3.1 Headache attributed to acute angle-closure glaucoma

Acute angle-closure glaucoma generally causes eye and/or periorbital pain, visual acuity loss (blurring), conjunctival injection and oedema, nausea and vomiting. As intraocular pressure rises, so does the risk of permanent visual loss. Early diagnosis is essential.

Description:

Headache, usually unilateral, caused by acute angle-closure glaucoma and associated with other symptoms and clinical signs of this disorder (eye and/or periorbital pain, visual acuity loss [blurring], conjunctival injection and oedema, nausea and vomiting).

Diagnostic criteria:

A. Any headache fulfilling criterion C
B. Acute angle-closure glaucoma has been diagnosed, with proof of increased intraocular pressure
C. Evidence of causation demonstrated by at least two of the following:
   1. headache has developed in temporal relation to the onset of the glaucoma
   2. headache has significantly worsened in parallel with progression of the glaucoma
   3. headache has significantly improved or resolved in parallel with improvement in or resolution of the glaucoma
   4. pain location includes the affected eye
D. Not better accounted for by another ICHD-3 diagnosis.

Painful cranial neuropathies and other facial pain

13. Painful lesions of the cranial nerves and other facial pain

13.1.1 Trigeminal neuralgia

The diagnosis of 13.1.1 *Trigeminal neuralgia* must be established clinically. Investigations are designed to identify cause.

Description:

A disorder characterized by recurrent unilateral brief electric shock-like pains, abrupt in onset and termination, limited to the distribution of one or more divisions of the trigeminal nerve and triggered by innocuous stimuli. It may develop without apparent cause or be a result of another disorder. Additionally, there may be concomitant continuous pain of moderate intensity within the distribution(s) of the affected nerve division(s).

Diagnostic criteria:

A. Recurrent paroxysms of unilateral facial pain in the distribution(s) of one or more divisions of the trigeminal nerve, with no radiation beyond, and fulfilling criteria B and C
B. Pain has all of the following characteristics:
   1. lasting from a fraction of a second to 2 minutes[1]
   2. severe intensity[2]
   3. electric shock-like, shooting, stabbing or sharp in quality
C. Precipitated by innocuous stimuli within the affected trigeminal distribution[3]
D. Not better accounted for by another ICHD-3 diagnosis.

Notes:

1. Paroxysms may become more prolonged over time.
2. Pain may become more severe over time.
3. Some attacks may be, or appear to be, spontaneous, but there must be a history or finding of pain provoked by innocuous stimuli to meet this criterion.

13.12 Persistent idiopathic facial pain (PIFP)

Description:

Persistent facial and/or oral pain, with varying presentations but recurring daily for more than 2 hours/day over more than 3 months, in the absence of clinical neurological deficit.

13.12 *Persistent idiopathic facial pain* may be comorbid with other pain conditions such as chronic widespread pain and irritable bowel syndrome. In addition, it presents with high levels of psychiatric comorbidity and psychosocial disability.

Diagnostic criteria:

A. Facial and/or oral pain fulfilling criteria B and C
B. Recurring daily for >2 hours/day for >3 months
C. Pain has both of the following characteristics:
    1. poorly localized, and not following the distribution of a peripheral nerve
    2. dull, aching or nagging quality
D. Clinical neurological examination is normal
E. A dental cause has been excluded by appropriate investigations
F. Not better accounted for by another ICHD-3 diagnosis.
