# Supplementary material for: Aids to management of headache disorders in primary care (2nd edition): on behalf of the European Headache Federation and Lifting The Burden: the Global Campaign against Headache
Source: J Headache Pain. 2019 May 21;20(1):57. doi: 10.1186/s10194-018-0899-2 (PMC6734476; doi:10.1186/s10194-018-0899-2)
Supplement: Supplementary file 16 — Instruments and other materials to aid diagnosis and management of headache disorders in primary care: Diagnostic headache diary. (PDF 193 kb) [file 10194_2018_899_MOESM16_ESM.pdf]

in collaboration with

Lifting The Burden

Aids to management of headache disorders in primary care (2nd edition)

16. Headache diary to aid diagnosis in primary care

Instructions

Filling in this diary on a daily basis will help ensure that your headache is diagnosed correctly and its effects are fully assessed. This will improve your treatment. Please complete it **at the end of each day** of the week as a summary of the headache(s) you had that day.

Insert your full name (first and family names) and the date when you started to keep the diary. The diary lasts for 1 week.

Notes:

Row 1: insert the date of each day of the week.

Row 2: circle the appropriate answer (if you circle "no", the diary is completed for today).

Row 3: enter the time your headache started today in hours and minutes (if it began yesterday and was still there today, draw a left arrow ⟵).

Row 4: enter the time the headache went away (if it is still there when you go to bed for the night, draw a right arrow ⟶ and continue your recording tomorrow).

Row 5: circle the appropriate answer (if the pain started on one side and spread or shifted to the other, circle the side on which it began and also "both").

Row 6: there are many ways of describing headache, and the choices here may not match yours perfectly, but tick the box for the one that fits better ("pulsating/throbbing" means the pain worsens with each heart beat; "pressing/tightening" means it is like a tight band or vice around the head).

Row 7: circle the most appropriate answer, thinking about your headache today overall (not at its very worst).

Row 8: circle "no" if your headache prevented you from doing anything today that you had planned to do.

Row 9: if you answered "no" to the previous question, please estimate how much you actually did today of what you had planned to do (tick the appropriate box).

Row 10: circle the appropriate answer (the question refers to simple exercise such as walking or going upstairs, not hard physical exercise).

Row 11: circle the most appropriate answer (nausea means feeling you are going to be sick).

Row 12: circle the appropriate answer.

Row 13: circle the most appropriate answer (this question refers to ordinary light rather than very bright sunlight; if you tried to avoid the light and preferred a dark room, you should answer either "a little" or "a lot").

Row 14: circle the most appropriate answer (this question refers to ordinary noise levels rather than very loud noises; if you tried to avoid noise and preferred a quiet room, you should answer either "a little" or "a lot").

Row 15: write here anything that you believe may have caused your headache.

Row 16: Write the name(s) and strength(s) (*eg*, 6 mg, 500 mg), of anything you took today for your headache, and the number of pills, tablets, suppositories or injections.

Diagnostic headache diary

Name (first name(s)): ______________________________________ (family name) ______________________________________

Record in this diary for 1 week. Start date: _____/_____/_______ Finish date: _____/_____/_______

Please read **the instructions** carefully. Then complete the following **every** evening at bedtime (where indicated, tick the most appropriate box or circle the most appropriate answer).

Please remember to bring this diary to your next appointment

1. Date (day/month)	/	/	/	/	/	/	/
2. Did you have a headache today?<br>(circle one)<br>(if no, the diary is completed for today)	yes / no	yes / no	yes / no	yes / no	yes / no	yes / no	yes / no
3. At what time today did your headache start?<br>(hours and minutes)<br>(if it began yesterday and was still there today, draw a left arrow ←——)							
4. When did it go away?<br>(hours and minutes)<br>(if it is still there when you go to bed for the night, draw a right arrow ——→)							
5. On which side is/was the headache?<br>(circle one)	right<br>left<br>both	right<br>left<br>both	right<br>left<br>both	right<br>left<br>both	right<br>left<br>both	right<br>left<br>both	right<br>left<br>both

6. Which of these better describes the pain? (tick one box)	pulsating/throbbing	☐	☐	☐	☐	☐	☐	☐
	pressing/tightening	☐	☐	☐	☐	☐	☐	☐
7. How bad has the headache been today? (circle one)		not bad<br>quite bad<br>very bad	not bad<br>quite bad<br>very bad	not bad<br>quite bad<br>very bad	not bad<br>quite bad<br>very bad	not bad<br>quite bad<br>very bad	not bad<br>quite bad<br>very bad	not bad<br>quite bad<br>very bad
8. Were you able to do everything you planned to do today? (circle one) (if "yes", go to question 10)		yes / no	yes / no	yes / no	yes / no	yes / no	yes / no	yes / no
9. If no, about how much did you get done? (tick one box)	more than half	☐	☐	☐	☐	☐	☐	☐
	less than half	☐	☐	☐	☐	☐	☐	☐
	nothing at all	☐	☐	☐	☐	☐	☐	☐
10. Was your headache made worse by simple exercise, such as going upstairs? (circle one)		yes / no	yes / no	yes / no	yes / no	yes / no	yes / no	yes / no
11. Have you felt sick (nauseated) today with the headache? (circle one)		no<br>a little<br>a lot	no<br>a little<br>a lot	no<br>a little<br>a lot	no<br>a little<br>a lot	no<br>a little<br>a lot	no<br>a little<br>a lot	no<br>a little<br>a lot
12. Have you been sick (vomited) today? (circle one)		yes / no	yes / no	yes / no	yes / no	yes / no	yes / no	yes / no
13. Has the light bothered you, or made the headache worse today? (circle one)		no<br>a little<br>a lot	no<br>a little<br>a lot	no<br>a little<br>a lot	no<br>a little<br>a lot	no<br>a little<br>a lot	no<br>a little<br>a lot	no<br>a little<br>a lot

14. Has noise bothered you, or made the headache worse today? (circle one)		no<br>a little<br>a lot	no<br>a little<br>a lot	no<br>a little<br>a lot	no<br>a little<br>a lot	no<br>a little<br>a lot	no<br>a little<br>a lot	no<br>a little<br>a lot
15. Did anything trigger the headache? (if so, please write in what it was)								
16. Did you take anything today for the headache (pills, tablets, suppositories, injections or other medicines)?	Name and strength							
	How many?							
	Name and strength							
	How many?							
	Name and strength							
	How many?							
	Name and strength							
	How many?							
	Name and strength							
	How many?							
