# Supplementary material for: Aids to management of headache disorders in primary care (2nd edition): on behalf of the European Headache Federation and Lifting The Burden: the Global Campaign against Headache
Source: J Headache Pain. 2019 May 21;20(1):57. doi: 10.1186/s10194-018-0899-2 (PMC6734476; doi:10.1186/s10194-018-0899-2)
Supplement: Supplementary file 17 — Instruments and other materials to aid diagnosis and management of headache disorders in primary care: Headache calendar for follow-up. (PDF 155 kb) [file 10194_2018_899_MOESM17_ESM.pdf]

in collaboration with

Lifting The Burden

Aids to management of headache disorders in primary care (2nd edition)

17. Headache calendar to aid follow-up in primary care

Instructions

Filling in this calendar on a regular basis will help ensure that your headaches are well treated. Please enter details **every time you have a headache**.

Insert your name and date of birth, and list any medications you take to treat your headache (symptomatic drugs), any you take regularly to prevent your headaches (daily prophylactic drugs), any hormonal medication (such as the contraceptive pill or hormone replacement therapy) and any other regular medication.

Also enter the year in which you start the calendar. It lasts for up to 1 year, but you should begin a new one next January.

Notes:

Every day of each month has a blank box. If you have a headache (migraine or other headache) on any date, record it using the following symbols:

X = migraine **/ = any other type of headache**

Women can record days of menstrual periods or spotting with these symbols:

O = period **o = spotting**

Please use additional symbols or colours to record any other information you feel may be useful.

Headache calendar for follow-up

YEAR:

NAME: DOB:

Symptomatic drugs:

Daily prophylactic drugs:

Hormones:

Other regular medication:

	1	2	3	4	5	6	7	8	9	10	11	12	13	14	15	16	17	18	19	20	21	22	23	24	25	26	27	28	29	30	31
January																															
February																															
March																															
April																															
May																															
June																															
July																															
August																															
September																															
October																															
November																															
December																															

X = migraine / = headache

© Anne MacGregor
