# Supplementary material for: Aids to management of headache disorders in primary care (2nd edition): on behalf of the European Headache Federation and Lifting The Burden: the Global Campaign against Headache
Source: J Headache Pain. 2019 May 21;20(1):57. doi: 10.1186/s10194-018-0899-2 (PMC6734476; doi:10.1186/s10194-018-0899-2)
Supplement: Supplementary file 18 — Instruments and other materials to aid diagnosis and management of headache disorders in primary care: The HALT-90 Index. (PDF 287 kb) [file 10194_2018_899_MOESM18_ESM.pdf]

© *Lifting The Burden*

Lifting The Burden

in official relations with
the World Health Organization

The Global Campaign against Headache

18. HALT-90 Index*

(Headache-Attributed Lost Time – 90 days)

You may be given this short questionnaire before you start treatment.

Your answers to the five simple questions will help us understand how much your headaches are affecting your life, and guide your treatment.

Please answer these five questions carefully

1 On how many **days** in the **last three months** could you **not go** to work or school because of your headaches? ☐☐

2 On how many **days** in the **last three months** could you do **less than half** your usual amount in your job or schoolwork because of your headaches?
(Do not include days you counted in question 1 where you missed work or school.) ☐☐

3 On how many **days** in the **last three months** could you **not do any** household work because of your headaches?
(Do not include days you counted in questions 1 or 2.) ☐☐

4 On how many **days** in the **last three months** could you do **less than half** your usual amount of household work because of your headaches?
(Do not include days you counted in any of the previous questions.) ☐☐

5 On how many **days** in the **last three months** did you **miss** family, social or leisure activities because of your headaches? ☐☐

Grading (I-IV indicate, in order, increasing need for medical care; either III or IV indicates high need)

**0-5**	Minimal or infrequent impact	Grade I
**6-10**	Mild or infrequent impact	Grade II
**11-20**	Moderate impact	Grade III
**20+**	Severe impact	Grade IV

TOTAL ☐☐☐

* HALT is closely based on the first five questions of MIDAS, developed by RB Lipton and WF Stewart.
