# Supplementary material for: Aids to management of headache disorders in primary care (2nd edition): on behalf of the European Headache Federation and Lifting The Burden: the Global Campaign against Headache
Source: J Headache Pain. 2019 May 21;20(1):57. doi: 10.1186/s10194-018-0899-2 (PMC6734476; doi:10.1186/s10194-018-0899-2)
Supplement: Supplementary file 19 — Instruments and other materials to aid diagnosis and management of headache disorders in primary care: The HALT-30 Index. (PDF 283 kb) [file 10194_2018_899_MOESM19_ESM.pdf]

© *Lifting The Burden*

Lifting The Burden

in official relations with
the World Health Organization

The Global Campaign against Headache

19. HALT-30 Index*

(Headache-Attributed Lost Time – 30 days)

Your answers to the five simple questions will help us understand how much your headaches are affecting your life.

Please answer these five questions carefully

**1**	On how many **days** in the **last month** could you **not go** to work or school because of your headaches?	☐☐
**2**	On how many **days** in the **last month** could you do **less than half** your usual amount in your job or schoolwork because of your headaches?<br>(Do not include days you counted in question 1 where you missed work or school.)	☐☐
**3**	On how many **days** in the **last month** could you **not do any** household work because of your headaches?<br>(Do not include days you counted in questions 1 or 2.)	☐☐
**4**	On how many **days** in the **last month** could you do **less than half** your usual amount of household work because of your headaches?<br>(Do not include days you counted in any of the previous questions.)	☐☐
**5**	On how many **days** in the **last month** did you **miss** family, social or leisure activities because of your headaches?	☐☐
	**TOTAL**	☐☐☐

* HALT is closely based on the first five questions of MIDAS, developed by RB Lipton and WF Stewart.
