# Supplementary material for: Aids to management of headache disorders in primary care (2nd edition): on behalf of the European Headache Federation and Lifting The Burden: the Global Campaign against Headache
Source: J Headache Pain. 2019 May 21;20(1):57. doi: 10.1186/s10194-018-0899-2 (PMC6734476; doi:10.1186/s10194-018-0899-2)
Supplement: Supplementary file 20 — Instruments and other materials to aid diagnosis and management of headache disorders in primary care: The HURT questionnaire. (PDF 290 kb) [file 10194_2018_899_MOESM20_ESM.pdf]

Lifting The Burden

in official relations with
the World Health Organization

The Global Campaign against Headache

20. HURT questionnaire

(Headache Under-Response to Treatment)

Your medical treatment for your headaches may not be as good as it can be. By completing this short questionnaire, you will help us improve it.

Please answer these questions carefully

please tick **ONE** box in each row

**1**	On how many **days** in the **last month** did you have a headache?	☐ **none**	☐ **1-2**	☐ **3-5**	☐ **6-15**	☐ **16+**
**2**	On how many **days** in the **last three months** did your headaches make it hard to work, study or carry out household work?	☐ **none**	☐ **1-5**	☐ **6-10**	☐ **11-20**	☐ **21+**
**3**	On how many **days** in the **last three months** did your headaches spoil or prevent your family, social or leisure activities?	☐ **none**	☐ **1-5**	☐ **6-10**	☐ **11-20**	☐ **21+**

Analysis (these questions establish frequency of all headaches and of disabling headaches under current treatment; ticks towards the right suggest increasing need for treatment review)

All ticks in white area	Headache control is good: no review needed.
One or more ticks in lightly-shaded area	Better acute headache management is needed; review Qs 4-8 for guidance; prophylaxis may not be required.
One or more ticks in middle-shaded area	Headache control is not good; review Qs 4-8 to optimise acute medication; consider ways of reducing frequency (trigger avoidance and prophylactic medication).
One or more ticks in dark-shaded area	Disabling headache, poorly treated; possibly chronic daily headache (acute medication should be avoided); review Qs 4-8 and consider ways of reducing frequency.

**4**	On how many **days** in the **last month** did you take medication to **relieve** a headache? (Do not count preventative medication.)	☐ none	☐ 1-4	☐ 5-9	☐ 10-15	☐ 16+
**5**	When you take your headache medication, does one dose get rid of your headache and keep it away?	☐ always	☐ often	☐ sometimes	☐ rarely	☐ never
**6**	Do you feel in control of your headaches?	☐ always	☐ often	☐ sometimes	☐ rarely	☐ never
**7**	Do you **avoid** or **delay** taking your headache medication because you do not like its side-effects?	☐ never	☐ rarely	☐ sometimes	☐ often	☐ always
**8**	What have you been told is your headache diagnosis?<br>Do you feel you understand this diagnosis? (tick one box)	please write your diagnosis here:			☐ yes	☐ no

Analysis (these questions suggest how current management might be improved)

**Q4:** Response should accord with Q1. When medication days are 5-9 there is potential risk of medication overuse. When medication days are >10 there is high risk of medication-overuse headache.	Advise patient about the risk and dangers of medication overuse. Give written information leaflet.<br>Consider ways to reduce frequency (trigger avoidance and prophylactic medication).
**Q5:** Ticks towards the **right** increasingly suggest poor efficacy	Consider treating earlier, changing medication, dose or route of administration, or using combination therapy, according to local guidelines.
**Q6:** This question relates to self-efficacy and to satisfaction.<br>The response should be concordant with previous responses.	When the response is in the shaded area, look for the reason(s) in responses to Qs 1-6. If it is not evident, consider the possibility of co-morbidities.<br>When the response is not concordant, consider cognitive interventions and expectation management.
**Q7:** Ticks towards the **right** increasingly suggest poor tolerability.	Consider changing medication or dose according to local guidelines.
**Q8:** This question relates to education.	Always hand out the appropriate information leaflet.<br>When the diagnosis is wrongly stated, or the answer “no” is given, further explanation may be necessary.
