# Supplementary material for: Aids to management of headache disorders in primary care (2nd edition): on behalf of the European Headache Federation and Lifting The Burden: the Global Campaign against Headache
Source: J Headache Pain. 2019 May 21;20(1):57. doi: 10.1186/s10194-018-0899-2 (PMC6734476; doi:10.1186/s10194-018-0899-2)
Supplement: Supplementary file 21 — Patient information leaflets to aid headache management in primary care (2nd edition): Migraine. (PDF 171 kb) [file 10194_2018_899_MOESM21_ESM.pdf]

Lifting The Burden

in official relations with
the World Health Organization

The Global Campaign against Headache

21. Information for people affected by migraine

(version 2: 2018)

Headache disorders are real – they are not just in the mind. If headache bothers you, it needs medical attention.

The purpose of this leaflet is to help you understand your headache, your diagnosis and your treatment, and to work with your doctor or nurse in a way that will get best results for you.

"When I get migraine it makes me very ill and really puts me out of action for the entire day or more. I can feel one coming on and then the full blown migraine emerges and I feel wretched and need to go to bed. As it progresses, I feel sick and eventually I am sick and then, finally, the pain begins to lessen. The next day I'm left with a vague pain and feel absolutely exhausted but otherwise okay."

What is migraine?

Migraine is a medical disorder.

In most cases it takes place in attacks, once or twice a year in some people but up to several times a month in others. The main feature of these attacks is headache, which may be severe. Other common features are feeling sick (nauseated) or being sick (vomiting) and finding light and noise uncomfortable.

In a few people, these symptoms are very frequent, with headache on more days than not. This is called *chronic migraine*.

If you feel nauseated when you have a headache, or light and noise bother you, or if your headache makes it difficult to carry out your usual daily activities, it is quite likely that it is migraine.

What causes migraine?

Migraine comes from the brain. It is a disorder of the parts of the brain that process pain and other sensations. You probably inherited it from one or other parent or from a grandparent.

Who gets migraine?

About one in six adults have migraine, so it is very common. Women are up to three times more likely to be affected than men. It often starts in childhood or adolescence. In girls in particular it may start at puberty. Because of inheritance, migraine tends to run in families.

What are the different types of migraine?

The commonest is *migraine without aura* (aura is described later). About three-quarters of people with migraine have only this type; one in 10 have *migraine with aura*, and twice this many have both types from time to time. Much less common are attacks of aura alone, with no headache. This type of migraine tends to develop in older people.

There are other types of migraine, including chronic migraine, which are much less common and usually require referral to a specialist.

What are the symptoms of migraine?

Symptoms are present during the attack, which has four stages, although not all of these always happen. Between attacks, most people with migraine are completely well.

The *prodromal phase* comes before any other symptoms of the attack. Only half of people with migraine are aware of this phase. If you are one of these, you may feel irritable, depressed or tired for hours or even one or two days before the headache begins. However, some people find they are unusually energetic during this time. Some have food cravings. Others "just know" that a migraine attack is about to start.

The *aura*, when it happens, is almost always the next phase. Only a third of people with migraine ever have aura, and it may not be part of every attack even for them. Aura is a signal from the brain, which is being temporarily (but not seriously) affected by the migraine process. It lasts, usually, for 10 to 30 minutes, but can be longer. It mostly affects vision. You may notice blank patches, bright or flashing lights or coloured zigzag lines spreading in front of your eyes, usually to one side. Less common are *sensory* symptoms – pins-and-needles or numbness – which generally start in the fingers of one hand, and spread up the arm to affect that side of the face or tongue. When these happen, there are nearly always visual symptoms as well. Difficulty speaking or finding the right words can also be part of the aura.

The *headache phase* is the most troublesome for most people, lasting for a few hours or up to two or three days. Migraine headache is often severe. It tends to be one-sided, but can be on both sides, and although most commonly at the front or in the temple it can be anywhere in the head. It is usually a throbbing or pounding headache, very often made worse by movement. You probably feel nauseated, and may vomit (which seems to relieve the headache). You may also find light and noise unpleasant and prefer to be alone in the dark and quiet. These symptoms can make it difficult for you to continue your usual daily activities during an attack.

The *resolution phase* follows as the headache fades. During this time you may again feel tired, irritable and depressed, and have difficulty concentrating. It can take a further day before you feel fully recovered.

What is my “migraine threshold”?

Migraine is unpredictable. An attack can start at any time. However, some people are more prone to attacks than others. The higher your *migraine threshold*, the less likely you are to develop an attack, and the lower your threshold the more at risk you are.

So-called *triggers* play a part in this. A trigger will set off an attack (although we do not understand how this happens). It does this more easily if your migraine threshold is low. If your threshold is high, two or three triggers may need to come together for this to happen.

Separate from triggers are *predisposing factors*. These have the effect of lowering your threshold, so that triggers work more easily. Tiredness, anxiety and general stress have this effect, as can pregnancy and the menopause in women.

What are the triggers?

Everyone wants to know what might trigger his or her migraine. This is often difficult and sometimes impossible to pin down because triggers are not the same for everybody, or even always the same for different attacks in the same person. Many people with migraine cannot identify any triggers. Possible triggers are many and varied.

Diet: some foods (and alcohol), but only in some people; more commonly, delayed or missed or inadequate meals, caffeine withdrawal and becoming dehydrated.
Sleep: changes in sleep patterns, both lack of sleep and sleeping in.
Other life-style: intense exercise, or long-distance travel, especially across time zones.
Environmental: bright or flickering lights, using a computer, strong smells and marked weather changes.
Psychological: emotional upset or, surprisingly, relaxation after a stressful period.
Hormonal factors in women: menstruation; breaks in combined hormonal contraception or hormone-replacement therapy (HRT).

The commonest trigger is hunger, or not enough food in relation to needs. This is particularly the case in young people – children prone to migraine should never miss breakfast. In women, hormonal changes associated with the menstrual cycle are important potential triggers.

These, and most other triggers, represent some form of *stress*, and suggest that people with migraine do not respond well to change.

What treatment can I take?

Medications that treat the migraine attack are called *acute* treatments. The right ones can be very helpful, but need to be taken correctly and not overused. They include over-the-counter painkillers, most of which contain aspirin, ibuprofen or paracetamol. Of these, paracetamol is least helpful for most people. In all cases, quickly dissolving forms (tablets or powders) work faster and better. Medications containing codeine are not recommended.

You can also take medicine called an anti-sickness treatment (antiemetic) if you feel nauseated or likely to vomit. Some antiemetics actually help the painkillers by

causing your body to absorb them more quickly. You can have these as suppositories if you feel very nauseated during migraine attacks.

Your pharmacist may offer advice on the best over-the-counter treatments to take. If none of these works for you, or you need more than the recommended dose or find you have to take them more than twice a week, you should ask for medical advice.

Your doctor may prescribe one of the specific antimigraine treatments when painkillers and antiemetics do not relieve your symptoms and get you back into action reasonably quickly. They work quite differently. They do not tackle pain but undo what is happening in your brain to cause an attack. They include a group of drugs called triptans. If your doctor advises it, you can use these together with painkillers, antiemetics or both.

There are some simple measures that can make medication more effective.

Take medication early …

Always carry at least one dose of the medication that has been recommended by your doctor, nurse or pharmacist. Take it as soon as you know an attack is coming on. Medication taken early is more likely to work well. During a migraine attack the stomach is less active, so tablets taken by mouth are not absorbed as well into the bloodstream as they would be normally.

… but not too often

Always carefully follow the instructions that come with your medication. In particular, do not take acute treatment too often because you can give yourself a headache from the treatment. This is called *medication-overuse headache*, and there is a separate leaflet on it which you can ask for if you are worried about it. To avoid this happening, do not take medication to treat headache symptoms *regularly* on more than two days a week.

What if these don’t work?

If frequent or severe attacks are not well controlled with acute treatment, so-called *prophylactic* medication is an option. Unlike acute treatment, you *should* take this daily because it works in a wholly different way – by preventing the migraine process starting. In other words, it raises your migraine threshold.

Your doctor or nurse can give you advice on the choice of medicines available and their likely side-effects. Most were first developed for quite different conditions, so do not be surprised if you are offered a medication described as treatment for high blood pressure, epilepsy or even depression. This is *not* why you are taking it. These medications work against migraine too.

If you are taking one of these, do follow the instructions carefully. Research has shown that a very common reason for this type of medication not working is that patients forget to take it.

What else can I do to help myself?

Exercising regularly and keeping fit will benefit you. Avoiding predisposing and trigger factors is sensible, so you should be aware of the full range of possible triggers. You may be able to avoid some triggers even if there are others that you find difficult or impossible to control.

Keep a diary

A diary can record a lot of relevant information about your headaches – how often you get them, when they happen, how long they last and what your symptoms are. They are valuable in helping with diagnosis, identifying trigger factors and assessing how well treatments work.

What if I think I may be pregnant?

You will need advice from your doctor or nurse. Some of the medications used for migraine are unsuitable if you are pregnant.

Do I need any tests?

Most cases of migraine are easy to recognize. There are no tests to confirm the diagnosis, which is based on your description of your headaches and the lack of any abnormal findings when your doctor examines you. A brain scan is unlikely to help.

If your doctor is at all unsure about the diagnosis, he or she may ask for tests to rule out other causes of headaches, but these are not often needed.

Will my migraine get better?

Although there is no cure yet for migraine, treatments can make a real difference for most people. In addition, migraine nearly always becomes less troublesome in later life.

Meanwhile, doing all you can to follow the advice in this leaflet can make the change from a condition that is out of control to one that you can control.

For more information, visit www.l-t-b.org
