# Supplementary material for: Aids to management of headache disorders in primary care (2nd edition): on behalf of the European Headache Federation and Lifting The Burden: the Global Campaign against Headache
Source: J Headache Pain. 2019 May 21;20(1):57. doi: 10.1186/s10194-018-0899-2 (PMC6734476; doi:10.1186/s10194-018-0899-2)
Supplement: Supplementary file 22 — Patient information leaflets to aid headache management in primary care (2nd edition): Tension-type headache. (PDF 162 kb) [file 10194_2018_899_MOESM22_ESM.pdf]

Lifting The Burden

in official relations with
the World Health Organization

The Global Campaign against Headache

22. Information for people affected by tension-type headache

(version 2: 2018)

Headache disorders are real – they are not just in the mind. If headache bothers you, it needs medical attention.

The purpose of this leaflet is to help you understand your headache, your diagnosis and your treatment, and to work with your doctor or nurse in a way that will get best results for you.

"I get headaches that last for several days at a time and feel as though I'm wearing a hat that's too tight - more like a pressure than a real pain. It doesn't really stop me doing anything, but it's much harder to get through the day."

What is tension-type headache?

Tension-type headache is the common sort of headache that nearly everyone has occasionally. Although never serious, it can make it difficult to carry on entirely as normal. In a few people, it becomes bothersome enough to need medical attention, usually because it has become frequent.

What causes tension-type headache?

Tension-type headache is generally thought of as a headache affecting or arising from muscles and their connections. Its causes appear to be many and varied. However, there are some factors that are more important than others:

> **emotional tension:** this can be anxiety, or stress;
>
> **physical tension** in the muscles of the scalp and neck: this may be caused by poor posture, for example when working at a computer, or by lifting a heavy object incorrectly.

Who gets tension-type headache?

Tension-type headache affects most people from time to time, but women more than men. It also affects children.

What are the different types of tension-type headache?

Episodic tension-type headache is often referred to as "normal" or "ordinary" headache. It happens in attacks (*episodes*) that last for anything from half an hour to several days.

The frequency of these varies widely between people, and in individual people over time.

In about three people in every 100, tension-type headache happens on more days than not. This is *chronic tension-type headache*. In some cases, chronic tension-type headache is present all the time – it may ease but never goes completely. This type of headache can be quite disabling and distressing.

What are the symptoms of tension-type headache?

Usually, tension-type headache is described as a squeezing or pressure, like a tight band around the head or a cap that is too tight. It tends to be on both sides of the head, and often spreads down to or up from the neck. The pain is usually moderate or mild, but it can be severe enough to prevent everyday activities. Generally there are no other symptoms, although some people with tension-type headache dislike bright lights or loud noises, and may not feel like eating much.

What can I do to help myself?

These are things you can try.

Relax. Taking a break, having a massage or a warm bath, going for a walk or taking exercise to get you away from the normal routine may help.

Cope with stress. If you have a stressful job, or are faced with a stressful situation that you cannot avoid, try breathing and relaxation exercises to prevent a possible headache. Mindfulness-based stress reduction can be effective. There are many books and smartphone apps to guide you in these exercises.

Take regular exercise. Tension-type headache is more common in people who do not take much exercise than in those who do. Try walking wherever possible, or take stairs rather than the lift, so that exercise becomes a routine part of your life.

Treat depression. If you feel that you are depressed more often than not, it is important to ask for medical advice and get effective treatment.

Keep a diary

A diary can record a lot of relevant information about your headaches – how often you get them, when they happen, how long they last and what your symptoms are. They are valuable in helping with diagnosis, identifying trigger factors and assessing how well treatments work.

Take painkillers if needed …

Simple painkillers such as aspirin or ibuprofen usually work well in episodic tension-type headache. Paracetamol is less effective but suits some people. Medication that contains codeine is not recommended.

… but not too often

Medication only treats the symptoms of tension-type headache. This is perfectly acceptable if you do not get many. To manage frequent headache over the long term, it is better to try to treat the cause.

Always carefully follow the instructions that come with your medication. In particular, do not take painkillers too often because you can give yourself a worse headache from the treatment. This is called *medication-overuse headache*, and a separate leaflet on it is

available if you are worried about it. To avoid this happening, do not take medication to treat headache *regularly* on more than two days a week.

What other treatments are there?

If you have frequent episodic tension-type headache, or more so if you have chronic tension-type headache, painkillers are not the answer. They will only make things worse over time. So-called *prophylactic* medications are an option. Unlike painkillers, you *should* take these every day because they work in a wholly different way. Their purpose is to make you less prone to headache and so prevent headache from even starting.

Your doctor or nurse can advise on the choice of medicines available and their likely side-effects. Most were first developed for quite different conditions, so do not be surprised if you are offered a medication described as treatment for depression or epilepsy, or as a muscle relaxant. This is *not* why you are taking it. These medications work in tension-type headache too, as they do in other painful conditions.

If you are taking one of these, do follow the instructions carefully. Research has shown that a very common reason for this type of medication not working is that patients forget to take it.

Because posture sometimes plays a role in tension-type headache, and because of the muscles involved, your doctor or nurse may suggest physiotherapy to the head and neck. This can help some people greatly.

Other non-drug approaches include relaxation therapy including biofeedback, and acupuncture. These are not suitable for everybody, do not work for everyone, and are not available everywhere. Again, your doctor or nurse will give you advice.

Will these treatments work?

If the cause is identified and treated, episodic tension-type headache rarely continues to be a problem. Very often, it improves on its own, or the cause goes away, and no further treatment is needed.

For some people, especially with chronic tension-type headache, these treatments do not help or only partially help. If all else fails, you may be referred to a pain clinic which uses a wider range of treatments.

Do I need any tests?

There are no tests to confirm the diagnosis of tension-type headache. This is based on your description of the headaches and the lack of any abnormal findings when you are examined. Be sure to describe your symptoms carefully. Also tell your doctor how many painkillers or other medications you are taking for your headaches, and how often you are taking them.

A brain scan is unlikely to help. If your doctor is at all unsure about the diagnosis, he or she may ask for tests to rule out other causes of headaches, but these are not often needed. If your doctor does not ask for any, it means they will not help to give you the best treatment.

For more information, visit www.l-t-b.org
