# Supplementary material for: Aids to management of headache disorders in primary care (2nd edition): on behalf of the European Headache Federation and Lifting The Burden: the Global Campaign against Headache
Source: J Headache Pain. 2019 May 21;20(1):57. doi: 10.1186/s10194-018-0899-2 (PMC6734476; doi:10.1186/s10194-018-0899-2)
Supplement: Supplementary file 23 — Patient information leaflets to aid headache management in primary care (2nd edition): Cluster headache. (PDF 165 kb) [file 10194_2018_899_MOESM23_ESM.pdf]

Lifting The Burden

in official relations with
the World Health Organization

The Global Campaign against Headache

23. Information for people affected by cluster headache

(version 2: 2018)

Headache disorders are real – they are not just in the mind. If headache bothers you, it needs medical attention.

The purpose of this leaflet is to help you understand your headache, your diagnosis and your treatment, and to work with your doctor or nurse in a way that will get best results for you.

"They often wake me during the night, usually a couple of hours after I've gone to bed. The pain builds up in a matter of seconds and is just excruciating. It's only in my right eye, like a red hot poker. I don't know where to put myself. I have to do something to distract from the pain. Sometimes I pace up and down the room holding my head, or just sit in the chair and rock."

What is cluster headache?

Cluster headache is the name given to short-lasting attacks of very severe one-sided head pain, usually in or around the eye. These generally start without warning, one or many times every day, generally at the same times each day or during the night. Quite often, the first one will wake the person up an hour or two after falling asleep.

Cluster headache is sometimes said to be a type of migraine, but this is not so. It is a quite distinct headache and needs different treatment from migraine.

Who gets cluster headache?

Cluster headache is not common. It affects up to three in every 1,000 people. Men are three times more likely than women to have cluster headache, which makes it unusual among headache disorders. The first attack is likely to happen between the ages of 20 and 40, but cluster headache can start at any age, even in childhood.

What are the different types of cluster headache?

Episodic cluster headache is more common. This type happens daily for limited periods (*episodes*) and then stops, a feature giving rise to the term "cluster". Usually these periods last from six to 12 weeks, but they can end after two weeks or go on for anything up to six months. They tend to come at about the same time each year, often spring or autumn, but some people have two or three episodes every year and others have gaps of two or more years between episodes.

In between, people with episodic cluster headache have no symptoms of the condition at all.

Chronic cluster headache, which accounts for about one in five cases of cluster headache, does not stop. Daily or near-daily attacks continue year after year without a break, or with only short breaks of less than a month.

Episodic cluster headache can turn into chronic cluster headache, and *vice versa.*

What are the symptoms of cluster headache?

There are a highly recognizable group of symptoms. Most importantly, cluster headache is excruciatingly painful. The pain is strictly one-sided and always on the same side (although in episodic cluster headache it can switch sides from one episode to another). It is in, around or behind the eye and described as searing, knife-like or boring. It becomes worse very quickly, reaching full force within five to 10 minutes, and when untreated lasts between 15 minutes and three hours (most commonly between 30 and 60 minutes). In marked contrast to migraine, during which most people want to lie down and keep as quiet as possible, cluster headache causes agitation. People with this condition cannot keep still – they will pace around or rock violently backwards and forwards, even going outside.

Also, the eye on the painful side becomes red and waters and the eyelid may droop. The nostril feels blocked, or runs. The other side of the head is completely unaffected.

What causes cluster headache?

Despite a great deal of medical research into the cause of cluster headache, it is still not known. Much interest centres on the timing of attacks, which appears to link to circadian rhythms (the biological clock). Recent research has highlighted changes in a part of the brain known as the hypothalamus, the area that controls the body clock.

What are the triggers?

So-called *triggers* set off a headache attack. Alcohol, even a small amount, may trigger an attack of cluster headache during a cluster episode but not at other times. Certain medications that open blood vessels in order to improve blood flow can also trigger an attack of cluster headache. These include drugs such as Viagra taken to help erection of the penis. Another medication that can trigger an attack of cluster headache is nitroglycerin, used to treat episodes of chest pain in people who have narrowing of the vessels that supply blood to the heart. We do not understand how this happens. There do not appear to be other common trigger factors as in migraine.

Do I need any tests?

Because of its set of symptoms, cluster headache is easy to recognize. There are no tests to confirm the diagnosis, which is based on your description of the headaches and other symptoms and the lack of any abnormal findings when your doctor examines you. Therefore, it is very important to describe your symptoms carefully.

If your doctor is not sure about the diagnosis, a brain scan may be carried out to rule out other causes of headaches. However, this is not often needed. If your doctor does not ask for a brain scan, it means that it will not help to give you the best treatment.

What treatments are there?

There are a number of treatments for cluster headache which often work well. They all need a doctor's prescription. Medications that treat the symptoms of an attack are called *acute* treatments. If frequent or severe attacks are not well controlled with acute treatment, *preventative* medication is an option. Unlike acute treatment, you should take this daily because it works in a wholly different way – by stopping the attack before it begins.

The most usual treatments for the attack are 100% oxygen, which needs a cylinder, high-flow regulator and special mask from a supplier, or an injected drug called sumatriptan, which you can give to yourself using a special injection device.

Preventative medications are the best treatments for most people with cluster headache. You take these every day for the usual length of the cluster episode. They are effective, but you do need rather close medical supervision, often with blood tests or monitoring of the heart, because of the possible side-effects. You may be referred to a specialist for this. The referral should be urgent because, if you have this condition, we know you are suffering greatly.

What if these don't work?

There are a range of preventative medications. If one does not work very well, another may. Sometimes, two or more are used together.

Treatments that use a mild electrical stimulation to treat and prevent pain have also been developed.

What can I do to help myself?

Ordinary painkillers do not work – they take too long, and the headache will usually have run its course before they take effect. For effective treatment, you will need to ask for medical help. Do this *at the start* of a cluster episode, because preventative treatment appears to be more successful when started early in the episode. For chronic cluster headache, you will need to take continuous preventative treatment.

Keep a diary

You can use a diary to record a lot of relevant information about your headaches – how often you get them, when they happen, how long they last and what your symptoms are. They are valuable in helping with diagnosis, identifying trigger factors and assessing how well treatments work.

Will my cluster headache get better?

Cluster headache may return for many years, but then stop of its own accord. About one in ten people with episodic cluster headache develop chronic cluster headache; about one third of people with chronic cluster headache change back to episodic headache. The length of time free from symptoms between periods of cluster headache does lengthen as people get older.

For more information, visit www.l-t-b.org
