# Supplementary material for: Aids to management of headache disorders in primary care (2nd edition): on behalf of the European Headache Federation and Lifting The Burden: the Global Campaign against Headache
Source: J Headache Pain. 2019 May 21;20(1):57. doi: 10.1186/s10194-018-0899-2 (PMC6734476; doi:10.1186/s10194-018-0899-2)
Supplement: Supplementary file 24 — Patient information leaflets to aid headache management in primary care (2nd edition): Medication-overuse headache. (PDF 166 kb) [file 10194_2018_899_MOESM24_ESM.pdf]

Lifting The Burden

in official relations with
the World Health Organization

The Global Campaign against Headache

24. Information for people affected by medication-overuse headache

(version 2: 2018)

Headache disorders are real – they are not just in the mind. If headache bothers you, it needs medical attention.

The purpose of this leaflet is to help you understand your headache, your diagnosis and your treatment, and to work with your doctor or nurse in a way that will get best results for you.

"I began to get headaches which would come on every morning, not long after I woke up. I could carry on working through them but it was hard to concentrate. I found that a couple of painkillers would ease the headache. The headaches started to get more frequent and now I take painkillers most days. My head never seems to clear completely."

What is medication-overuse headache?

Medication-overuse headache (MOH) is a frequent headache *caused by* medication to treat the symptoms of migraine or tension-type headache, when it is taken too often for too long. It can also be caused by taking painkillers too often for other types of headache or other types of pain.

Any medication you use to treat the symptoms of headache can cause MOH. Aspirin, paracetamol, ibuprofen, codeine and opioids – in fact, all painkillers, even those bought over the counter – can give this problem. And it is not just painkillers. Drugs that specifically treat migraine headache also lead to MOH when used too often. These include triptans and ergotamine.

A similar headache (although not strictly MOH) can result from taking too much caffeine. The usual source of this is coffee, tea or cola drinks, but it can come from caffeine tablets or from caffeine included in many painkillers.

The exact way MOH develops is not known, and may be different according to the nature of the medication. Triptans and ergotamine may cause a rebound effect, with headache returning after they wear off. Painkillers are believed to cause, over time, a change in pain-signalling systems in the brain. This means people become used to the effects of the medication so that they need more and more of it.

For most people with occasional headaches, painkillers are a safe and effective treatment. However, MOH may develop in anyone regularly taking simple painkillers on three or more days a week or other headache medications on two or more days a week. Usually, the person with MOH began with occasional attacks of migraine or (less commonly) tension-type headache. For varying reasons, the headaches start happening more often. This may be through natural variation or because an extra headache has developed, perhaps due to stress or muscular pain. The increase in headache leads to use of more and more medication to try to control the symptoms, until eventually both happen every day.

Many people in this situation know that they are taking more medication than is wise, and try to reduce the amount. This leads them to have withdrawal symptoms – worsening headache – for which they take more medication. It is easy to see how this results in a vicious cycle, which can be difficult to break. It makes not so much difference how *much* you take – if you regularly use the full dose of painkillers on one or two days a week only, you are unlikely to develop MOH. However, take just a couple of painkillers on most days and you may well be making your headaches worse. It is *frequent* use over a period of time that causes the problem.

Who gets medication-overuse headache?

It is surprisingly common; one person in 50 or even more may have this problem. It is more common in women than men, and it also happens in children.

What are the symptoms of medication-overuse headache?

The main feature is very frequent headache. This varies, but is often a dull pain with overlapping migraine like attacks. Other common symptoms, alongside headache, are feeling tired, feeling sick, being irritable and difficulty sleeping. Sometimes headache seems relentless, although it may change as you go through the day. MOH is often at its worst early in the morning.

Will my medication-overuse headache get better?

If your headache is caused by overusing medication, then it is very likely to improve once you stop doing this, but not otherwise.

How MOH is treated depends on what was the original headache and what medication is being overused. Getting the treatment right is very important, so medical care is usually necessary. While painkillers or antimigraine treatments may seem to help, this relief is only partial and the effect lessens over time. In fact, these treatments will make the condition worse.

Do I need any tests?

There are no tests to confirm the diagnosis of MOH. This is based on your description of your headaches and the lack of any unusual findings when you are clinically examined. Therefore, it is very important that you carefully describe your symptoms and how they developed. It is also very important that you say how many painkillers or other medications you are taking for your headaches, and how often you are taking them.

Your doctor should be able to tell quite easily whether you have an illness more serious than MOH. If he or she is not sure about the diagnosis, or there is any sudden change in your headache, tests including a brain scan may be carried out to

rule out other causes of your headache. However, these are not often needed. If your doctor does not ask for a brain scan, it means that it will not help to give you the best treatment.

What can I do to help myself?

The ***only*** way of treating this condition is to stop overusing the responsible medication (*withdrawal*). Clinical studies show that at least two out of every three people who do this improve greatly. However, it may take up to two months before you see the full benefit. Even if headaches continue after that time, despite stopping the medication, their cause usually becomes clear and they can then be treated by correctly prescribed specific treatment.

You can withdraw either by stopping in one go or by gradually reducing the amount taken over two to three weeks. Either way, you will almost certainly have withdrawal symptoms – worsening headache, feeling sick, perhaps being sick, anxiety and difficulty sleeping. These symptoms will appear within 48 hours. They may be less severe, but will last longer, if you try to stop slowly.

It makes sense to choose when to withdraw, and not begin shortly before an important event. Do warn your work colleagues that you may be unable to come into work for a few days.

Are there other treatments I can take?

There are medications, which a doctor can prescribe, that you can take every day to help you withdraw. They work only if you stop all other headache medication, and even then it is uncertain how much they help. You will also have to stop these at some point, and, for most people, it is better to do without them.

What if I just carry on as I am?

If medication overuse is causing your frequent headaches, carrying on as you are is not an option. You will continue to have ever-more frequent headaches, which will not be helped by painkillers or preventative medicine. Eventually you may do yourself other harm as well, such as damage to your liver and kidneys.

How can I make sure it doesn’t happen again?

As it develops, MOH largely replaces the original headache (migraine or tension-type headache) for which you took the medication in the first place. This means that, as your MOH improves after withdrawal, you can expect your original pattern of headache to return.

There are separate leaflets on both migraine and tension-type headache. You may find one of these useful at this stage.

If you need to, you can cautiously restart using medication for this headache once the frequency of headache has returned to normal. This is likely to be after several weeks or longer.

Be careful, because there is a risk of following the same path as before. To prevent this, avoid treating headaches on more than three days in a row, or on a *regular* basis on more than two days in a week.

If a headache doesn't get better, or keeps returning, do not just continue taking medication but consult your doctor or nurse.

See your doctor or nurse if frequent headaches do not go, or if they return in the future.

Keep a diary

You can use a diary to record a lot of useful information about your headaches – how often you get them, when they happen, how long they last and what other symptoms you have. Diaries help with diagnosis, recording how often you take medication. For people at risk of MOH, this is especially important. Diaries also help in judging how well treatments work.

For more information, visit www.l-t-b.org
