# Supplementary material for: Aids to management of headache disorders in primary care (2nd edition): on behalf of the European Headache Federation and Lifting The Burden: the Global Campaign against Headache
Source: J Headache Pain. 2019 May 21;20(1):57. doi: 10.1186/s10194-018-0899-2 (PMC6734476; doi:10.1186/s10194-018-0899-2)
Supplement: Supplementary file 25 — Patient information leaflets to aid headache management in primary care (2nd edition): Female hormones and headache. (PDF 160 kb) [file 10194_2018_899_MOESM25_ESM.pdf]

Lifting The Burden

in official relations with
the World Health Organization

The Global Campaign against Headache

25. Information on female hormones for women with headache

(version 2: 2018)

Headache disorders are real – they are not just in the mind. If headache bothers you, it needs medical attention.

The changing pattern of hormones throughout a woman's life, from puberty to the menopause, has an important effect on migraine and other headaches.

Knowing what to expect can help women understand why headaches occur and, importantly, when to seek help.

Given the strong influence of hormones on headache in women, you may wonder why doctors do not do any hormone tests. The simple answer is that no tests are able to show doctors the cause of the problem.

Headache and women's hormones

The relationships between female hormones and the processes that cause headaches, or make them better or worse, are very complex. Even when hormones are clearly a factor in headache problems, all the standard hormone tests are usually normal. Studies measuring hormone levels show no differences between women with headaches triggered by hormonal changes and women without a hormonal trigger.

Headache, migraine and puberty

Puberty is the time when a girl begins to produce hormones in a monthly cycle that leads to the start of menstruation. This is therefore the time when hormones may first influence headaches. Although migraine can start at any age, puberty commonly brings about its onset.

Headaches and the menstrual cycle

Many women notice a link between headaches and their menstrual cycle. Headaches are typically more frequent and more severe in the days around the menstrual period. At the same time, there may be mood changes, water retention and other premenstrual symptoms, which improve as the period starts.

Migraine is also affected by the menstrual cycle, and in some women is triggered by the natural drop in levels of the hormone estrogen, which happens just around the time of the menstrual period. Other hormones that change with the menstrual cycle,

such as prostaglandins, which are released just before and during a period, may also be an important trigger. This is particularly likely in women who get migraine only on the first or second day of bleeding.

So-called "menstrual migraine" can be more severe than headaches at other times of the month, so take your migraine treatments early. If over-the-counter medication is not effective, your doctor can prescribe drugs that may be more helpful. If necessary, you can also consider options to prevent menstrual attacks. No drugs are sold specifically for prevention of menstrual migraine, but there are some that often work well. The choice of drug depends on any other period problems you have, which may also benefit from treatment, so this is something to discuss with your doctor or nurse.

Headaches and contraception

Hormonal contraception, such as combined hormonal contraceptives (pills, patches, rings and injections), is very safe for most women. This is equally true for women with migraine. Many women find that combined hormonal contraceptives have no effect upon their headaches – or even help them. Even so, headaches are a common side-effect of these medications. In most cases, headaches of this sort improve after a few months, and they are rarely a reason to stop contraception.

If you had *migraine without aura* before starting combined hormonal contraceptives, you may notice that you get your attacks during the medication-free interval. The drop in hormone levels causes a "withdrawal" bleed, like a period. However, the drop in estrogen level can also trigger migraine, just as during the natural menstrual cycle.

However, if you have *migraine with aura*, you should *not* take combined hormonal contraceptives. This is because the estrogen in these contraceptives can increase the risk of a stroke. This risk is very low in women under 50, but it is sensible not to increase it when there are many choices of other methods of contraception, and several of these are even more effective contraceptives than the combined hormonal methods.

If you have *migraine without aura*, and this changes to *migraine with aura* after starting combined hormonal contraceptives, you should **stop them immediately** and seek medical advice. You will need to see your doctor or nurse because you may also need emergency contraception.

There is a separate leaflet explaining what migraine without aura and migraine with aura are. Ask your doctor or nurse if you would like to have this.

Progestogen-only methods (pills, implants, injections and intrauterine methods) do not increase the risk of a stroke but have varying effects on headaches. Most evidence suggests that, if the method "switches off" normal periods, headaches usually improve.

Headaches, pregnancy and breastfeeding

Fortunately, most women find that headaches improve during the later part of pregnancy. This is especially likely for *migraine without aura*. The benefit may continue through breastfeeding.

In the first few months of pregnancy, however, headaches may be worse. One reason is that sickness, particularly when it is severe, can reduce food and fluid intake and result in low blood sugar and dehydration. If this happens to you, try to eat small, frequent carbohydrate snacks and drink plenty of liquids. Adequate rest is important to avoid over-tiredness. Other preventative measures that can safely be tried include acupuncture, biofeedback and other relaxation techniques.

Women who have *migraine with aura* before they become pregnant are more likely to continue to have attacks during pregnancy. If migraine happens for the first time during pregnancy, it is likely to be *migraine with aura*.

There is no evidence that headaches or migraine, either with or without aura, have any effect on the pregnancy itself or on the baby's growth. It is, of course, important to make sure that any treatments taken for headaches are safe. Few drugs have been tested for safety in pregnancy and during breastfeeding. In fact, paracetamol (when used correctly) is regarded as the safest medication throughout pregnancy and breastfeeding. Unfortunately, this is not the most effective treatment, especially for migraine, and even paracetamol should not be taken too often. However, there are other medications that can be taken under medical supervision. If you feel you need to take any other drugs for headaches while pregnant, **check with your doctor first**.

Headaches, the menopause and hormone replacement therapy (HRT)

In the years leading up to the menopause, the ovaries produce less and less estrogen. During this time of hormonal imbalance, migraine and other headaches often become more frequent or severe. For most women, they settle again after the menopause, possibly because the hormonal changes stop and estrogen settles at a lower level.

You can decide to take HRT or not regardless of your headaches. Unlike the artificial estrogens in contraceptives, the natural estrogens in HRT do not seem to increase the risk of a stroke in women with migraine with aura. Migraine may worsen with oral HRT but improve with non-oral HRT such as patches or gels. Too high an estrogen dose can trigger migraine aura, in which case you should use a lower dose. Whichever type of HRT you start with, do give it a fair trial; during the first three months, the body needs to become used to the change of hormones.

Headaches and hysterectomy

Hysterectomy *does not help* in the treatment of hormonal headaches. The normal menstrual cycle happens because of actions of several different organs in the body – in the brain as well as the ovaries and the womb. Removing the womb alone has little effect on the hormonal changes of the menstrual cycle, even though periods stop.

What can I do to help myself?

If you think that your headaches are worse with hormonal changes, the first thing to do is to keep a diary of the dates of the first day of each period and the dates of each day of your headache. After a few months, look back over the records to see if there are any patterns. This will tell you if hormones are having an important effect.

Remember to look at the other causes of headaches. Think about other possible trigger factors. These may still be part of the problem even if hormones are too – and you may be able to avoid them.

When a headache starts, particularly migraine, do not delay taking treatment; if you leave it too late, it may be less helpful. If your treatments do not allow you to continue your usual daily activities, take your diary to your doctor and discuss other options.

For more information, visit www.l-t-b.org
