# Supplementary material for: Aids to management of headache disorders in primary care (2nd edition): on behalf of the European Headache Federation and Lifting The Burden: the Global Campaign against Headache
Source: J Headache Pain. 2019 May 21;20(1):57. doi: 10.1186/s10194-018-0899-2 (PMC6734476; doi:10.1186/s10194-018-0899-2)
Supplement: Supplementary file 26 — Patient information leaflets to aid headache management in primary care (2nd edition): Trigeminal neuralgia. (PDF 120 kb) [file 10194_2018_899_MOESM26_ESM.pdf]

Lifting The Burden

in official relations with
the World Health Organization

The Global Campaign against Headache

26. Information for people affected by trigeminal neuralgia

Trigeminal neuralgia is real – it is not just in the mind. If facial pain bothers you, it needs medical attention.

The purpose of this leaflet is to help you understand your pain, your diagnosis and your treatment, and to work with your doctor or nurse in a way that will get best results for you.

What is trigeminal neuralgia?

Trigeminal neuralgia (TN) is an unpleasant disorder affecting a nerve in the face. This results in severe electric-shock-like pains travelling through the nerve.

What causes TN?

Despite a lot of medical research, the exact cause is still not known. In some people, a blood vessel lies very close to the affected nerve and squeezes it. However, there are other unknown causes.

What are the symptoms of TN?

People with TN describe these pains, usually in the cheek or jaw and always on one side only. They are very brief, but happen one after another in bouts for up to two minutes, several times every day for weeks or months, although rarely at night. They usually start without warning, but can be triggered by light touch, wind or cold air on the face, eating, drinking, brushing the teeth or even speaking.

In between attacks there is no pain, but people can be so afraid of triggering attacks that they stop eating or drinking. TN is therefore very debilitating.

Who gets TN?

TN is not common. It affects one or two people in every 1,000. Women are twice as likely as men to have it. The first attacks usually happen above the age of 50 years, but TN can start at any age.

Do I need any tests?

Yes, a brain scan (MRI) is essential. TN is quite easy to recognize in the clinic, but sometimes another, underlying problem can cause very similar symptoms.

What treatments are there?

Painkillers do not help. However, there are **preventative medications** for TN. You should be referred to a specialist for treatment. The referral should be urgent because, if you have this condition, we know you are suffering greatly.

The treatments work by stopping attacks before they begin. You take them every day, and may need to do so for quite some time. High doses may be necessary. You will need rather close medical supervision, often with blood tests or monitoring of the heart, because of the possible side-effects. You may reduce the dose once the attacks subside and are fully under control.

What if these don't work?

There are a range of preventative medications. If one does not work very well, another may. Sometimes, two or more are used together.

Occasionally, surgery is required. It is a last resort because permanent side effects can result. You will need to discuss these with the specialists.

Will my TN get better?

TN can usually be kept under control. Sometimes it stops completely, of its own accord, but this is unpredictable.

What can I do to help myself?

For effective treatment, you will need medical help. People with TN often see their dentist, but **TN does not require dental treatment**. It is important that dental work on healthy teeth is avoided.

Do take preventative medications exactly as instructed. Ordinary painkillers do not work – they take too long. You should avoid taking these often, because they may well make things worse.

You may recognize triggers, and be able to avoid them. When these include eating or drinking, further problems arise if you do not eat or drink enough to stay healthy.

Keep a diary

You can use a diary to record a lot of useful information about your attacks – how often you get them, when they happen, how long they last and what seems to trigger them. This can be very helpful in assessing how well treatments work.
