# Supplementary material for: Aids to management of headache disorders in primary care (2nd edition): on behalf of the European Headache Federation and Lifting The Burden: the Global Campaign against Headache
Source: J Headache Pain. 2019 May 21;20(1):57. doi: 10.1186/s10194-018-0899-2 (PMC6734476; doi:10.1186/s10194-018-0899-2)
Supplement: Supplementary file 27 — Patient information leaflets to aid headache management in primary care (2nd edition): Persistent idiopathic facial pain. (PDF 251 kb) [file 10194_2018_899_MOESM27_ESM.pdf]

Lifting The Burden

in official relations with
the World Health Organization

The Global Campaign against Headache

27. Information for people affected by persistent idiopathic facial pain

Persistent idiopathic facial pain is real – it is not just in the mind. If facial pain bothers you, it needs medical attention.

The purpose of this leaflet is to help you understand your pain, your diagnosis and your treatment, and to work with your doctor or nurse in a way that will get best results for you.

What is persistent idiopathic facial pain?

Persistent idiopathic facial pain (PIFP) is a constant, dull, nagging or aching pain in one side of the face, of unknown cause.

What causes PIFP?

The cause of PIFP is unknown.

What are the symptoms of PIFP?

People with PIFP describe this type of pain, usually in the cheek and lower jaw but sometimes around the ear and temple. Rarely there are electric-shock-like pains also, which can cause confusion with trigeminal neuralgia. The two disorders can overlap.

Unlike trigeminal neuralgia, PIFP has no specific triggers.

Who gets PIFP?

PIFP is very rare. It mostly affects younger women, but can start at any age.

Do I need any tests?

There are no tests to confirm the diagnosis of PIFP.

What treatments are there?

Painkillers do not work in PIFP. **Preventative medications** are the best treatments for most people with this condition. They all need a doctor's prescription, and you should be referred to a specialist for treatment.

The treatments work by stopping attacks before they begin. You take them every day, and may need to do so for quite some time. High doses may be necessary. You

will need rather close medical supervision, often with blood tests or monitoring of the heart, because of the possible side-effects. You may reduce the dose once the attacks come under control.

What if these don't work?

There are a range of preventative medications. If one does not work very well, another may. Sometimes, two or more are used together.

What can I do to help myself?

For effective treatment, you will need to ask for medical help. People with PIFP sometimes see their dentist, but **PIFP does not require dental treatment**. It is important that dental work on healthy teeth is avoided.

Do take preventative medications exactly as instructed. Ordinary painkillers do not work in PIFP, and may well make things worse if taken too often.

Keep a diary

You can use a diary to record a lot of useful information about your pain – how often you get it, when it happens and how long it lasts. This can be very helpful in assessing how well treatments work.
