# Supplementary material for: Aids to management of headache disorders in primary care (2nd edition): on behalf of the European Headache Federation and Lifting The Burden: the Global Campaign against Headache
Source: J Headache Pain. 2019 May 21;20(1):57. doi: 10.1186/s10194-018-0899-2 (PMC6734476; doi:10.1186/s10194-018-0899-2)
Supplement: Supplementary file 28 — Translation, and the preservation of original meaning, of materials developed to improve headache management: Translation protocol for lay documents (2nd edition). (PDF 146 kb) [file 10194_2018_899_MOESM28_ESM.pdf]

Lifting The Burden

in official relations with
the World Health Organization

The Global Campaign against Headache

28. Translation protocol for lay documents

(2nd edition: 2018)

These guidelines have been developed by *Lifting The Burden* (LTB).

They are for the translation of documents (referred to as "lay documents") produced for the Global Campaign against Headache as information for lay people, including people with headache, the general public and the lay media.

Translations of all lay documents for the Global Campaign against Headache should follow these guidelines in order to ensure high-quality translation and be approved by LTB.

Procedure

Translation should follow five steps.

1. Coordination of the translation

A translation coordinator, who oversees but does not carry out the translation, is selected according to the following criteria:

- bilingual in English and the target language (ideally a native speaker and resident of the country of the target language);
- has ability to mediate between different translators and to understand the points of view of lay and professional translators.

If the coordinator is not a native speaker, a referee (native speaker) must be nominated. The referee cannot be involved in the translation process, and is called upon to arbitrate should irreconcilable views amongst translators prevent the production of a consensus-based translation.

The tasks of the coordinator include:

- selecting the translators, assessor and review panel (and referee when necessary);

- organising and overseeing the translation, including meeting with the translators to produce a consensus-based translation;
- organising and overseeing the quality assurance of the translation;
- producing the report of the translation process.

2. Translation into target language

Two independent translations into the target language of the original document must be produced.

The two translations may be carried out by two individual translators, by two pairs of translators (one translates and the second of the pair reviews the translation) or by two independent panels of translators (with 3-4 members in each panel). If a translator pair or a panel is used, one person should be identified as lead, and be responsible for liaising with the translation coordinator. The two individuals, pairs or panels may not confer with each other until each has produced their translation.

Translators are selected according to the following criteria:

- native speaker of the target language;
- at least one (individual, pair or panel) must be headache or medical expert(s)
- (ideally, the other is a professional translator or bilingual person, pair or panel skilled in language/linguistics, such as a teacher or journalist; if no such translator is available, then a second headache or medical expert [individual, pair or panel] may be used).

Translators are instructed to:

- keep translations simple, avoiding technical language, so that the documents can be understood by lay people of average reading ability;
- make semantic and conceptual translations (rather than literal), so that the meanings of the words and phrases remain as in the original document;
- keep a record of any parts that they found difficult to translate.

3. Production of a consensus-based translation

The coordinator works with the two translators, or the leads of the translation pairs or panels, to reconcile differences between the two translations and produce a consensus-based translation. There are three steps to this process:

- the translators each send their translations to the coordinator;
- the coordinator makes an initial comparison of the two translations and highlights and records any parts of them that are substantially different;

- the coordinator and translators (or leads) meet (or, alternatively, hold a teleconference) to discuss these parts and any other problem areas, agreeing through consensus on one translation.

If the translators cannot reach a consensus on any part, the coordinator, if a native speaker, makes the final decision. If the coordinator is not a native speaker, the referee is called upon to make the final decision.

4. Quality assessment

a) Linguistic review

One assessor is selected according to the following criteria:

- a lay person (not medically qualified and not a researcher);
- a native speaker of the target language (and, ideally, a resident of the relevant country) with good understanding of linguistic factors (such as grammar, readability) but not necessarily bilingual.

The assessor is instructed:

- that the document is to be understood by lay people of average reading ability;
- to assess the consensus-based translation for readability, grammatical correctness and cultural suitability;
- to keep a record of his/her comments and send these to the coordinator.

b) Target audience review

A second quality assessment judges suitability for the intended audience. It is carried out by a review panel of six people selected according to the following criteria:

- affected by headache disorders;
- native speakers of the target language and not necessarily bilingual.

Each panel member assesses the consensus-based translation individually, without reference to the others, sending comments to the coordinator.

c) Production of final quality-assured translation

Minor changes suggested by the assessor or panel members may be implemented by the coordinator (in consultation if necessary with the referee).

When substantial changes are suggested, the coordinator must liaise with the translators, and referee if necessary, in order to agree on an alternative

translation. If substantial changes are agreed, the quality of the new translation should be re-assessed by the same processes.

5. Report of the translation process

The coordinator should produce a report in English on the translation process, documenting the details (qualifications and experience) of the translators, referee, assessors and review panel members. Furthermore, the report will contain:

- the original document;
- the two first translations, the consensus-based translation, any other intermediate versions and the final translation;
- a record of any substantial difficulties encountered during the translation (difficulties may include problematic words or parts of the document that were difficult to translate, points of disagreement and alternatives, or any aspects on which it was difficult to achieve consensus or that were highlighted during the quality assessment of the translation).

The report is to be sent to LTB (mail@l-t-b.org), addressed to the Company Secretary.

Resolving problems

Any problems with or queries about this translation process should be addressed to LTB (mail@l-t-b.org).
