# Supplementary material for: Aids to management of headache disorders in primary care (2nd edition): on behalf of the European Headache Federation and Lifting The Burden: the Global Campaign against Headache
Source: J Headache Pain. 2019 May 21;20(1):57. doi: 10.1186/s10194-018-0899-2 (PMC6734476; doi:10.1186/s10194-018-0899-2)
Supplement: Supplementary file 30 — Translation, and the preservation of original meaning, of materials developed to improve headache management: Translation protocol for hybrid documents (2nd edition). (PDF 177 kb) [file 10194_2018_899_MOESM30_ESM.pdf]

Lifting The Burden

in official relations with
the World Health Organization

The Global Campaign against Headache

30. Translation protocol for hybrid documents

(2nd edition: 2018)

These guidelines have been developed by *Lifting The Burden* (LTB).

They are for the translation of documents (referred to as "hybrid documents") produced for the Global Campaign against Headache and aimed at people with headache but to be used in support either of clinical practice or of research

(such as questionnaires, diaries, survey instruments).

Translations of all hybrid documents for the Global Campaign against Headache should follow these guidelines in order to ensure high-quality translation and be approved by LTB.

Procedure

Translation should follow six steps.

1. Coordination of the translation

A translation coordinator, who oversees but does not carry out the translation, is selected according to the following criteria:

- has technical knowledge (*ie*, understands the concepts underlying the questions or instrument being translated);
- bilingual in English and the target language (ideally a native speaker and a resident of the country of the target language);
- has ability to mediate between different translators and to understand the points of view of lay and professional translators.

If the coordinator is not a native speaker, a referee (native speaker) must be nominated. The referee cannot be involved in the translation process, and is called upon to arbitrate should irreconcilable views amongst translators prevent the production of a consensus-based translation.

The tasks of the coordinator include:

- selecting the forward- and back-translators, assessor and review panel (and referee when necessary);

- liaising when necessary with the document author;
- organising and overseeing the forward- and back-translations, including meeting with the translators first to produce a consensus-based forward-translation and again (when necessary) to resolve discrepancies discovered during back-translation;
- organising and overseeing the quality assurance of the translation;
- producing the report of the translation process.

2. Translation into target language

Two independent forward-translations into the target language of the original document must be produced.

The two translations may be carried out by two individual translators, by two pairs of translators (one translates and the second of the pair reviews the translation) or by two independent panels of translators (with 3-4 members in each panel). If a translator pair or a panel is used, one person should be identified as lead, and be responsible for liaising with the translation coordinator. The two individuals, pairs or panels may not confer with each other until each has produced their translation.

Translators are selected according to the following criteria:

- native speaker of the target language;
- at least one (individual, pair or panel) must be headache or medical expert(s);
- (ideally, the other is a professional translator or bilingual person, pair or panel skilled in language/linguistics, such as a teacher or journalist; if no such translator is available, then a second headache or medical expert [individual, pair or panel] may be used).

Translators are provided by the coordinator with an explanation of the purpose and concepts underlying the elements of the document (obtained, when necessary, from the document author).

Translators are instructed to:

- keep translations simple, avoiding technical language, so that the documents can be understood by lay people of average reading ability;
- make semantic and conceptual translations (rather than literal), so that the meanings of the words and phrases remain as in the original document;
- keep a record of any parts that they found difficult to translate.

3. Production of a consensus-based translation

The coordinator works with the two translators, or the leads of the translation pairs or panels, to reconcile differences between the two translations and produce a consensus-based translation. There are three steps to this process:

- the translators each send their translations to the coordinator;

- the coordinator makes an initial comparison of the two translations and highlights and records any parts of them that are substantially different;
- the coordinator and translators (or leads) meet (or, alternatively, hold a teleconference) to discuss these parts and any other problem areas, agreeing through consensus on one forward translation.

If the translators cannot reach a consensus on any part, the coordinator, if a native speaker, makes the final decision. If the coordinator is not a native speaker, the referee is called upon to make the final decision.

4. Back-translation

One back-translation of the consensus-based forward translation is carried out by one translator selected according to the following criteria:

- a native speaker of English;
- either a headache or medical expert, or a professional or bilingual lay translator skilled in language/linguistic issues.

The back-translation is sent to the coordinator to forward to the original author with a request to compare the original and back-translated versions and assess their conceptual equivalence. If the author believes conceptual equivalence is not maintained, he or she should be asked to explain the reasons to the coordinator.

Following this conceptual comparison, minor amendments may be implemented by the coordinator (in consultation with the referee when appropriate). When substantial discrepancies have been highlighted, the coordinator calls a second meeting (or teleconference) with the forward-translators and back-translator to locate their causes and eliminate them by making changes either to the consensus-based forward-translation or to the back-translation as appropriate.

This process produces the back-checked consensus-based translation.

5. Quality assessment

a) Linguistic review

One assessor is selected according to the following criteria:

- a lay person (not medically qualified and not a researcher);
- a native speaker of the target language (and, ideally, a resident of the relevant country) with good understanding of linguistic factors (such as grammar, readability) but not necessarily bilingual.

The assessor is instructed:

- that the document is to be understood by lay people of average reading ability;
- to assess the back-checked consensus-based translation for readability, grammatical correctness and cultural suitability;

- to keep a record of his/her comments and send these to the coordinator.

b) Target audience review

A second quality assessment judges suitability for the intended audience. A review panel of six people are selected according to the following criteria:

- affected by headache disorders;
- native speakers of the target language and not necessarily bilingual.

Each panel member assesses the back-checked consensus-based translation individually, without reference to the others, sending comments to the coordinator.

c) Production of final quality-assured translation

Minor changes suggested by the assessor or panel members may be implemented by the coordinator (in consultation if necessary with the referee).

When substantial changes are suggested, the coordinator must liaise with the forward-translators, and referee if necessary, to agree on an alternative translation. If substantial changes are agreed, the back-translation process should be repeated and, subsequently, the quality of the new translation should be re-assessed.

6. Report of the translation process

The coordinator should produce a report in English on the translation process, documenting the details (qualifications and experience) of the translators, referee, assessors and review panel members. Furthermore, the report will contain:

- the original document;
- the two forward-translations, the consensus-based translation, the back-translation, the back-checked consensus-based translation, any other intermediate versions and the final translation;
- a record of any substantial difficulties encountered during the translation (difficulties may include problematic words or parts of the document that were difficult to translate, points of disagreement and alternatives, or any aspects on which it was difficult to achieve consensus or that were highlighted during the quality assessment of the translation).

The report is to be sent to LTB (mail@l-t-b.org), addressed to the Company Secretary.

Resolving problems

Any problems with or queries about this translation process should be addressed to LTB (mail@l-t-b.org).
